# Genomes of the Bacterial Endosymbionts of Carrot Psyllid *Trioza apicalis* Suggest Complementary Biosynthetic Capabilities

**DOI:** 10.1007/s00284-025-04119-y

**Published:** 2025-02-20

**Authors:** Sarah Thompson, Jinhui Wang, Thomas Schott, Riitta Nissinen, Minna Haapalainen

**Affiliations:** 1 The New Zealand Institute for Plant and Food Research Limited, Lincoln, New Zealand; 2 College of Plant Protection, Hebei Agricultural University, Lekai South Street 2596, Baoding, 071001 Hebei China; 3 Leibniz Institute for Baltic Sea Research, Seestraße 15, 18119 Rostock, Germany; 4 Department of Biology, University of Turku, 20014 Turku, Finland; 5 Department of Agricultural Sciences, University of Helsinki, P. O. Box 27, 00014 Helsinki, Finland; 6 Natural Resources Institute Finland, Latokartanonkaari 9, 00790 Helsinki, Finland

## Abstract

Carrot psyllid *Trioza apicalis* is a serious pest of cultivated carrot and also a vector of the plant pathogen ‘*Candidatus* Liberibacter solanacearum’ (Lso). To find out whether *T. apicalis* harbours other species of bacteria that might affect the Lso infection rate, the bacterial communities and metagenome in *T. apicalis* were studied. Lso haplotype C was detected in a third of the psyllids sampled, at different relative amounts. Surprisingly, *T. apicalis* was found to harbour only one secondary endosymbiont, a previously unknown species of gamma proteobacterium endosymbiont (Gpe), beside the primary endosymbiont ‘*Candidatus* Carsonella ruddii’ (CCr). The relative abundancies of these two endosymbionts were approximately equal. The genomes of CCr, Gpe and Lso were assembled from a *T. apicalis* metagenome sample. Based on the 16S rRNA gene, the closest relative of Gpe of *T. apicalis* could be a secondary endosymbiont of *Trioza magnoliae*. The 253.171 kb Gpe genome contains all the tRNA and rRNA genes and most of the protein-coding genes required for DNA replication, transcription and translation, but it lacks most of the genes for amino acid biosynthesis. Gpe has no genes encoding cell wall peptidoglycan synthesis, suggesting it has no cell wall, and could thus live as an intracellular endosymbiont. Like the CCr of other psyllids, CCr of *T. apicalis* retains a broad amino acid biosynthetic capacity, whilst lacking many genes required for DNA replication and repair and for transcription and translation. These findings suggest that these two endosymbionts of *T. apicalis* are complementary in their biosynthetic capabilities.

**Supplementary Information:**

The online version contains supplementary material available at 10.1007/s00284-025-04119-y.

## Introduction

Cultivated and wild carrots (*Daucus carota sativus* and *D. carota carota*) are the main host plants of the carrot psyllid *Trioza apicalis* Förster, a pest causing major damage to the carrot crops in northern and central Europe [26, 37]. This psyllid is univoltine and overwinters on coniferous trees, preferably on Norway spruce (*Picea abies*) [22]. *T. apicalis* was found to harbour and transmit the plant pathogen ‘*Candidatus* Liberibacter solanacearum’ (Lso), associated with carrot leaf discolouration symptoms [32, 38].

Psyllids (Psylloidea, Hemiptera) feed on plant phloem sap, which contains sugars but does not have sufficient amounts of essential amino acids. Thus, the phloem sap-feeding insects are dependent on their primary endosymbiont bacteria to provide essential amino acids [12]. Phylogenetic data suggests that the mutualistic association of psyllids and whiteflies with their primary endosymbiont bacteria was established already in their common sternorrhyncha ancestor, approximately 280 million years ago [45]. In all the psyllid species studied, the obligatory primary symbiont is ‘*Candidatus* Carsonella ruddii’ (CCr) [36, 49]. Within the psyllid body, CCr is enclosed in bacteriocytes that are specialised insect cells in an organ called the bacteriome [49]. CCr is transovarially transmitted to the next generation of psyllids, and the congruent phylogenies of CCr and psyllid species suggest that this endosymbiont has continuously evolved together with the psyllid hosts [9, 49].

Most of the psyllid species studied harbour at least one other endosymbiont in addition to CCr, and the different endosymbionts co-residing in the same host may have developed metabolic complementarity between each other. An example of this kind of evolution was recently shown for *Cacopsylla* endosymbionts CCr and an Enterobacteriaceae bacterium ‘*Candidatus* Psyllophila symbiotica’ [11]. Whilst CCr produces most of the essential amino acids, ‘*Ca.* Psyllophila symbiotica’ can provide vitamins and carotenoids, but has lost the amino acid biosynthesis genes, except for genes complementing the tryptophan biosynthesis pathway of CCr [11]. Similarly, the enterobacterial secondary endosymbionts of *Heteropsylla cubana* and *Ctenarytaina eucalypti* have lost most of the essential amino acid biosynthetic pathways and also some of the tricarboxylic acid cycle components [43]. Moreover, transcriptome analysis of *Pachypsylla venusta* body, bacteriome cells and CCr endosymbiont revealed that the bacteriome cells had significantly different gene expression levels compared to the rest of the body, showing elevated expression levels of the genes complementing the incomplete metabolic pathways found in CCr [44].

The loss of many genes encoding the biosynthetic pathways that are essential for free-living bacteria has resulted in a reduced genome size in the obligate endosymbionts of insects [29, 30]. These intracellular endosymbionts seem to have an enhanced genomic instability, which is mainly due to enhanced mutation rate [19]. Their genome nucleotide composition is biassed to be more AT-rich, in comparison to the related free-living bacterial species. The CCr genome is extremely reduced in size, only 160 kb [33], whilst the secondary endosymbionts of psyllids usually have a genome size varying from 0.4 to 4 Mb, and may encode additional functions like toxin production [34].

In addition to the primary and secondary endosymbionts, psyllids can also harbour other bacteria that can play different roles in the psyllids’ microbiome and in the interaction with the host plants, varying from beneficial to parasitic interactions [35, 36]. For ‘*Ca*. Liberibacter’ species an auxiliary role in feeding has been suggested, as the Lso haplotypes A and B harboured by *Bactericera cockerelli* were found to delay the defence response of the host plant tomato against the psyllid infestation [18]. Apart from the assembly and analysis of the genome of Lso haplotype C [52], the bacteria living inside the carrot psyllid *T. apicalis* have not been studied at community or genomic level.

The aims of this study were (i) to analyse the internal bacterial community of *T. apicalis* and identify the number and relative abundance of potential endosymbiotic bacteria, in both Lso positive and Lso negative individuals, and (ii) to study the most abundant endosymbiotic bacteria at the genome level. The bacterial community of *T. apicalis* was analysed by 16S rRNA sequencing and the genomic sequences of three bacterial species were assembled using metagenomic approach. Comparative genetic and genomic analyses were carried out with these three bacterial genomes.

## Materials and Methods

### Sample Preparation

Carrot psyllids (*T. apicalis*) for bacterial community analysis were captured from carrot leaves in an infested carrot field in Laitila (60°52’ N, 21°41’ E), Southwest Finland, in July 2017. Each psyllid was checked under stereomicroscope to confirm the species, sex and integrity, and 69 psyllids were stored at − 20 °C in tubes. Before DNA extraction, the psyllids were thoroughly cleaned to remove dust and contaminating microbial DNA attached to the outer surfaces. First, the psyllids were individually contained in small bags (10 mm × 25 mm), specially made of thin nylon mesh and autoclaved before use. The bags with psyllids were stacked inside a tea ball strainer (stainless steel, autoclaved) and submerged in 1% sodium hypochlorite solution for 3 min, with stirring, then in 0.3% Triton X-100 (Sigma) for 1 min, with stirring, and finally rinsed three times with sterile distilled water. This protocol was modified from the one used by Hall et al. [17].

### DNA Extraction

Each psyllid was placed in 100 µL of DNA extraction buffer (2% CTAB, 1.4 M NaCl, 20 mM EDTA, 100 mM Tris–HCl pH 8.0, 0.2% 2-mercaptoethanol) in a 1.5 mL tube and crushed with a sterile micro-pestle. Heat-sterilised extra-pure sea sand (Merck) was added to the tubes to help the homogenisation. Then another 400 µL of extraction buffer was added and the samples were heated at 65 °C for 20 min, with mixing twice by inverting the tubes. After adding 500 µL of chloroform octanol mixture (24:1), the samples were mixed by vortex for 10 min, followed by phase separation by centrifugation for 5 min at 9400 × g at room temperature. The upper phase was moved into a new tube and the DNA was precipitated with 1 mL of ethanol, at − 20 °C for 2 h or longer. After centrifugation at 16,000 × g for 20 min, at 4 °C, the supernatant was removed and the precipitate washed once with 1 mL cold 70% ethanol. The precipitates were air-dried at room temperature and then re-dissolved in 50 µL sterile nuclease-free water at 4 °C. The DNA concentration was measured with NanoDrop Lite spectrophotometer (Thermo Fisher Scientific). The samples were stored at − 20 °C.

### Quantitative PCR

The psyllid DNA samples were diluted in sterile nuclease-free water. To determine the PCR E-values, a dilution series was prepared of an Lso positive *T. apicalis* control sample. Each 15 µL reaction contained 7.5 µL SYBR Green I Master Mix (2×) (Roche, Basel, Switzerland), forward and reverse primers at 500 nM concentration, and 1 ng of sample DNA or 5 µL of a control DNA sample. The PCR programme, with initial denaturation at 95 °C for 5 min and 45 cycles of denaturation at 95 °C for 10 s, annealing at 54 °C for 10 s and elongation at 72 °C for 10 s, followed by melting curve analysis, was run on a LightCycler 480 qPCR machine (Roche). All the reactions were run in triplicates, in parallel with Lso 16S rRNA gene specific primers OA2 [27] and Lib-Rev203 [38] and *T. apicalis* 18S rRNA gene specific primers Tza-2F/ Tza-2R [38], to determine the relative Lso titre in each sample by the Pfaffl method [40]. For Lso, the qPCR detection limit (Ct = 40) was used as Lso negative reference value, and for *T. apicalis* 18S the average Ct value of all the tested psyllid samples was used as the reference level.

### Bacterial 16S Sequencing and Analysis

Eighteen samples of DNA from carrot psyllids, six with no detectable presence of Lso, six with a moderate titre and six with a high titre of Lso, were chosen for microbiome analysis. A fragment of bacterial 16S rRNA gene was amplified following the protocol previously described [39], using primers 799f/1492r [6] and M13-1062F/ P1-1390R [28] in a nested approach. The nested primers targeting the V6-V8 regions of 16S rRNA gene enable elimination of host mitochondrial amplicons by size fractionation (799f–1492r) and produce an amplicon with high phylogenetic coverage and optimal size for IonTorrent sequencing (1062f–1390r). Primer 1062f was tagged with M13 sequence to enable sample barcoding, as previously described [28]. Both reactions contained 1–2 μL of sample DNA, 1 × PCR buffer, 1 mg/mL of BSA, 0.2 mM dNTPs, 0.3 μM of each primer and 1250 U/mL GoTaq DNA Polymerase (Promega, WI, USA) in a 30 μL reaction volume. In PCR 20 ng of DNA was used as template, and 1 μL of 1:10 diluted amplicons from the PCR were used as template for the nested run. The PCR programme for both the runs was: 3 min denaturation at 95 °C, followed by 25 and 15 cycles (nested PCR) of denaturing at 95 °C for 45 s, annealing at 54 °C for 45 s, and extension at 72 °C for 1 min. Final extension was carried out at 72 °C for 5 min. The samples were barcoded with a third PCR: Amplicons from the nested PCR were diluted 1:5 and amplified using a barcoded M13 adapter as forward primer and 1390r-P1 with adapter A as the reverse primer [28]. PCR mix and conditions were similar as described above, except only 8 cycles were used for amplification. Amplified samples were purified using Agencourt AMPure XP PCR purification system (1.8X sample volume) (Beckman Coulter, CA, USA). Purified samples were quantified with Tape Station 2200 (Agilent, CA, USA) and pooled equimolarly (20 ng of eubacterial amplicon/sample) for sequencing library. The library was size fractionated using Pippin Prep (Sage Science, MA, USA) 2% Agarose gel cassette (Marker B) following the manufacturer’s protocol to select the amplicons within size range of 350–550 bp. Size selected library was diluted to 22 pM, of which 25 μL was used for sequencing using an Ion 316 chip kit V2 BC on Ion Torrent PGM (Life Technologies, CA, USA) at the Department of Biological and Environmental Science, University of Jyväskylä. The sequence reads were processed using a CLC Genomics Workbench 11.0 with a Microbial Genomics Module (Qiagen, Denmark). Raw reads imported from IonTorrent were screened for chimaeras and quality. Chimeric sequences and reads < 150 bp and with Q score < 25 were removed. Good quality reads were clustered into OTUs (operational taxonomic units) at 97% sequence identity using reference-based OTU assignment, using Silva SSU reference 99% database release 312 [41].

### Genomic Sequences of the Endosymbiont Bacteria

Sequencing of the total DNA of one previously obtained Lso positive *T. apicalis* sample, 11-H40 from Haukivuori (62°01’ N, 27°12’ E) in South Savo [16], was performed on Illumina High Seq2000 equipment in the Functional Genomics Unit of the University of Helsinki. Prior to DNA extraction by CTAB method, this psyllid sample had not been surface-sterilised but stored in ethanol at − 20 °C. Kraken analysis against the MiniKraken database [54] was performed on the total microbial DNA reads of the sample 11-H40 to identify the bacteria and viruses present. Reads classified as ‘*Ca*. Carsonella’ or *Enterobacterales* were assembled using SPAdes v3.6.1 (default settings) and reads classified as ‘*Ca*. Liberibacter’ were assembled using SPAdes v3.10.1 [2]. Additionally, all reads were assembled as described before [52] and subsequently binned by abundance and tetranucleotide frequencies using MetaBAT [21]. Resulting bins were taxonomically classified using CAT/BAT [51]. Reads mapping to contigs (bowtie2 with default settings, [25]) from the targeted assembly or from *Enterobacterales* bins were combined and assembled once more. Contigs smaller than 1 kb were discarded. The resulting assembly, Gpe of *T. apicalis*, was manually refined, validated and checked for read pair integrity using Gap5 [4]. The previous assembly of Lso haplotype C FIN114 [52] was used as the scaffold to order the contigs of the Lso FINH40 assembly. The genomes of CCr and Gpe of *T. apicalis* were assembled de novo.

### Genomic Sequence Analysis

Annotation of the assembled bacterial genomes was done through the Prokaryotic Genome Annotation Pipeline (PGAP) at the National Center for Biotechnology Information (NCBI). Biosynthetic capacities of the endosymbionts were assessed by pathway analyses. Mapping tools provided by the Kyoto Encyclopedia of Genes and Genomes (KEGG) [20] were used to analyse the completeness of metabolic pathways. Genome organisation of the assembled endosymbiont genomes was compared with the previously sequenced genomes using the multiple genome alignment tool Mauve [10].

### Phylogenetic Analyses

Multi-locus phylogenetic trees of Ccr and insect endosymbiont bacteria related to the Gpe of *T. apicalis* were generated using Randomized Axelerated Maximum Likelihood (RAxML) method [46]. Sequences of single copy orthologs identified using OrthoFinder [14] were first aligned by MUSCLE [13] and then concatenated for analysis. The full-length 16S rRNA sequences of Ccr and the Gpe of *T. apicalis* were compared by Blastn tool against the nucleotide database at the NCBI. For the Gpe, 16S rRNA sequences of selected endosymbionts and reference species were aligned by MUSCLE and the final dataset had a total of 1209 nucleotide positions. The 16S rRNA phylogeny analysis was conducted in MEGA7 [23], using the Maximum Likelihood method.

## Results

### Lso Infection Levels in the *T. apicalis* Samples

Of the adult carrot psyllids captured directly from carrot leaves in a field in Laitila, the majority (59%) were males, and the rest were egg-laying females. DNA extraction was successful from 62 samples, and 23 (37%) of these samples were confirmed to be Lso positive by quantitative PCR. The experimental E-values with Lso-specific primers and psyllid-specific primers were 1.92 and 1.95, respectively, indicating high efficiency. Six psyllids that had a very high relative titre of Lso (Lso/psyllid target gene ratio > 30,000) were chosen for the bacterial community analysis. As all of these psyllids happened to be male, six males with moderate Lso titre (target gene ratio from 1,000 to 30,000) and six males with no detectable amount of Lso (threshold cycles ≥ 40) were chosen for the bacterial community analysis, to allow comparison of the relative amounts of the endosymbiont bacteria between the three groups (Table 1). The total DNA concentration in these 18 samples was on average 18.53 (SD 3.49) ng µL^−1^.

**Table 1:** Relative amounts of ‘*Candidatus* Liberibacter solanacearum’ (Lso), ‘*Candidatus* Carsonella ruddii’ (CCr) and gamma proteobacterium endosymbiont (Gpe) in the samples of male carrot psyllids (*Trioza apicalis*) chosen for microbiome analysis

Sample group	Relative Lso titre^a^	Lso / CCr ratio^b^	Lso / Gpe ratio^b^	Gpe / CCr ratio^b^
High Lso titre (> 30,000)	98,143 (± 43,889)	12.23 (± 12.77)	12.55 (± 13.89)	1.05 (± 0.83)
Moderate Lso titre (1000–30,000)	5469 (± 3226)	0.85 (± 0.58)	1.26 (± 1.05)	0.79 (± 0.38)
No Lso detected	0	0	0	1.24 (± 0.39)

^a^Determined by quantitative PCR, average (± standard deviation). ^b^Based on the number of 16S OTUs, average (± standard deviation)

### Bacterial Community of *T. apicalis*

**Fig. 1:**
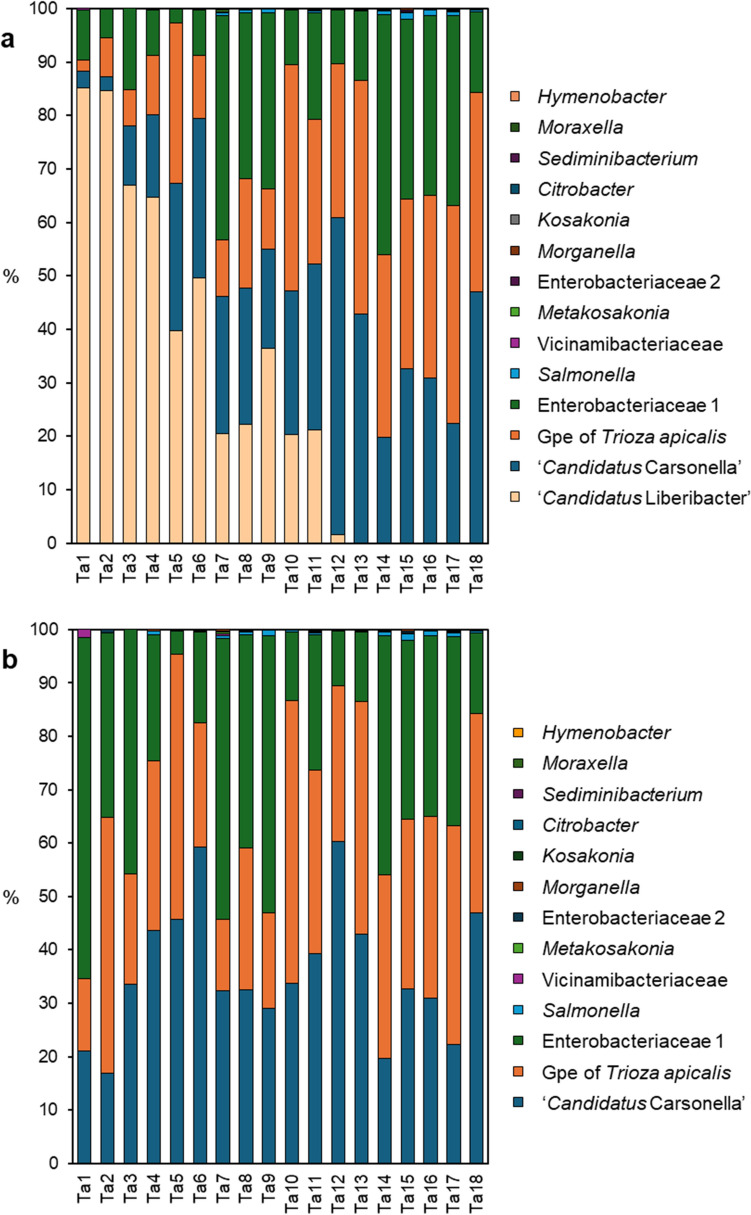
Proportions of different bacterial taxa inside *Trioza apicalis* male individuals. **a** Composition of the bacterial community with ‘*Candidatus* Liberibacter solanacearum’ (Lso) present in twelve of the samples and **b** the bacterial community after omitting the Lso reads. Only the eleven most abundant bacterial taxa are shown

Bacterial 16S rRNA metabarcoding of 18 surface-sterilised carrot psyllid samples from Laitila revealed the presence of a total of 108 OTUs and 26 bacterial genera, mostly representing the bacterial phylum Proteobacteria. The number of bacterial 16S reads obtained per sample, after filtering, ranged from 514 to 2056 and the total number of reads analysed was 21,845. On average, Lso (alphaproteobacteria) was the relatively most abundant taxon, followed by CCr, Gpe of *T. apicalis* and the bacterial OTU the family Enterobacteriaceae (Fig. 1a). Collectively, these four taxa comprised over 98% of the total bacterial community. Similarly to the other psyllids [36], the relative proportion of CCr reads was high in *T. apicalis*, and so was the proportion of Gpe reads. When the Lso reads were excluded from the data, the bacterial communities in the groups of psyllids with different levels of Lso colonisation did not significantly differ from each other (Fig. 1b), which was verified by PERMANOVA and PcoA (results not shown). Calculated from the proportions of summed reads, the ratio of Gpe to CCr was on average 1.03 (SD 0.60) (Table 1), and the ratio was not significantly different in the psyllid groups with a high or moderate Lso titre or in psyllids without a detectable amount of Lso (two-sample t-test).

Kraken analysis of the microbial metagenome of *T. apicalis* sample 11-H40, collected from Haukivuori, detected presence of Ccr and Lso together with its prophage. The metagenome analysis also showed hits to various other bacteria, mainly insect endosymbionts belonging to the order Enterobacterales (Supplementary Data S1 and S2). However, as the Kraken analysis was suggesting hits to a large number of different gamma proteobacterial S-endosymbionts, it was not possible to identify the S-endosymbiont of *T. apicalis* as any of the previously known species, nor to tell how many species of gamma proteobacterium endosymbionts there were. The fourth major group detected in the bacterial community analysis, representing unclassified enterobacteria, was almost completely absent in the metagenome DNA sample 11-H40 (Supplementary Data S2).

### Genomic Assemblies of Lso, Ccr and Gpe of *T. apicalis*

The Lso haplotype C genomic assembly FINH40 contains 1,064 genes, of which 982 are protein-coding genes (Table 2). The assembly shows differences in the core genome organisation in comparison to the previously published Lso haplotype C assembly FIN114, derived from a psyllid sample from Forssa 2012 colony [52]. In these genomic assemblies two large regions are located differently in relation to the other regions (Supplementary Data S3). Like FIN114, the assembly FINH40 contains genes for flagellar biosynthesis, suggesting motility, and for Flp family type IVb pilus biosynthesis, enabling attachment.

**Table 2:** Features of the genomic assemblies of the three sequenced endosymbionts

Feature	‘*Candidatus* Liberibacter solanacearum’	‘*Candidatus* Carsonella ruddii’	Gamma proteobacterium endosymbiont of *Trioza apicalis*
Assembly size, nucleotides	1,185,167	166,761	253,271
Number of contigs	18	1	1
Genes, total	1064	229	260
Protein coding genes	982	198	223
RNA genes	57	30	36
rRNAs	3, 3, 3	1, 1, 1	1, 1, 1
tRNAs	45	27	30
Pseudogenes	25	1	1
Genome coverage	15x	300x	6000x

The primary endosymbiont of *T. apicalis*, CCr strain TA, has an extremely reduced genome size, only 166.761 kb (Table 2). Despite the reduced size, the CCr TA genome still contains 198 protein-encoding genes and 30 RNA-encoding genes. The base composition is very AT-rich, with only 15.1% G + C, which leads to a bias in amino acid codons in favour of the AT-rich codons.

The assembled genome sequence of Gpe of *T. apicalis* is 253.171 kb long, and the DNA GC-content is 20.4%. This genomic assembly contains all the tRNAs, rRNAs and ribosomal protein genes (Table 2) as well as genes encoding other proteins required in translation and DNA replication and transcription. The assembly also includes genes encoding proteins involved in the energy metabolism, protein secretion through Sec system and transporters for the import of nutrients and efflux of cations through the cytoplasmic membrane (Supplementary Data S4). This suggests that the assembly represents the whole or almost whole genomic sequence. All the metagenome contigs were double-checked and taxonomically annotated to find more contigs fitting the Gpe assembly, however, those potential candidate contigs that showed similar abundance to the Gpe assembly were clearly of eukaryotic origin. Despite the very high genome coverage (6000x), the assembly could not be closed to form a circular chromosome, which suggests that either the chromosome is linear—which is unusual for a bacterium—or that a piece of sequence of unknown length and origin (bacterial, phage or eukaryotic) has not been identified yet. The organisation of Gpe genome is different from the related Enterobacteriaceae endosymbionts of *Cacopsylla picta* and *Cacopsylla pyricola* and from the ant endosymbiont ‘*Candidatus* Westeberhardia cardiocondylae’ (Supplementary Data S5). In comparison to the genome of ‘*Ca.* W. cardiocondylae’, which is twice as long as the Gpe genome, Gpe has lost many genes, including all the genes encoding enzymes required for peptidoglycan cell wall biosynthesis and the cell rod shape -determining proteins. Also the genes for outer membrane protein (omp) assembly factors and omp chaperons are missing from Gpe, as well as the protein factors needed for controlling cell volume under different osmolarity conditions.

The genome assemblies of ‘*Ca.* Liberibacter solanacearum’ FINH40, ‘*Ca.* Carsonella ruddii’ strain TA and the gamma proteobacterium endosymbiont of *T. apicalis* were deposited at GenBank as biosamples SAMN10723055, SAMN14054108 and SAMN14054176, respectively, under the bioproject PRJNA514334, and nucleotide sequence accession numbers JACEEQ000000000, CP059138 and CP066222.

### Phylogenies of CCr TA and Gpe of *T. apicalis*

**Fig. 2:**
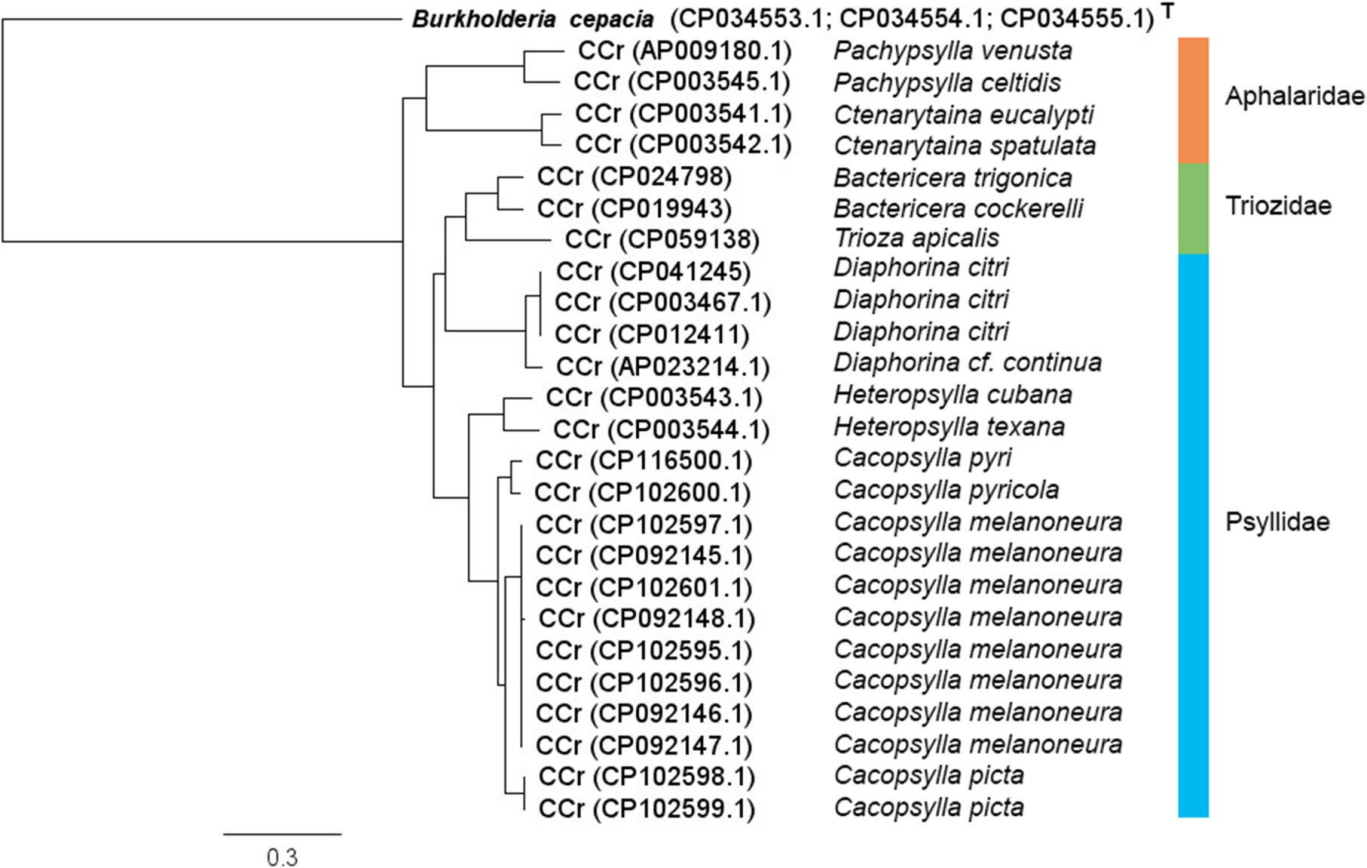
An RAxML phylogenetic tree based on 59 single copy orthologs from 25 ‘*Candidatus* Carsonella ruddii’ (CCr) strains and the betaproteobacteria type species *Burkholderia cepacia* type strain ATCC 25416 as the outgroup. Species of the psyllid hosts of the CCr strains are marked on the right side of the tree and the psyllid families are shown on the right. Scale bar refers to a phylogenetic distance of 0.3 nucleotide substitutions per site

The average nucleotide identity (ANI) analysis showed that the CCr genomes even from closely related psyllid species were diverse, challenging the suggested 95–96% bacterial species demarcation level for ANI (data not shown). However, the average amino acid identity (AAI) analysis showed that the relatedness between the CCr protein-coding genes is concordant with the taxonomy of the psyllid hosts (Supplementary Data S6). Based on the 16S rRNA gene sequence comparison, the CCr strains from *T. magnoliae* (95.03% identity) and *T. eugeniae* (94.43% identity) are the most closely related ones to CCr TA, followed by CCr from *T. urticae* (94.35% identity), *Bactericera trigonica* (94.23% identity) and *B. cockerelli* (from 94.09 to 94.16% identity). However, the 16S rRNA gene phylogeny of CCr strains is not well resolved, due to numerous random mutations in the variable regions (data not shown). Instead, comparison of the protein-coding gene regions of CCr (Supplementary Data S7) gives a phylogeny tree that agrees well with the psyllid phylogeny and with the result of the AAI analysis (Fig. 2, Supplementary Data S6).

**Fig. 3:**
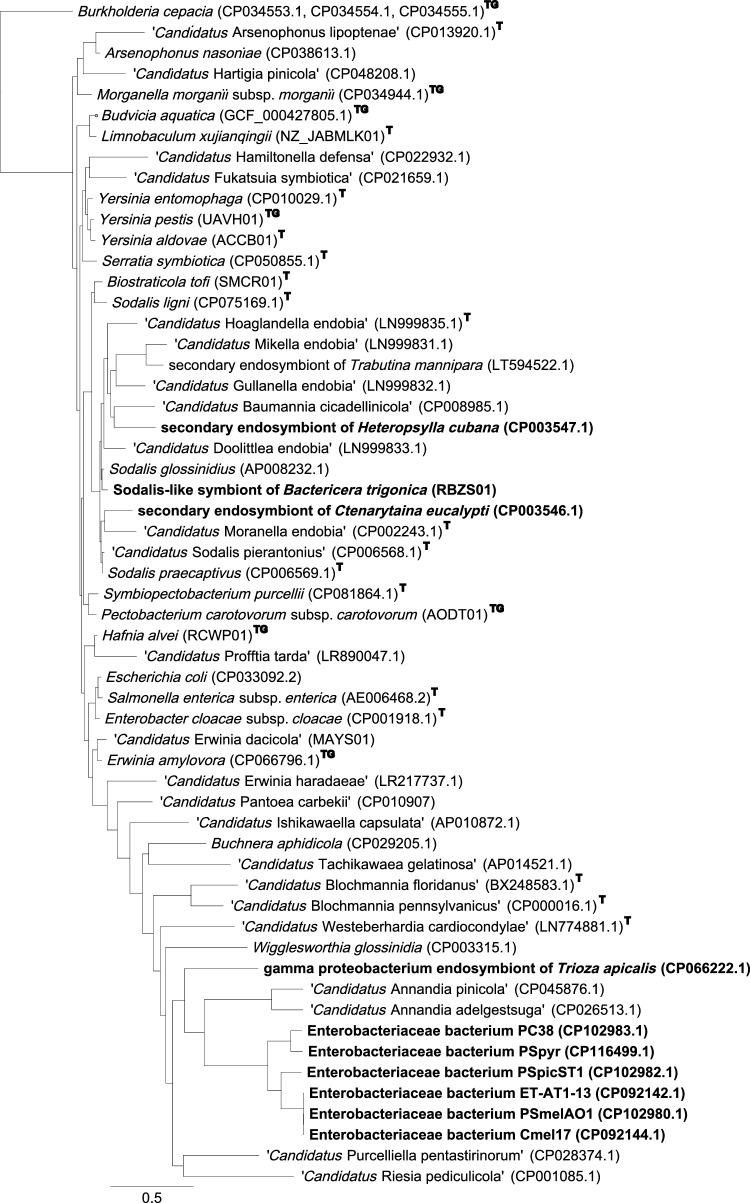
Multi-locus phylogenetic tree generated from the analysis of 56 Enterobacterales genomes using the betaproteobacteria type species *Burkholderia cepacia* type strain ATCC 25416 as an outgroup. Amino acid sequences of 41 single copy orthologs were aligned by MUSCLE and concatenated, and the tree was generated using RAxML method. Scale units are substitutions per site. T indicates a type species and TG indicates a type genus. Endosymbionts of psyllids are presented in bold

Based on BLASTn analysis against the GenBank nucleotide database, the 16S rRNA sequence showed that the bacteria with the highest sequence similarity to the Gpe of *T. apicalis* are a secondary endosymbiont of *T. magnoliae* (GenBank AF077607.1) and the endosymbionts of lice *Haematopinus apri* (GenBank LC706254.1) and *Haematopinus suis* (GenBank KX146200.1), with nucleotide identities 90.93%, 90.57% and 90.35%, respectively. Alignment of the endosymbiont 16S rRNA gene sequences from different insects revealed many single nucleotide variations and also larger deletions and insertions. Exclusion of the hypervariable N-terminal region was required to obtain a solid alignment, and still the only bacteria clustering closely together with Gpe were the S-endosymbiont of *T. magnoliae* and the endosymbionts of two lice species (Supplementary Data S8). The translated amino acid sequences of protein-encoding gene regions of Gpe and a range of insect endosymbionts for which genome sequences were available were compared, and selected genomes representing the seven Enterobacterales families were analysed. Comparison of 41 gene loci (Supplementary Data S7) suggests that of the endosymbiont species, the ones most closely related to Gpe of *T. apicalis* are the endosymbionts of the *Cacopsylla* species *C. melanoneura*, *C. picta* and *C. pyricola*, and the adelgid endosymbionts ‘*Candidatus* Annandia’ species (Fig. 3).

### Complementarity of the Biosynthetic Capacities of the Two Endosymbionts of *T. apicalis*

**Fig. 4:**
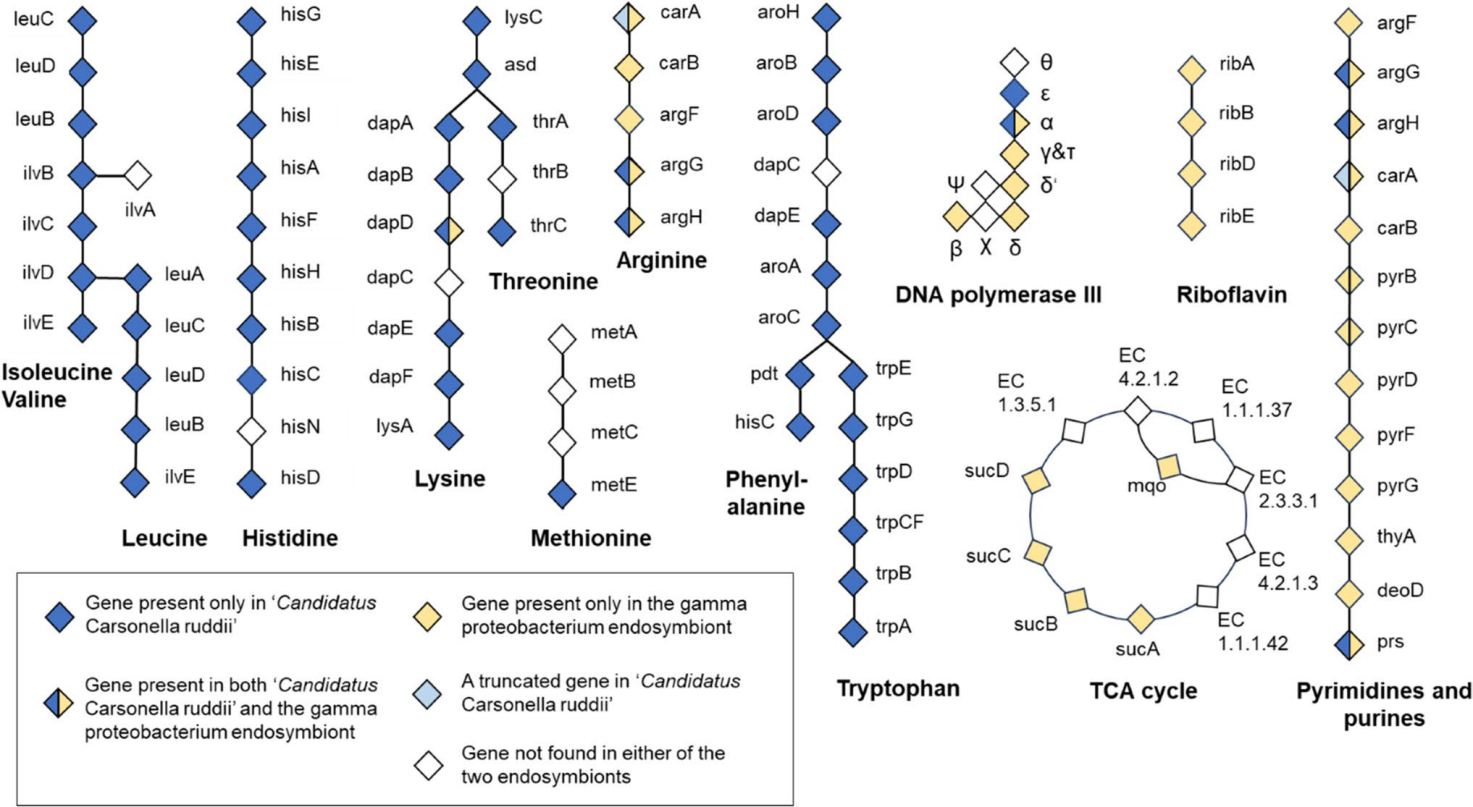
Complementary and incomplete biosynthetic pathways in ‘*Candidatus* Carsonella ruddii’ and the gamma proteobacterium endosymbiont of *Trioza apicalis*. Enzyme Commission (EC) numbers for the enzymes and additional information are provided in Supplementary Data S4

Like the CCr found in other psyllids, CCr TA is lacking the genes encoding bacterial cell wall components and extracellular structures. CCr TA also lacks genes for transporters and protein secretion as well as ribosome biogenesis factors (Supplementary Data S4). Instead, the small genome of CCr TA is tightly packed with genes encoding the enzymes needed for amino acid biosynthesis. Based on the pathway analysis in KEGG, CCr TA has the capacity of de novo biosynthesis of at least six essential amino acids (histidine, valine, leucine, isoleucine, phenylalanine and tryptophan) and probably also of lysine and threonine (Fig. 4). The genes *dapC*(*ArgD*) and *thrB* of those amino acid biosynthesis pathways were not identified, and thus it is possible that those enzymes could be provided by other bacteria or by the host bacteriome cells. Like the other CCr, CCr TA genome encodes the enzyme 5-methyltetrahydropteroyltriglutamate–homocysteine S-methyltransferase (MetE) that catalyses the formation of methionine from 5-methyltetrahydrofolate and homocysteine. CCr TA also carries genes *argG* and *argH* required for biosynthesis of arginine from citrulline, but lacks the gene *argF* encoding ornithine transcarbamylase, required for production of citrulline from ornithine. However, Gpe has the capacity to convert glutamine to carbamoyl phosphate, which is the precursor for arginine biosynthesis, and carries the other arginine biosynthesis pathway genes as well. Apart from the genes *carA*, *carB*, *argF*, *argG* and *argH*, no other amino acid biosynthesis genes were identified in the genome of Gpe of *T. apicalis*. The enzymes required for arginine biosynthesis are also required in the de novo biosynthesis of pyrimide and purine nucleotides, and Gpe carries genes of this pathway (Fig. 4). Neither CCr TA nor Gpe appears to be capable of synthesizing the group B vitamins: no components of the pathways for pyridoxin (vitamin B6) or biotin (vitamin B7) synthesis were identified, and in Gpe the de novo synthesis pathway for riboflavin (vitamin B2) is incomplete, lacking two key enzymes (FMN hydrolase YbjI and riboflavin synthase RibC).

Surprisingly, the genes encoding DNA-directed RNA polymerase subunit alpha (rpoA) and subunit beta’ (rpoC), which are intact in the CCr genomes derived from *B. cockerelli* and *B. trigonica*, are both broken in CCr TA by introduction of a stop codon, separating the C-terminal domain from the rest of the coding region. In both cases the sequence encoding the C-terminal domain of the protein is also in a different reading frame than the larger part of the coding region. Moreover, CCr TA lacks the enzymes required for nucleotide biosynthesis and the protein factors required in transcription elongation, pausing and termination, as well as ribosome biogenesis, which suggests that these factors are provided for CCr from another source. Indeed, the genome of Gpe of *T. apicalis* carries intact genes for all the subunits of DNA-directed RNA polymerase and for the other factors required for nucleotide biosynthesis, transcription and translation (Supplementary Data S4). As previously noted [48], most of the subunits of DNA polymerase III are missing from the CCr genome, and this also applies to CCr TA: only the genes for subunits alpha and epsilon, and a truncated and degenerated version of the subunit beta were found. This deficiency could be complemented by Gpe which carries genes for all the DNA polymerase III subunits. Altogether, the results of genomic analyses support the view that CCr TA could provide most of the essential amino acids required by the psyllid host and by Gpe, whereas Gpe could produce nucleotides, DNA replication factors, ribosome biogenesis factors, most of the transcription and translation factors, Fe-S cluster subunits, transporters and co-factors that the CCr TA genome does not encode.

## Discussion

Bacterial community analysis of *T. apicalis* samples from Laitila, Finland, showed that all the psyllids harboured CCr, the primary endosymbiont common to all the psyllid species studied so far. In addition, a novel species of gamma proteobacterium endosymbiont (Gpe) and an unclassified Enterobacteriaceae were abundant in all the samples. In those *T. apicalis* individuals that had a high Lso colonisation level, Lso was the most abundant bacterial species. The bacterial OTUs belonging to unclassified Enterobacteriaceae and the other (minor) bacterial OTUs detected by the community analysis are likely to be derived from bacteria residing in the psyllid alimentary canal. In the *T. apicalis* metagenome DNA samples 11-H40 and FIN114 [this study, 21] bacterial taxa corresponding to the unclassified Enterobacteriaceae OTUs were not detected. However, as the 16S metabarcoding samples and the samples for metagenome assembly were sampled at different locations and in different years, it is possible that these enterobacteria might have been acquired from the host plant microbiome in Laitila at the time of sampling psyllids for the microbiome analysis. No antagonistic or synergistic relationships were identified between Lso and the other bacterial taxa on the basis of relative abundancies. This could be due to the different location of the different bacteria inside the psyllid body: based on the studies on other psyllids in family Triozidae, the obligate endosymbionts are confined in specialised cells (bacteriocytes) and Lso invades multiple tissues [7, 15], whilst the other bacteria reside in the alimentary canal. However, a competitive interaction has been suggested between Lso and a densovirus (BtDNV) infecting the psyllid *B. trigonica* tissues [15]. As no densovirus was detected in the Kraken analysis of *T. apicalis* 11-H40, it remains open whether this kind of interaction could take place in *T. apicalis*.

Apart from CCr, the major bacterial taxa in *T. apicalis* were different from the other economically significant Lso vectors *B. cockerelli* and *B. trigonica*. The bacteria most commonly found in *B. cockerelli*, in addition to CCr, are *Wolbachia*, *Acinetobacter* sp., *Pseudomonas* sp., Enterobacteriaceae and Lso [1]. However, the relative abundances of different bacterial species in *B. cockerelli* were found to vary depending on the location of collection within the United States of America (USA) [1]. The northwestern *B. cockerelli* colonies did not carry *Wolbachia*, which was suggested to have a connection with their lower Lso titre and transmission rate on potato [8]. *B. trigonica* psyllids from colonies originating from Israel harboured CCr, *Sodalis* and *Spiroplasma* endosymbionts, whereas they contained no *Wolbachia* or ‘*Candidatus* Arsenophonus’ [15]. The *Sodalis*-like endosymbiont of *B. trigonica* had lost most of the amino acid biosynthesis genes [15], suggesting a biosynthetic capacity complementary with that of CCr, similar to that found in this study for Gpe of *T. apicalis*. The published genome sequence of the *Sodalis*-like bacterium of *B. trigonica* (GCA_003668825.1, NCBI) contains strikingly many transposase-like insertion sequences, which implies that genome rearrangements may happen frequently. Although ‘*Ca*. Arsenophonus’ has been identified as a potential secondary endosymbiont in *Trioza eugeniae* and *Wolbachia* as a facultative endosymbiont in *Trioza magnoliae* [31], these bacteria were not detected in *T. apicalis.* Instead, *T. apicalis* harbours an endosymbiont that, based on the 16S rRNA gene sequence, seems to be most closely related to a secondary endosymbiont of *T. magnoliae*.

The genome of the Gpe of *T. apicalis* was assembled de novo from the metagenome sequence reads classified as Enterobacterales. The fragments of sequences that had appeared in the Kraken analysis as a batch of hits to multiple different gamma proteobacterial endosymbionts, all turned out to belong to the same genome and could be joined together into one continuous sequence. Based on the genomic sequence of the Gpe of *T. apicalis*, it may represent an unknown species of unclassified Enterobacteriaceae, and thus the name ‘*Candidatus* Triozidicola socius’ (“Triozidae-loving partner”) is suggested for this species, belonging to the family Enterobacteriaceae, order Enterobacterales, class Gammaproteobacteria.

The world-wide distribution of closely related psyllid species and their primary endosymbiont CCr suggests that this symbiotic relationship is very old. On the other hand, incongruences observed between psyllid S-endosymbiont phylogeny and the insect phylogeny suggest that these symbionts may have sometimes been passed between different insect species [17]. In this study, the Gpe of *T. apicalis* was found to be as related to endosymbionts of adelgids as to other psyllid species. Differences observed in the bacterial communities of closely related psyllid species may, in turn, reflect their adaptation to different climate and vegetation zones. As recently shown for whitefly (*Bemisia tabaci*) [42], the phloem-feeding insects can acquire bacteria from the host plant, and after switching to a different host plant species the insect gut-associated bacterial community changes. Whilst the newly acquired bacteria may help the insect to adapt to the new host plant, the acquired bacteria, switching between the plant and the insect, also have to adapt to the new insect host. As an example of a host switch phenomenon between different insects, the evolutionary evidence suggests that the ant endosymbionts ‘*Ca*. Blochmanniella’ spp. were originally acquired from the phloem sap-feeding insects tended by the ants [53]. Thus, the host switches can enhance the bacterial diversity and speciation, in addition to facilitating the spread of the bacteria by different insects.

Similarly to the endosymbionts, Lso does not have a free-living stage in its life cycle, but is switching between two hosts, the psyllid and the plant. When an infected psyllid feeds on the plant phloem fluid, Lso gets into the phloem sieve cells where it lives as an intracellular parasite [38]. Another psyllid may then feed on the infected plant and acquire Lso, which can then move to the salivary gland and colonize it [7], enabling transmission of Lso into new plants. Lso can utilise different psyllids of the families Triozidae and Aphalaridae as hosts [47], and thus infect plants in many different families. As an adaptation into living in the nutrient-rich environments inside psyllids and plants, Lso has a reduced genome size in comparison to the free-living *Rhizobiales*, and has lost e.g. the majority of the amino acid biosynthetic pathways [52].

The differences found in the Lso haplotype C core genome organisation between the new assembly FINH40 and the previous assembly FIN114 [52] could reflect a long-term separation of the psyllid populations. The *T. apicalis* sample 11-H40 was collected from Haukivuori and the sample FIN114 from Forssa (60°49’ N, 23°37’ E). These sampling locations are more than two hundred kilometres away from each other and separated by large lakes. Previously, differences in genome organisation were found between the Lso haplotype A sequences obtained from New Zealand and USA [50]. For the haplotype C genome FIN114, two prophage regions were also assembled, however, this could only be achieved through cloning, due to the flanking repeat-rich sequences [52].

The small genome of the Gpe of *T. apicalis*, with many genes essential for free-living bacteria either missing or been degenerated, suggests a long history of reductive genome evolution. Because of the loss of many genes encoding essential metabolites, this bacterium would be incapable of surviving as an independent organism. Moreover, the finding that Gpe genome contains no genes encoding for cell wall peptiglycan synthesis or the cell rod shape determining proteins suggests that Gpe does not have a cell wall. Thus, it is likely to live as an intracellular endosymbiont. Gpe could be harboured within the same bacteriocytes as CCr, like *Sodalis* and CCr in *B. trigonica* [15], or in separate bacteriocytes, as ‘*Ca*. Sulcia muelleri’ and the gamma proteobacterium endosymbiont of cixiid leafhoppers [5]*.* The very high sequencing coverage seen across all the regions of the assembled Gpe genome implies that any large genomic regions are unlikely to have been missed because of no coverage. Thus, despite its small size and the fact that it could not be closed to form a circular chromosome, the genomic assembly obtained is likely to be near to complete. Linear chromosomes have been previously found in other bacteria—the first proven case was *Borrelia burgdorferi* [3]. Many insect endosymbionts have an extremely compact genome that has undergone a dramatic reduction. For example, the genome size of the leaf beetle endosymbiont ‘*Candidatus* Stammera capleta’ varies from 212.7 to 325.6 kb (GenBank accessions CP043989.1 and CP144853.1), and the genome size of the Enterobacteriaceae endosymbionts of *Cacopsylla* species varies from 221.4 to 237.1 kb (GenBank CP116499.1, CP102980.1). There is evidence that even the extremely reduced genomes of insect endosymbionts are still functional but may need the help from other symbionts and host bacteriome cells [11, 44].

Several genes encoding enzymes required in essential biosynthetic pathways seem to be missing from both CCr TA and Gpe of *T. apicalis*, and it is possible that those enzymes could be provided by the host bacteriome cells. CCr of *Pachypsylla venusta* was previously found to have shared biosynthetic pathways with the host bacteriocytes [44], including the phenylalanine synthesis pathway. Also in CCr TA, this pathway seems to lack one enzyme (DapC/ArgD), and it is possible that this enzyme could be provided by the host. The bacteriome cells of *B. cockerelli* and *Diaphorina citri* were found to express enhanced levels of several enzymes involved in the biosynthesis of non-essential amino acids tyrosine, cysteine, proline and serine [24]. Also the transcripts of *ribC*, encoding riboflavin synthase, were enriched in the bacteriomes of both of these psyllid species. Thus, it is possible that the bacteriome cells of *T. apicalis* could provide this enzyme for CCr TA and Gpe, which both seem to lack the *ribC* gene. In *D. citri* bacteriome the transcripts related to vitamin B6 and folate were enriched [24], suggesting that the host cells could provide those vitamins for the endosymbionts. As neither CCr nor Gpe of *T. apicalis* have the capacity to produce group B vitamins, these vitamins could be provided either by other bacteria or by the host cells. The partial riboflavin biosynthesis pathway found in Gpe could serve as a part of a shared pathway. Moreover, only a half of the TCA cycle enzymes were identified in the Gpe genome, in accordance with the previous finding of an incomplete TCA cycle in the secondary endosymbionts of *C. eucalypti* and *H. cubana* [43]. The Gpe of *T. apicalis* seems to have lost all the amino acid biosynthesis genes except for those needed in the arginine biosynthesis. These genes may have been retained to compensate the loss of *carA*, *carB* and *argF* from CCr TA. The *argF* gene is present in most of the published genomes of CCr (from *B. trigonica*, *B. cockerelli*, *Cacopsylla* spp., *Pachypsylla* spp., *D. citri* and *Diaphorina* cf. *continua*), but is missing from CCr of *Stenarytaina* spp. [43]. Based on the genomic data, Gpe of *T. apicalis* could provide CCr the proteins required for the bacterial DNA replication, transcription and ribosome biogenesis. Gpe could also provide CCr other enzymes and co-factors required in the bacterial cellular functions. The finding that the relative abundances of these two endosymbiont genomes in *T. apicalis* are close to equal suggests that the two genomes could complement each other almost like two chromosomes of a single organism. Thus, Gpe could be called the co-primary endosymbiont of *T. apicalis*.

## Conclusions

Carrot psyllid *T. apicalis* was found to harbour a previously unknown gamma proteobacterium endosymbiont (Gpe) in addition to the primary endosymbiont ‘*Ca.* C. ruddii’ (CCr) that is indispensable for psyllids. Based on the 16S rRNA gene sequence, the closest relative of Gpe could be the secondary endosymbiont of *T. magnoliae*. The 253.171 kb long genome assembly of Gpe contains the RNA genes and most of the protein-coding genes required for DNA replication, transcription and translation, suggesting it represents the whole genome. Whilst CCr of *T. apicalis* retains a broad amino acid biosynthetic capacity, Gpe seems to lack almost all the amino acid biosynthesis genes. On the other hand, CCr of *T. apicalis* lacks several subunits of DNA polymerase III, has frameshifts in the RNA polymerase genes and lacks many other genes required for transcription and translation, whilst these functions are encoded by the Gpe genome. Altogether, the findings of this study suggest that the reduction of genome size and the complementarity of the biosynthetic capabilities of these two endosymbionts of *T. apicalis* have reached such a level that Gpe, together with CCr, is indispensable for the psyllid host. Further research on *T. apicalis* samples from other geographical regions where this psyllid is found would reveal whether there are some additional, facultative endosymbionts associated with this psyllid species. Genetic studies on the endosymbionts of the other *Trioza* species could shed light on the evolution and possible host exchanges of the secondary endosymbionts within the genus *Trioza*.

## Supplementary Information

Below is the link to the electronic supplementary material.

Supplementary file1 (PDF 181 kb)

Supplementary file2 (PDF 1150 kb)

Supplementary file3 (PDF 1069 kb)

Supplementary file4 (PDF 898 kb)

Supplementary file5 (PDF 2565 kb)

Supplementary file6 (XLSX 16 kb)

Supplementary file7 (XLSX 57 kb)

Supplementary file8 (PDF 169 kb)

## Data Availability

DNA sequence data of the assembled bacterial genomes for ‘*Candidatus* Carsonella ruddii’ and gamma proteobacterium endosymbiont of *T. apicalis* have been deposited to NCBI GenBank under accession numbers CP059138 and CP066222, and the genome assembly of ‘*Candidatus* Liberibacter solanacearum’ FINH40 is under the bioproject PRJNA514334.
