# Supplementary material for: Genomes of the Bacterial Endosymbionts of Carrot Psyllid Trioza apicalis Suggest Complementary Biosynthetic Capabilities
Source: Curr Microbiol. 2025 Feb 20;82(4):145. doi: 10.1007/s00284-025-04119-y (PMC11842425; doi:10.1007/s00284-025-04119-y)
Supplement: Supplementary file 1 — Supplementary file1 (PDF 181 kb) [file 284_2025_4119_MOESM1_ESM.pdf]

Genomes of the bacterial endosymbionts of carrot psyllid *Trioza apicalis* suggest complementary biosynthetic capabilities

Current Microbiology

Sarah Thompson, Jinhui Wang, Thomas Schott, Riitta Nissinen, Minna Haapalainen

University of Helsinki

email: minna.haapalainen@helsinki.fi; minna.haapalainen@luke.fi

Bacterial metapopulation in *T. apicalis*
(Kraken, old sample)

Gammaproteobacteria	368751
Alphaproteobacteria	56940
Betaproteobacteria	26746
Epsilonproteobacteria	4399
Firmicutes	22444
Mollicutes	17414
Unclassified Bacteria	7285
Bacteroidetes	5504
Cyanobacteria	4492
Actinobacteria	1238
Archaea	11953
Other bacteria	51654
	578820

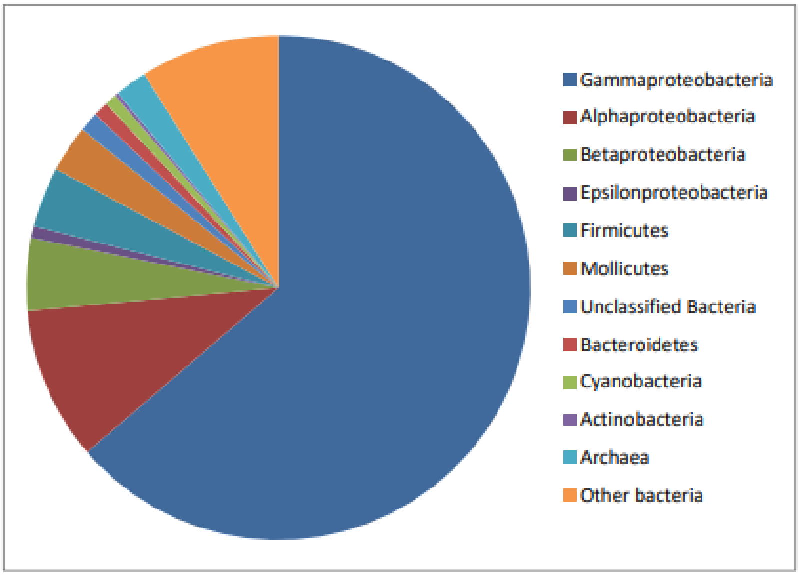

Supplementary Data S1. Different classes of bacteria detected by Kraken analysis in the *Trioza apicalis* metagenome sample 11-H40.
