# Supplementary material for: Genomes of the Bacterial Endosymbionts of Carrot Psyllid Trioza apicalis Suggest Complementary Biosynthetic Capabilities
Source: Curr Microbiol. 2025 Feb 20;82(4):145. doi: 10.1007/s00284-025-04119-y (PMC11842425; doi:10.1007/s00284-025-04119-y)
Supplement: Supplementary file 2 — Supplementary file2 (PDF 1150 kb) [file 284_2025_4119_MOESM2_ESM.pdf]

Genomes of the bacterial endosymbionts of carrot psyllid *Trioza apicalis* suggest complementary biosynthetic capabilities

Current Microbiology

Sarah Thompson, Jinhui Wang, Thomas Schott, Riitta Nissinen, Minna Haapalainen
University of Helsinki, email: minna.haapalainen@helsinki.fi; minna.haapalainen@luke.fi

Supplementary Data S2. Results of Kraken analysis of microbial sequences in the *Trioza apicalis* metagenome sample 11-H40.

% reads covered by clade rooted at this taxon	No. reads covered by clade rooted at this taxon	No. reads assigned directly to this taxon	Rank code	NCBI Tax ID	Scientific Name
99.34	119082603	119082603	U	0	unclassified
0.66	791137	157432	0	1	root
0.50	595136	4363	0	131567	cellular organisms
0.48	578820	43860	D	2	Bacteria
0.40	475860	18512	P	1224	Proteobacteria
0.31	368751	40141	C	1236	Gammaproteobacteria
0.14	167744	2	O	135619	Oceanospirillales
0.14	167741	0	F	28256	Halomonadaceae
0.14	167740	731	0	114403	Zymobacter group
0.14	163282	0	G	114185	Candidatus Carsonella
0.14	163282	54293	S	114186	Candidatus Carsonella ruddii
0.03	35612	0	0	134277	primary endosymbiont of Heteropsylla cubana
0.03	35612	35612	0	1202538	Candidatus Carsonella ruddii HC isolate Thao2000
0.02	29385	29385	0	667013	Candidatus Carsonella ruddii DC
0.01	12768	12768	0	1202540	Candidatus Carsonella ruddii PC isolate NHV
0.01	11876	11876	0	1202539	Candidatus Carsonella ruddii HT isolate Thao2000
0.01	9615	9615	0	1202537	Candidatus Carsonella ruddii CS isolate Thao2000
0.01	6056	6056	0	1202536	Candidatus Carsonella ruddii CE isolate Thao2000
0.00	3677	3677	0	387662	Candidatus Carsonella ruddii PV
0.00	3727	0	0	114399	whitefly endosymbionts
0.00	3727	0	G	235572	Candidatus Portiera
0.00	3727	2014	S	91844	Candidatus Portiera aleyrodidarum
0.00	1663	1663	0	1297582	Candidatus Portiera aleyrodidarum TV
0.00	49	49	0	1206109	Candidatus Portiera aleyrodidarum BT-B-HRs
0.00	1	1	0	1239881	Candidatus Portiera aleyrodidarum BT-QVLC
0.00	1	0	G	42054	Chromohalobacter
0.00	1	0	S	158080	Chromohalobacter salexigens
0.00	1	1	0	290398	Chromohalobacter salexigens DSM 3043
0.00	1	0	F	135620	Oceanospirillaceae
0.00	1	0	G	28253	Marinomonas
0.00	1	0	S	119864	Marinomonas mediterranea
0.00	1	1	0	717774	Marinomonas mediterranea MMB-1
0.11	128921	0	O	91347	Enterobacteriales
0.11	128921	23443	F	543	Enterobacteriaceae
0.04	49148	0	G	32199	Buchnera
0.04	49148	8332	S	9	Buchnera aphidicola
0.01	13288	13288	0	118099	Buchnera aphidicola (Acyrthosiphon pisum)
0.01	10028	0	0	118117	Buchnera aphidicola (Uroleucon ambrosiae)
0.01	10028	10028	0	1005057	Buchnera aphidicola str. Ua (Uroleucon ambrosiae)
0.01	7978	0	0	135842	Buchnera aphidicola (Baizongia pistaciae)
0.01	7978	7978	0	224915	Buchnera aphidicola str. Bp (Baizongia pistaciae)
0.00	5039	0	0	668607	Buchnera aphidicola (Acyrthosiphon kondoi)
0.00	5039	5039	0	1005090	Buchnera aphidicola str. Ak (Acyrthosiphon kondoi)
0.00	3627	0	0	98794	Buchnera aphidicola (Schizaphis graminum)
0.00	3627	3627	0	198804	Buchnera aphidicola str. Sg (Schizaphis graminum)
0.00	854	0	0	261318	Buchnera aphidicola (Cinara cedri)
0.00	854	854	0	372461	Buchnera aphidicola BCc
0.00	2	2	0	261317	Buchnera aphidicola (Cinara tujafilina)
0.03	31527	0	0	191675	unclassified Enterobacteriaceae
0.03	31525	1	0	84563	ant, tsetse, mealybug, aphid, etc. endosymbionts
0.02	25513	0	0	84564	ant endosymbionts
0.02	25513	4075	G	203804	Candidatus Blochmannia
0.01	11259	11259	S	203907	Candidatus Blochmannia floridanus
0.01	10177	0	S	251535	Candidatus Blochmannia vafer
0.01	10177	10177	0	859654	Candidatus Blochmannia vafer str. BVAF
0.00	1	0	S	101534	Candidatus Blochmannia pennsylvanicus
0.00	1	1	0	291272	Candidatus Blochmannia pennsylvanicus str. BPEN
0.00	1	0	S	251542	Candidatus Blochmannia chromaiodes
0.00	1	1	0	1240471	Candidatus Blochmannia chromaiodes str. 640
0.01	6011	0	0	146507	aphid secondary symbionts
0.01	6009	6009	S	134287	secondary endosymbiont of Heteropsylla cubana
0.00	1	0	G	568987	Candidatus Hamiltonella
0.00	1	0	S	138072	Candidatus Hamiltonella defensa
0.00	1	1	0	572265	Candidatus Hamiltonella defensa 5AT (Acyrthosiphon pisum)
0.00	1	1	S	1199245	secondary endosymbiont of Ctenarytaina eucalypti
0.00	2	0	0	36866	unclassified Enterobacteriaceae (miscellaneous)
0.00	2	2	S	693444	Enterobacteriaceae bacterium strain FGI 57
0.01	9675	0	G	51228	Wigglesworthia
0.01	9675	0	S	51229	Wigglesworthia glossinidia
0.01	8141	8141	0	36870	Wigglesworthia glossinidia endosymbiont of Glossina brevipalpis

0.00	1534	0	0	36868	Wigglesworthia glossinidia endosymbiont of Glossina morsitans
0.00	1534	1534	0	1142511	Wigglesworthia glossinidia endosymbiont of Glossina morsitans morsitans (Yale colony)
0.00	5013	0	G	401618	Candidatus Riesia
0.00	5013	0	S	401619	Candidatus Riesia pediculicola
0.00	5013	5013	0	515618	Candidatus Riesia pediculicola USDA
0.00	4987	0	G	613	Serratia
0.00	4972	0	S	138074	Serratia symbiotica
0.00	4972	4972	0	568817	Serratia symbiotica str. 'Cinara cedri'
0.00	14	0	S	614	Serratia liquefaciens
0.00	14	14	0	1346614	Serratia liquefaciens ATCC 27592
0.00	1	0	S	615	Serratia marcescens
0.00	1	1	0	1249634	Serratia marcescens FGI94
0.00	4425	0	G	1048757	Candidatus Moranella
0.00	4425	4425	S	1048758	Candidatus Moranella endobia
0.00	320	0	G	1335483	Shimwellia
0.00	320	0	S	563	Shimwellia blattae
0.00	320	320	0	630626	Shimwellia blattae DSM 4481 = NBRC 105725
0.00	98	0	G	561	Escherichia
0.00	98	20	S	562	Escherichia coli
0.00	70	0	0	83334	Escherichia coli O157:H7
0.00	70	70	0	444450	Escherichia coli O157:H7 str. EC4115
0.00	7	7	0	405955	Escherichia coli APEC O1
0.00	1	1	0	655817	Escherichia coli ABU 83972
0.00	61	0	G	629	Yersinia
0.00	54	0	S	630	Yersinia enterocolitica
0.00	54	0	0	150053	Yersinia enterocolitica subsp. palearctica
0.00	27	27	0	930944	Yersinia enterocolitica subsp. palearctica Y11
0.00	27	27	0	994476	Yersinia enterocolitica subsp. palearctica 105.5R(r)
0.00	7	0	S	633	Yersinia pseudotuberculosis
0.00	7	7	0	502800	Yersinia pseudotuberculosis YPIII
0.00	59	0	G	547	Enterobacter
0.00	32	1	S	548	Enterobacter aerogenes
0.00	31	31	0	935296	Enterobacter aerogenes EA1509E
0.00	26	0	0	354276	Enterobacter cloacae complex
0.00	20	1	S	550	Enterobacter cloacae
0.00	11	11	0	1045856	Enterobacter cloacae EcWSU1
0.00	8	0	0	336306	Enterobacter cloacae subsp. cloacae
0.00	4	4	0	718254	Enterobacter cloacae subsp. cloacae NCTC 9394
0.00	4	4	0	1211025	Enterobacter cloacae subsp. cloacae ENHKU01
0.00	5	0	S	1334193	Enterobacter lignolyticus
0.00	5	5	0	701347	Enterobacter lignolyticus SCF1
0.00	1	0	S	61645	Enterobacter asburiae
0.00	1	1	0	640513	Enterobacter asburiae LF7a
0.00	1	1	S	1166130	Enterobacter sp. R4-368
0.00	50	0	G	84565	Sodalis
0.00	50	0	S	63612	Sodalis glossinidius
0.00	50	50	0	343509	Sodalis glossinidius str. 'morsitans'
0.00	46	0	G	29487	Photorhabdus
0.00	45	0	S	29488	Photorhabdus luminescens
0.00	45	0	0	141679	Photorhabdus luminescens subsp. laumondii
0.00	45	45	0	243265	Photorhabdus luminescens subsp. laumondii TTO1
0.00	1	1	S	291112	Photorhabdus asymbiotica
0.00	30	0	G	583	Proteus
0.00	30	0	S	584	Proteus mirabilis
0.00	30	30	0	1266738	Proteus mirabilis BB2000
0.00	16	6	G	570	Klebsiella
0.00	7	6	S	573	Klebsiella pneumoniae
0.00	1	1	0	1244085	Klebsiella pneumoniae CG43
0.00	2	0	S	244366	Klebsiella variicola
0.00	2	2	0	640131	Klebsiella variicola At-22
0.00	1	1	S	571	Klebsiella oxytoca
0.00	9	0	G	620	Shigella
0.00	9	9	S	624	Shigella sonnei
0.00	3	0	G	626	Xenorhabdus
0.00	3	0	S	628	Xenorhabdus nematophila
0.00	3	3	0	406817	Xenorhabdus nematophila ATCC 19061
0.00	2	0	G	590	Salmonella
0.00	1	0	S	28901	Salmonella enterica
0.00	1	0	0	59201	Salmonella enterica subsp. enterica
0.00	1	0	0	600	Salmonella enterica subsp. enterica serovar Thompson
0.00	1	1	0	1064551	Salmonella enterica subsp. enterica serovar Thompson str. RM6836
0.00	1	0	S	54736	Salmonella bongori
0.00	1	1	0	1197719	Salmonella bongori N268-08
0.00	2	0	G	53335	Pantoea
0.00	2	2	S	553	Pantoea ananatis
0.00	2	0	G	122277	Pectobacterium
0.00	1	0	S	554	Pectobacterium carotovorum
0.00	1	0	0	555	Pectobacterium carotovorum subsp. carotovorum
0.00	1	1	0	1218933	Pectobacterium carotovorum subsp. carotovorum PCC21
0.00	1	1	S	1166016	Pectobacterium sp. SCC3193
0.00	1	0	G	544	Citrobacter
0.00	1	0	S	545	Citrobacter koseri
0.00	1	1	0	290338	Citrobacter koseri ATCC BAA-895

0.00	1	0	G	551	Erwinia
0.00	1	1	S	552	Erwinia amylovora
0.00	1	1	G	34037	Rahnella
0.00	1	0	G	160674	Raoultella
0.00	1	0	S	54291	Raoultella ornithinolytica
0.00	1	1	0	1286170	Raoultella ornithinolytica B6
0.00	1	0	G	413496	Cronobacter
0.00	1	0	S	413502	Cronobacter turicensis
0.00	1	1	0	693216	Cronobacter turicensis z3032
0.01	13978	0	O	72274	Pseudomonadales
0.01	10935	1	F	468	Moraxellaceae
0.00	5593	4209	G	469	Acinetobacter
0.00	1381	0	0	909768	Acinetobacter calcoaceticus/baumannii complex
0.00	1380	19	S	470	Acinetobacter baumannii
0.00	1336	1336	0	1400867	Acinetobacter baumannii ZW85-1
0.00	22	22	0	497978	Acinetobacter baumannii MDR-ZJ06
0.00	2	2	0	509170	Acinetobacter baumannii SDF
0.00	1	1	0	405416	Acinetobacter baumannii ACICU
0.00	1	0	S	471	Acinetobacter calcoaceticus
0.00	1	1	0	871585	Acinetobacter calcoaceticus PHEA-2
0.00	2	0	S	1148157	Acinetobacter oleivorans
0.00	2	2	0	436717	Acinetobacter oleivorans DR1
0.00	1	1	S	62977	Acinetobacter sp. ADP1
0.00	5336	0	G	475	Moraxella
0.00	5336	0	0	46226	Branhamella
0.00	5336	0	S	480	Moraxella catarrhalis
0.00	5336	5336	0	749219	Moraxella catarrhalis RH4
0.00	5	2	G	497	Psychrobacter
0.00	3	0	S	330922	Psychrobacter cryohalolentis
0.00	3	3	0	335284	Psychrobacter cryohalolentis K5
0.00	3043	16	F	135621	Pseudomonadaceae
0.00	3025	911	G	286	Pseudomonas
0.00	1134	165	0	136843	Pseudomonas fluorescens group
0.00	686	95	S	294	Pseudomonas fluorescens
0.00	279	279	0	216595	Pseudomonas fluorescens SBW25
0.00	201	201	0	1037911	Pseudomonas fluorescens A506
0.00	79	79	0	205922	Pseudomonas fluorescens Pf0-1
0.00	32	32	0	1114970	Pseudomonas fluorescens F113
0.00	159	0	S	200451	Pseudomonas poae
0.00	159	159	0	1282356	Pseudomonas poae RE*1-1-14
0.00	124	107	S	380021	Pseudomonas protegens
0.00	11	11	0	220664	Pseudomonas protegens Pf-5
0.00	6	6	0	1124983	Pseudomonas protegens CHA0
0.00	313	15	0	136845	Pseudomonas putida group
0.00	287	29	S	303	Pseudomonas putida
0.00	219	219	0	231023	Pseudomonas putida ND6
0.00	10	10	0	76869	Pseudomonas putida GB-1
0.00	9	9	0	1215088	Pseudomonas putida HB3267
0.00	8	8	0	390235	Pseudomonas putida W619
0.00	4	4	0	1211579	Pseudomonas putida NBRC 14164
0.00	4	4	0	1331671	Pseudomonas putida H8234
0.00	2	2	0	160488	Pseudomonas putida KT2440
0.00	1	1	0	931281	Pseudomonas putida BIRD-1
0.00	1	1	0	1042876	Pseudomonas putida S16
0.00	9	0	S	47880	Pseudomonas fulva
0.00	9	9	0	743720	Pseudomonas fulva 12-X
0.00	2	2	S	76759	Pseudomonas monteilii
0.00	289	289	S	1415630	Pseudomonas sp. TKP
0.00	114	10	0	136849	Pseudomonas syringae group
0.00	70	0	0	251698	Pseudomonas syringae group genomosp. 2
0.00	70	0	S	29438	Pseudomonas savastanoi
0.00	70	0	0	319	Pseudomonas syringae pv. phaseolicola
0.00	70	70	0	264730	Pseudomonas syringae pv. phaseolicola 1448A
0.00	20	0	S	251701	Pseudomonas syringae group genomosp. 3
0.00	20	0	0	323	Pseudomonas syringae pv. tomato
0.00	20	20	0	223283	Pseudomonas syringae pv. tomato str. DC3000
0.00	14	0	0	251695	Pseudomonas syringae group genomosp. 1
0.00	14	0	S	317	Pseudomonas syringae
0.00	14	0	0	321	Pseudomonas syringae pv. syringae
0.00	14	14	0	205918	Pseudomonas syringae pv. syringae B728a
0.00	72	72	S	1207075	Pseudomonas sp. UW4
0.00	71	71	S	69328	Pseudomonas sp. VLB120
0.00	59	2	0	136841	Pseudomonas aeruginosa group
0.00	34	20	S	287	Pseudomonas aeruginosa
0.00	8	8	0	1352355	Pseudomonas aeruginosa c7447m
0.00	3	3	0	381754	Pseudomonas aeruginosa PA7
0.00	2	2	0	208963	Pseudomonas aeruginosa UCBPP-PA14
0.00	1	1	0	1427342	Pseudomonas aeruginosa SCV20265
0.00	15	4	S	300	Pseudomonas mendocina
0.00	8	8	0	1001585	Pseudomonas mendocina NK-01
0.00	3	3	0	399739	Pseudomonas mendocina ymp
0.00	8	0	S	53412	Pseudomonas resinovorans
0.00	8	8	0	1245471	Pseudomonas resinovorans NBRC 106553

0.00	19	0	0	136846	Pseudomonas stutzeri group
0.00	19	0	0	578833	Pseudomonas stutzeri subgroup
0.00	19	10	S	316	Pseudomonas stutzeri
0.00	3	3	0	379731	Pseudomonas stutzeri A1501
0.00	2	2	0	1123519	Pseudomonas stutzeri DSM 10701
0.00	2	2	0	1196835	Pseudomonas stutzeri CCUG 29243
0.00	1	1	0	644801	Pseudomonas stutzeri RCH2
0.00	1	1	0	996285	Pseudomonas stutzeri DSM 4166
0.00	19	0	S	930166	Pseudomonas brassicacearum
0.00	19	0	0	86264	Pseudomonas brassicacearum subsp. brassicacearum
0.00	19	19	0	994484	Pseudomonas brassicacearum subsp. brassicacearum NFM421
0.00	16	0	S	312306	Pseudomonas entomophila
0.00	16	16	0	384676	Pseudomonas entomophila L48
0.00	8	0	0	136844	Pseudomonas pertucinogena group
0.00	8	0	S	43306	Pseudomonas denitrificans
0.00	8	8	0	1294143	Pseudomonas denitrificans ATCC 13867
0.00	2	0	0	351	Azotobacter group
0.00	2	0	G	352	Azotobacter
0.00	2	2	S	354	Azotobacter vinelandii
0.01	13704	0	0	118884	unclassified Gammaproteobacteria
0.01	13702	0	0	198346	Candidatus Baumannia
0.01	13702	0	S	186490	Candidatus Baumannia cicadellinicola
0.01	13702	13702	0	374463	Baumannia cicadellinicola str. Hc (Homalodisca coagulata)
0.00	2	1	0	32036	sulfur-oxidizing symbionts
0.00	1	0	S	410330	Calyptogena okutanii thioautotrophic gill symbiont
0.00	1	1	0	412965	Candidatus Vesicomyosocius okutanii HA
0.00	2152	0	O	135622	Alteromonadales
0.00	1328	0	F	267890	Shewanellaceae
0.00	1328	1	G	22	Shewanella
0.00	1284	0	S	60217	Shewanella violacea
0.00	1284	1284	0	637905	Shewanella violacea DSS12
0.00	41	0	S	62322	Shewanella baltica
0.00	41	41	0	693970	Shewanella baltica OS117
0.00	1	0	S	60478	Shewanella amazonensis
0.00	1	1	0	326297	Shewanella amazonensis SB2B
0.00	1	0	S	271098	Shewanella halifaxensis
0.00	1	1	0	458817	Shewanella halifaxensis HAW-EB4
0.00	756	0	F	267889	Colwelliaceae
0.00	756	0	G	28228	Colwellia
0.00	756	0	S	28229	Colwellia psychrerythraea
0.00	756	756	0	167879	Colwellia psychrerythraea 34H
0.00	65	0	F	72275	Alteromonadaceae
0.00	26	0	G	89404	Glaciecola
0.00	26	0	S	326544	Glaciecola psychrophila
0.00	26	26	0	1129794	Glaciecola psychrophila 170
0.00	22	0	G	226	Alteromonas
0.00	22	0	S	28108	Alteromonas macleodii
0.00	22	22	0	1300257	Alteromonas macleodii str. 'Ionian Sea U8'
0.00	17	0	G	316625	Saccharophagus
0.00	17	0	S	86304	Saccharophagus degradans
0.00	17	17	0	203122	Saccharophagus degradans 2-40
0.00	3	0	F	267894	Psychromonadaceae
0.00	3	0	G	67572	Psychromonas
0.00	2	2	S	314282	Psychromonas sp. CNPT3
0.00	1	0	S	357794	Psychromonas ingrahamii
0.00	1	1	0	357804	Psychromonas ingrahamii 37
0.00	886	0	O	135614	Xanthomonadales
0.00	886	73	F	32033	Xanthomonadaceae
0.00	758	0	G	338	Xanthomonas
0.00	755	162	S	339	Xanthomonas campestris
0.00	481	4	0	340	Xanthomonas campestris pv. campestris
0.00	476	476	0	314565	Xanthomonas campestris pv. campestris str. 8004
0.00	1	1	0	509169	Xanthomonas campestris pv. campestris str. B100
0.00	112	0	0	359385	Xanthomonas campestris pv. raphani
0.00	112	112	0	990315	Xanthomonas campestris pv. raphani 756C
0.00	2	1	S	347	Xanthomonas oryzae
0.00	1	1	0	64187	Xanthomonas oryzae pv. oryzae
0.00	1	0	0	643453	Xanthomonas citri group
0.00	1	0	S	366648	Xanthomonas fuscans
0.00	1	1	0	366649	Xanthomonas fuscans subsp. fuscans
0.00	27	1	G	83618	Pseudoxanthomonas
0.00	26	0	S	415229	Pseudoxanthomonas spadix
0.00	26	26	0	1045855	Pseudoxanthomonas spadix BD-a59
0.00	26	0	G	40323	Stenotrophomonas
0.00	26	0	0	995085	Stenotrophomonas maltophilia group
0.00	26	11	S	40324	Stenotrophomonas maltophilia
0.00	7	7	0	391008	Stenotrophomonas maltophilia R551-3
0.00	4	4	0	522373	Stenotrophomonas maltophilia K279a
0.00	3	3	0	868597	Stenotrophomonas maltophilia JV3
0.00	1	1	0	1163399	Stenotrophomonas maltophilia D457
0.00	1	0	G	2370	Xylella
0.00	1	1	S	2371	Xylella fastidiosa
0.00	1	0	G	75309	Rhodanobacter

0.00	1	1	S	666685	Rhodanobacter denitrificans
0.00	833	0	O	135613	Chromatiales
0.00	831	0	F	72276	Ectothiorhodospiraceae
0.00	830	0	G	133193	Alkalilimnicola
0.00	830	0	S	351052	Alkalilimnicola ehrlichii
0.00	830	830	0	187272	Alkalilimnicola ehrlichii MLHE-1
0.00	1	0	G	85108	Halorhodospira
0.00	1	0	S	1053	Halorhodospira halophila
0.00	1	1	0	349124	Halorhodospira halophila SL1
0.00	2	0	F	1046	Chromatiaceae
0.00	1	0	G	1227	Nitrosococcus
0.00	1	0	S	473531	Nitrosococcus watsonii
0.00	1	1	0	105559	Nitrosococcus watsonii C-113
0.00	1	0	G	13724	Thiocystis
0.00	1	0	S	73141	Thiocystis violascens
0.00	1	1	0	765911	Thiocystis violascens DSM 198
0.00	254	0	O	135625	Pasteurellales
0.00	254	1	F	712	Pasteurellaceae
0.00	98	1	G	724	Haemophilus
0.00	90	80	S	727	Haemophilus influenzae
0.00	8	8	0	71421	Haemophilus influenzae Rd KW20
0.00	1	1	0	374931	Haemophilus influenzae PittGG
0.00	1	1	0	1334187	Haemophilus influenzae KR494
0.00	6	0	S	729	Haemophilus parainfluenzae
0.00	6	6	0	862965	Haemophilus parainfluenzae T3T1
0.00	1	0	S	730	Haemophilus ducreyi
0.00	1	1	0	233412	Haemophilus ducreyi 35000HP
0.00	83	0	G	214906	Histophilus
0.00	83	1	S	731	Histophilus somni
0.00	70	70	0	205914	Haemophilus somnus 129PT
0.00	12	12	0	228400	Haemophilus somnus 2336
0.00	68	0	G	75984	Mannheimia
0.00	68	67	S	75985	Mannheimia haemolytica
0.00	1	1	0	1311759	Mannheimia haemolytica D171
0.00	2	0	G	745	Pasteurella
0.00	2	1	S	747	Pasteurella multocida
0.00	1	0	0	44283	Pasteurella multocida subsp. multocida
0.00	1	1	0	272843	Pasteurella multocida subsp. multocida str. Pm70
0.00	2	0	G	416916	Aggregatibacter
0.00	1	0	S	714	Aggregatibacter actinomycetemcomitans
0.00	1	1	0	754507	Aggregatibacter actinomycetemcomitans ANH9381
0.00	1	0	S	732	Aggregatibacter aphrophilus
0.00	1	1	0	634176	Aggregatibacter aphrophilus NJ8700
0.00	84	0	O	135623	Vibrionales
0.00	84	0	F	641	Vibrionaceae
0.00	44	0	G	662	Vibrio
0.00	44	43	S	666	Vibrio cholerae
0.00	1	1	0	935297	Vibrio cholerae LMA3984-4
0.00	40	0	G	511678	Aliivibrio
0.00	40	0	S	40269	Aliivibrio salmonicida
0.00	40	40	0	316275	Aliivibrio salmonicida LFI1238
0.00	42	0	O	118969	Legionellales
0.00	42	0	F	444	Legionellaceae
0.00	42	0	G	445	Legionella
0.00	42	0	S	450	Legionella longbeachae
0.00	42	42	0	661367	Legionella longbeachae NSW150
0.00	9	0	O	72273	Thiotrichales
0.00	7	0	F	135616	Piscirickettsiaceae
0.00	6	6	G	34067	Cycloclasticus
0.00	1	0	G	92244	Thioalkalimicrobium
0.00	1	0	S	147268	Thioalkalimicrobium cyclicum
0.00	1	1	0	717773	Thioalkalimicrobium cyclicum ALM1
0.00	2	0	F	34064	Francisellaceae
0.00	2	1	G	262	Francisella
0.00	1	0	S	657445	Francisella noatunensis
0.00	1	1	0	299583	Francisella noatunensis subsp. orientalis
0.00	2	0	O	135624	Aeromonadales
0.00	2	1	F	84642	Aeromonadaceae
0.00	1	0	G	642	Aeromonas
0.00	1	0	S	654	Aeromonas veronii
0.00	1	1	0	998088	Aeromonas veronii B565
0.00	1	0	O	135618	Methylococcales
0.00	1	0	F	403	Methylococcaceae
0.00	1	0	G	39773	Methylomicrobium
0.00	1	0	S	271065	Methylomicrobium alcaliphilum
0.00	1	1	0	1091494	Methylomicrobium alcaliphilum 20Z
0.05	56940	41	C	28211	Alphaproteobacteria
0.04	52922	65	O	356	Rhizobiales
0.04	51781	8	F	82115	Rhizobiaceae
0.04	51619	220	G	34019	Candidatus Liberibacter
0.04	51360	0	S	556287	Candidatus Liberibacter solanacearum
0.04	51360	51360	0	658172	Candidatus Liberibacter solanacearum CLso-ZC1
0.00	26	16	S	34021	Candidatus Liberibacter asiaticus

0.00	10	10	0	1174529	Candidatus Liberibacter asiaticus str. gxpsy
0.00	12	0	S	309868	Candidatus Liberibacter americanus
0.00	12	12	0	1261131	Candidatus Liberibacter americanus str. Sao Paulo
0.00	1	0	S	1273132	Liberibacter crescens
0.00	1	1	0	1215343	Liberibacter crescens BT-1
0.00	134	3	0	227290	Rhizobium/Agrobacterium group
0.00	120	6	G	379	Rhizobium
0.00	104	3	S	384	Rhizobium leguminosarum
0.00	95	0	0	387	Rhizobium leguminosarum bv. viciae
0.00	95	95	0	216596	Rhizobium leguminosarum bv. viciae 3841
0.00	6	0	0	386	Rhizobium leguminosarum bv. trifolii
0.00	3	3	0	395491	Rhizobium leguminosarum bv. trifolii WSM1325
0.00	3	3	0	395492	Rhizobium leguminosarum bv. trifolii WSM2304
0.00	4	4	S	424182	Rhizobium sp. IRBG74
0.00	3	0	S	398	Rhizobium tropici
0.00	3	3	0	698761	Rhizobium tropici CIAT 899
0.00	3	1	S	29449	Rhizobium etli
0.00	1	0	0	323733	Rhizobium etli bv. mimosae
0.00	1	1	0	1328306	Rhizobium etli bv. mimosae str. Mim1
0.00	1	1	0	491916	Rhizobium etli CIAT 652
0.00	11	0	G	357	Agrobacterium
0.00	7	0	0	1183400	Agrobacterium tumefaciens complex
0.00	5	0	S	358	Agrobacterium tumefaciens
0.00	5	5	0	311403	Agrobacterium radiobacter K84
0.00	2	0	S	1176649	Agrobacterium fabrum
0.00	2	2	0	176299	Agrobacterium fabrum str. C58
0.00	4	4	S	861208	Agrobacterium sp. H13-3
0.00	20	0	0	227292	Sinorhizobium/Ensifer group
0.00	20	2	G	28105	Sinorhizobium
0.00	11	0	0	663276	Sinorhizobium fredii group
0.00	11	0	S	380	Sinorhizobium fredii
0.00	10	10	0	1185652	Sinorhizobium fredii USDA 257
0.00	1	1	0	1117943	Sinorhizobium fredii HH103
0.00	6	4	S	382	Sinorhizobium meliloti
0.00	1	1	0	1230587	Sinorhizobium meliloti Rm41
0.00	1	1	0	1235461	Sinorhizobium meliloti GR4
0.00	1	0	S	110321	Sinorhizobium medicae
0.00	1	1	0	366394	Sinorhizobium medicae WSM419
0.00	856	22	F	41294	Bradyrhizobiaceae
0.00	757	41	G	374	Bradyrhizobium
0.00	669	669	S	288000	Bradyrhizobium sp. BTAi1
0.00	14	14	S	114615	Bradyrhizobium sp. ORS 278
0.00	10	0	S	1355477	Bradyrhizobium diazoefficiens
0.00	10	10	0	224911	Bradyrhizobium diazoefficiens USDA 110
0.00	9	0	S	44255	Bradyrhizobium oligotrophicum
0.00	9	9	0	1245469	Bradyrhizobium oligotrophicum S58
0.00	8	8	S	335659	Bradyrhizobium sp. S23321
0.00	6	0	S	375	Bradyrhizobium japonicum
0.00	6	6	0	1037409	Bradyrhizobium japonicum USDA 6
0.00	61	0	G	1073	Rhodopseudomonas
0.00	61	37	S	1076	Rhodopseudomonas palustris
0.00	8	8	0	316058	Rhodopseudomonas palustris HaA2
0.00	6	6	0	316057	Rhodopseudomonas palustris BisB5
0.00	5	5	0	316056	Rhodopseudomonas palustris BisB18
0.00	3	3	0	316055	Rhodopseudomonas palustris BisA53
0.00	1	1	0	258594	Rhodopseudomonas palustris CGA009
0.00	1	1	0	395960	Rhodopseudomonas palustris TIE-1
0.00	12	3	G	911	Nitrobacter
0.00	9	0	S	912	Nitrobacter hamburgensis
0.00	9	9	0	323097	Nitrobacter hamburgensis X14
0.00	4	0	G	40136	Oligotropha
0.00	4	4	S	40137	Oligotropha carboxidovorans
0.00	59	0	F	45401	Hyphomicrobiaceae
0.00	48	0	G	1082930	Pelagibacterium
0.00	48	0	S	531813	Pelagibacterium halotolerans
0.00	48	48	0	1082931	Pelagibacterium halotolerans B2
0.00	10	0	G	1068	Rhodomicrobium
0.00	10	0	S	1069	Rhodomicrobium vannielii
0.00	10	10	0	648757	Rhodomicrobium vannielii ATCC 17100
0.00	1	0	G	81	Hyphomicrobium
0.00	1	0	S	1427356	Hyphomicrobium nitrativorans
0.00	1	1	0	1029756	Hyphomicrobium nitrativorans NL23
0.00	53	0	0	119042	unclassified Rhizobiales
0.00	53	0	G	573657	Candidatus Hodgkinia
0.00	53	0	S	573658	Candidatus Hodgkinia cicadicola
0.00	53	53	0	573234	Candidatus Hodgkinia cicadicola Dsem
0.00	45	0	F	119045	Methylobacteriaceae
0.00	45	10	G	407	Methylobacterium
0.00	10	0	0	578822	Methylobacterium extorquens group
0.00	10	4	S	408	Methylobacterium extorquens
0.00	4	4	0	440085	Methylobacterium extorquens CM4
0.00	2	2	0	661410	Methylobacterium extorquens DM4
0.00	9	0	S	114616	Methylobacterium nodulans

0.00	9	9	0	460265	Methylobacterium nodulans ORS 2060
0.00	8	0	S	31998	Methylobacterium radiotolerans
0.00	8	8	0	426355	Methylobacterium radiotolerans JCM 2831
0.00	5	5	S	426117	Methylobacterium sp. 4-46
0.00	3	0	S	223967	Methylobacterium populi
0.00	3	3	0	441620	Methylobacterium populi BJ001
0.00	20	1	F	335928	Xanthobacteraceae
0.00	10	0	G	279	Xanthobacter
0.00	10	0	S	280	Xanthobacter autotrophicus
0.00	10	10	0	78245	Xanthobacter autotrophicus Py2
0.00	6	0	G	152053	Starkeya
0.00	6	0	S	921	Starkeya novella
0.00	6	6	0	639283	Starkeya novella DSM 506
0.00	3	0	G	6	Azorhizobium
0.00	3	0	S	7	Azorhizobium caulinodans
0.00	3	3	0	438753	Azorhizobium caulinodans ORS 571
0.00	18	0	F	69277	Phyllobacteriaceae
0.00	12	4	G	68287	Mesorhizobium
0.00	4	0	S	381	Mesorhizobium loti
0.00	4	4	0	266835	Mesorhizobium loti MAFF303099
0.00	3	0	S	39645	Mesorhizobium ciceri
0.00	3	0	0	278148	Mesorhizobium ciceri biovar biserrulae
0.00	3	3	0	765698	Mesorhizobium ciceri biovar biserrulae WSM1271
0.00	1	0	S	593909	Mesorhizobium opportunistum
0.00	1	1	0	536019	Mesorhizobium opportunistum WSM2075
0.00	6	0	G	449972	Chelativorans
0.00	6	6	S	266779	Chelativorans sp. BNC1
0.00	11	0	F	772	Bartonellaceae
0.00	11	0	G	773	Bartonella
0.00	11	0	S	803	Bartonella quintana
0.00	11	11	0	283165	Bartonella quintana str. Toulouse
0.00	5	0	F	118882	Brucellaceae
0.00	3	0	G	528	Ochrobactrum
0.00	3	0	S	529	Ochrobactrum anthropi
0.00	3	3	0	439375	Ochrobactrum anthropi ATCC 49188
0.00	2	2	G	234	Brucella
0.00	4	0	F	31993	Methylocystaceae
0.00	4	0	G	133	Methylocystis
0.00	4	4	S	187303	Methylocystis sp. SC2
0.00	4	0	F	45404	Beijerinckiaceae
0.00	3	0	G	120652	Methylocella
0.00	3	0	S	199596	Methylocella silvestris
0.00	3	3	0	395965	Methylocella silvestris BL2
0.00	1	0	G	532	Beijerinckia
0.00	1	0	S	533	Beijerinckia indica
0.00	1	0	0	31994	Beijerinckia indica subsp. indica
0.00	1	1	0	395963	Beijerinckia indica subsp. indica ATCC 9039
0.00	1	0	F	119043	Rhodobiaceae
0.00	1	0	G	256616	Parvibaculum
0.00	1	0	S	256618	Parvibaculum lavamentivorans
0.00	1	1	0	402881	Parvibaculum lavamentivorans DS-1
0.00	2538	0	O	766	Rickettsiales
0.00	2244	0	F	942	Anaplasmataceae
0.00	1309	0	G	768	Anaplasma
0.00	1309	0	S	769	Anaplasma centrale
0.00	1309	1309	0	574556	Anaplasma centrale str. Israel
0.00	846	0	0	952	Wolbachieae
0.00	846	214	G	953	Wolbachia
0.00	367	0	S	263437	Wolbachia endosymbiont of Culex quinquefasciatus
0.00	367	367	0	570417	Wolbachia endosymbiont of Culex quinquefasciatus Pel
0.00	219	0	S	77038	Wolbachia endosymbiont of Drosophila simulans
0.00	219	219	0	1236908	Wolbachia endosymbiont of Drosophila simulans wNo
0.00	44	44	S	66084	Wolbachia sp. wRi
0.00	2	2	S	100901	Wolbachia endosymbiont of Onchocerca ochengi
0.00	89	0	G	943	Ehrlichia
0.00	89	4	0	106178	canis group
0.00	76	0	S	779	Ehrlichia ruminantium
0.00	76	76	0	254945	Ehrlichia ruminantium str. Welgevonden
0.00	9	0	S	944	Ehrlichia canis
0.00	9	9	0	269484	Ehrlichia canis str. Jake
0.00	263	0	F	775	Rickettsiaceae
0.00	263	0	0	33988	Rickettsieae
0.00	263	27	G	780	Rickettsia
0.00	224	168	0	114277	spotted fever group
0.00	49	0	S	35788	Rickettsia africae
0.00	49	49	0	347255	Rickettsia africae ESF-5
0.00	5	0	S	33989	Candidatus Rickettsia amblyommii
0.00	5	5	0	1105111	Candidatus Rickettsia amblyommii str. GAT-30V
0.00	2	0	S	33992	Rickettsia rhipicephali
0.00	2	2	0	1105113	Rickettsia rhipicephali str. 3-7-female6-CWPP
0.00	12	0	0	1129742	belli group
0.00	7	7	S	788	Rickettsia canadensis
0.00	5	5	S	33990	Rickettsia bellii

0.00	31	0	F	1328881	Candidatus Midichloriaceae
0.00	31	0	G	411566	Candidatus Midichloria
0.00	31	0	S	234827	Candidatus Midichloria mitochondrii
0.00	31	31	0	696127	Candidatus Midichloria mitochondrii IricVA
0.00	1336	0	0	82117	unclassified Alphaproteobacteria
0.00	989	0	G	213485	Micavibrio
0.00	989	989	S	349221	Micavibrio aeruginosavorus
0.00	344	1	0	54526	SAR11 cluster
0.00	343	0	G	198251	Candidatus Pelagibacter
0.00	343	0	S	198252	Candidatus Pelagibacter ubique
0.00	343	343	0	335992	Candidatus Pelagibacter ubique HTCC1062
0.00	3	0	0	991903	Polymorphum
0.00	3	0	S	991904	Polymorphum gilvum
0.00	3	3	0	991905	Polymorphum gilvum SL003B-26A1
0.00	39	0	O	204455	Rhodobacterales
0.00	37	2	F	31989	Rhodobacteraceae
0.00	26	0	G	1060	Rhodobacter
0.00	22	21	S	1063	Rhodobacter sphaeroides
0.00	1	1	0	349102	Rhodobacter sphaeroides ATCC 17025
0.00	4	0	S	1061	Rhodobacter capsulatus
0.00	4	4	0	272942	Rhodobacter capsulatus SB 1003
0.00	5	0	G	265	Paracoccus
0.00	5	0	S	266	Paracoccus denitrificans
0.00	5	5	0	318586	Paracoccus denitrificans PD1222
0.00	1	0	G	92944	Ketogulonicigenium
0.00	1	1	S	92945	Ketogulonicigenium vulgare
0.00	1	0	G	97050	Ruegeria
0.00	1	0	S	89184	Ruegeria pomeroyi
0.00	1	1	0	246200	Ruegeria pomeroyi DSS-3
0.00	1	0	G	191028	Leisingera
0.00	1	0	S	133924	Leisingera methylohalidivorans
0.00	1	1	0	999552	Leisingera methylohalidivorans DSM 14336
0.00	1	0	G	309512	Dinoroseobacter
0.00	1	0	S	215813	Dinoroseobacter shibae
0.00	1	1	0	398580	Dinoroseobacter shibae DFL 12 = DSM 16493
0.00	2	0	F	69657	Hyphomonadaceae
0.00	1	0	G	85	Hyphomonas
0.00	1	0	S	81032	Hyphomonas neptunium
0.00	1	1	0	228405	Hyphomonas neptunium ATCC 15444
0.00	1	0	G	74317	Maricaulis
0.00	1	0	S	74318	Maricaulis maris
0.00	1	1	0	394221	Maricaulis maris MCS10
0.00	27	0	O	204457	Sphingomonadales
0.00	27	3	F	41297	Sphingomonadaceae
0.00	9	0	G	13687	Sphingomonas
0.00	7	0	S	160791	Sphingomonas wittichii
0.00	7	7	0	392499	Sphingomonas wittichii RW1
0.00	2	2	S	745310	Sphingomonas sp. MM-1
0.00	7	1	G	165695	Sphingobium
0.00	3	0	S	332056	Sphingobium japonicum
0.00	3	3	0	452662	Sphingobium japonicum UT26S
0.00	3	3	S	627192	Sphingobium sp. SYK-6
0.00	5	0	G	165696	Novosphingobium
0.00	3	3	S	702113	Novosphingobium sp. PP1Y
0.00	2	0	S	48935	Novosphingobium aromaticivorans
0.00	2	2	0	279238	Novosphingobium aromaticivorans DSM 12444
0.00	3	0	G	165697	Sphingopyxis
0.00	3	0	S	117207	Sphingopyxis alaskensis
0.00	3	3	0	317655	Sphingopyxis alaskensis RB2256
0.00	26	0	O	204441	Rhodospirillales
0.00	24	1	F	41295	Rhodospirillaceae
0.00	11	0	G	191	Azospirillum
0.00	7	3	S	193	Azospirillum lipoferum
0.00	3	3	0	862719	Azospirillum lipoferum 4B
0.00	1	1	0	137722	Azospirillum sp. B510
0.00	4	0	S	192	Azospirillum brasilense
0.00	4	4	0	1064539	Azospirillum brasilense Sp245
0.00	10	0	G	1081	Rhodospirillum
0.00	7	0	S	1084	Rhodospirillum photometricum
0.00	7	7	0	1150469	Rhodospirillum photometricum DSM 122
0.00	2	0	S	34018	Rhodospirillum centenum
0.00	2	2	0	414684	Rhodospirillum centenum SW
0.00	1	1	S	1085	Rhodospirillum rubrum
0.00	2	0	G	171436	Tistrella
0.00	2	0	S	171437	Tistrella mobilis
0.00	2	2	0	1110502	Tistrella mobilis KA081020-065
0.00	2	0	F	433	Acetobacteraceae
0.00	1	0	G	441	Gluconobacter
0.00	1	0	S	442	Gluconobacter oxydans
0.00	1	1	0	290633	Gluconobacter oxydans 621H
0.00	1	0	G	89583	Gluconacetobacter
0.00	1	0	S	33996	Gluconacetobacter diazotrophicus
0.00	1	1	0	272568	Gluconacetobacter diazotrophicus PA1 5

0.00	10	0	O	204458	Caulobacterales
0.00	10	1	F	76892	Caulobacteraceae
0.00	5	0	G	75	Caulobacter
0.00	4	4	S	366602	Caulobacter sp. K31
0.00	1	0	S	155892	Caulobacter vibrioides
0.00	1	1	0	190650	Caulobacter crescentus CB15
0.00	4	0	G	41275	Brevundimonas
0.00	4	0	S	74313	Brevundimonas subvibrioides
0.00	4	4	0	633149	Brevundimonas subvibrioides ATCC 15264
0.00	1	0	O	255473	Parvularculales
0.00	1	0	F	255474	Parvularculaceae
0.00	1	0	G	208215	Parvularcula
0.00	1	0	S	208216	Parvularcula bermudensis
0.00	1	1	0	314260	Parvularcula bermudensis HTCC2503
0.02	26746	5	C	28216	Betaproteobacteria
0.01	16886	2317	0	119066	unclassified Betaproteobacteria
0.01	9358	0	G	33055	Kinetoplastibacterium
0.01	8016	8016	S	33056	Candidatus Kinetoplastibacterium crithidii
0.00	1338	0	S	994696	Candidatus Kinetoplastibacterium oncopeltii
0.00	1338	1338	0	1208920	Candidatus Kinetoplastibacterium oncopeltii TCC290E
0.00	3	0	S	994695	Candidatus Kinetoplastibacterium galatii
0.00	3	3	0	1208921	Candidatus Kinetoplastibacterium galatii TCC219
0.00	1	0	S	994692	Candidatus Kinetoplastibacterium desouzaii
0.00	1	1	0	1208919	Candidatus Kinetoplastibacterium desouzaii TCC079E
0.00	5203	0	G	1381133	Candidatus Profftella
0.00	5203	5203	S	669502	Candidatus Profftella armatura
0.00	5	0	G	1301080	Candidatus Nasuia
0.00	5	0	S	1160784	Candidatus Nasuia deltocephalinicola
0.00	5	5	0	1343077	Candidatus Nasuia deltocephalinicola str. NAS-ALF
0.00	2	0	G	327159	Candidatus Accumulibacter
0.00	2	0	S	327160	Candidatus Accumulibacter phosphatis
0.00	2	2	0	522306	Candidatus Accumulibacter phosphatis clade IIA str. UW-1
0.00	1	0	G	189384	Candidatus Tremblaya
0.00	1	1	S	189385	Candidatus Tremblaya princeps
0.01	9846	14	O	80840	Burkholderiales
0.01	7274	0	F	75682	Oxalobacteraceae
0.01	7272	0	G	884214	Candidatus Zinderia
0.01	7272	0	S	884215	Candidatus Zinderia insecticola
0.01	7272	7272	0	871271	Candidatus Zinderia insecticola CARI
0.00	2	0	G	963	Herbaspirillum
0.00	2	0	S	964	Herbaspirillum seropedicae
0.00	2	2	0	757424	Herbaspirillum seropedicae SmR1
0.00	1875	0	F	506	Alcaligenaceae
0.00	1873	0	G	222	Achromobacter
0.00	1873	1	S	85698	Achromobacter xylosoxidans
0.00	1398	1398	0	1216976	Achromobacter xylosoxidans NBRC 15126 = ATCC 27061
0.00	474	474	0	762376	Achromobacter xylosoxidans A8
0.00	1	0	G	517	Bordetella
0.00	1	0	S	94624	Bordetella petrii
0.00	1	1	0	340100	Bordetella petrii DSM 12804
0.00	1	0	G	290425	Advenella
0.00	1	0	S	310575	Advenella kashmirensis
0.00	1	1	0	1036672	Advenella kashmirensis WT001
0.00	609	23	F	80864	Comamonadaceae
0.00	498	223	G	80865	Delftia
0.00	143	143	S	742013	Delftia sp. Cs1-4
0.00	132	0	S	80866	Delftia acidovorans
0.00	132	132	0	398578	Delftia acidovorans SPH-1
0.00	71	9	G	12916	Acidovorax
0.00	50	50	S	358220	Acidovorax sp. KKS102
0.00	6	6	S	232721	Acidovorax sp. JS42
0.00	2	0	S	80867	Acidovorax avenae
0.00	2	0	0	80870	Acidovorax avenae subsp. avenae
0.00	2	2	0	643561	Acidovorax avenae subsp. avenae ATCC 19860
0.00	2	0	S	80869	Acidovorax citrulli
0.00	2	2	0	397945	Acidovorax citrulli AAC00-1
0.00	2	0	S	721785	Acidovorax ebreus
0.00	2	2	0	535289	Acidovorax ebreus TPSY
0.00	6	0	G	174951	Ramlibacter
0.00	6	0	S	94132	Ramlibacter tataouinensis
0.00	6	6	0	365046	Ramlibacter tataouinensis TTB310
0.00	4	0	G	34072	Variovorax
0.00	4	2	S	34073	Variovorax paradoxus
0.00	1	1	0	543728	Variovorax paradoxus S110
0.00	1	1	0	595537	Variovorax paradoxus EPS
0.00	3	0	G	201096	Alicycliphilus
0.00	3	3	S	179636	Alicycliphilus denitrificans
0.00	2	0	G	283	Comamonas
0.00	2	0	S	285	Comamonas testosteroni
0.00	2	0	0	543891	Comamonas testosteroni CNB-1
0.00	2	2	0	688245	Comamonas testosteroni CNB-2
0.00	1	0	G	28065	Rhodoferax
0.00	1	0	S	192843	Rhodoferax ferrireducens

0.00	1	1	0	338969	Rhodoferax ferrireducens T118
0.00	1	0	G	364316	Verminephrobacter
0.00	1	0	S	364317	Verminephrobacter eiseniae
0.00	1	1	0	391735	Verminephrobacter eiseniae EF01-2
0.00	68	0	F	119060	Burkholderiaceae
0.00	51	0	G	32008	Burkholderia
0.00	28	0	S	252970	Burkholderia phenoliruptrix
0.00	28	28	0	1229205	Burkholderia phenoliruptrix BR3459a
0.00	14	14	S	416344	Burkholderia sp. KJ006
0.00	3	1	0	87882	Burkholderia cepacia complex
0.00	1	0	S	95486	Burkholderia cenocepacia
0.00	1	1	0	406425	Burkholderia cenocepacia MC0-3
0.00	1	1	S	482957	Burkholderia lata
0.00	2	0	0	111527	pseudomallei group
0.00	2	2	S	28450	Burkholderia pseudomallei
0.00	1	0	S	337	Burkholderia glumae
0.00	1	1	0	626418	Burkholderia glumae BGR1
0.00	1	0	S	28095	Burkholderia gladioli
0.00	1	1	0	999541	Burkholderia gladioli BSR3
0.00	1	0	S	148447	Burkholderia phymatum
0.00	1	1	0	391038	Burkholderia phymatum STM815
0.00	1	0	S	261302	Burkholderia phytofirmans
0.00	1	1	0	398527	Burkholderia phytofirmans PsJN
0.00	14	0	G	48736	Ralstonia
0.00	13	3	S	329	Ralstonia pickettii
0.00	7	7	0	402626	Ralstonia pickettii 12J
0.00	2	2	0	428406	Ralstonia pickettii 12D
0.00	1	1	0	1366050	Ralstonia pickettii DTP0602
0.00	1	1	S	305	Ralstonia solanacearum
0.00	3	0	G	106589	Cupriavidus
0.00	1	0	S	106590	Cupriavidus necator
0.00	1	1	0	381666	Ralstonia eutropha H16
0.00	1	0	S	164546	Cupriavidus taiwanensis
0.00	1	1	0	977880	Cupriavidus taiwanensis LMG 19424
0.00	1	0	S	248026	Cupriavidus pinatubonensis
0.00	1	1	0	264198	Ralstonia eutropha JMP134
0.00	6	0	0	119065	unclassified Burkholderiales
0.00	6	0	0	224471	Burkholderiales Genera incertae sedis
0.00	3	0	G	28067	Rubrivivax
0.00	3	0	S	28068	Rubrivivax gelatinosus
0.00	3	3	0	983917	Rubrivivax gelatinosus IL144
0.00	2	0	G	316612	Methylibium
0.00	2	0	S	105560	Methylibium petroleiphilum
0.00	2	2	0	420662	Methylibium petroleiphilum PM1
0.00	1	0	G	88	Leptothrix
0.00	1	0	S	34029	Leptothrix cholodnii
0.00	1	1	0	395495	Leptothrix cholodnii SP-6
0.00	6	0	O	206389	Rhodocyclales
0.00	6	1	F	75787	Rhodocyclaceae
0.00	2	0	G	33057	Thauera
0.00	2	2	S	85643	Thauera sp. MZ1T
0.00	2	0	G	551759	Aromatoleum
0.00	2	0	S	551760	Aromatoleum aromaticum
0.00	2	2	0	76114	Aromatoleum aromaticum EbN1
0.00	1	0	G	73029	Dechloromonas
0.00	1	0	S	259537	Dechloromonas aromatica
0.00	1	1	0	159087	Dechloromonas aromatica RCB
0.00	2	0	O	206350	Methylophilales
0.00	2	1	F	32011	Methylophilaceae
0.00	1	0	G	404	Methylobacillus
0.00	1	0	S	405	Methylobacillus flagellatus
0.00	1	1	0	265072	Methylobacillus flagellatus KT
0.00	1	0	O	206351	Neisseriales
0.00	1	0	F	1499392	Chromobacteriaceae
0.00	1	0	G	568394	Pseudogulbenkiania
0.00	1	1	S	748280	Pseudogulbenkiania sp. NH8B
0.00	4911	0	0	68525	delta/epsilon subdivisions
0.00	4399	0	C	29547	Epsilonproteobacteria
0.00	4399	0	O	213849	Campylobacterales
0.00	3748	0	F	72294	Campylobacteraceae
0.00	3726	0	G	194	Campylobacter
0.00	3726	3725	S	197	Campylobacter jejuni
0.00	1	0	0	32022	Campylobacter jejuni subsp. jejuni
0.00	1	1	0	1201032	Campylobacter jejuni subsp. jejuni PT14
0.00	22	0	G	28196	Arcobacter
0.00	22	22	S	944547	Arcobacter sp. L
0.00	651	0	F	72293	Helicobacteraceae
0.00	610	0	G	209	Helicobacter
0.00	581	2	S	210	Helicobacter pylori
0.00	492	492	0	85962	Helicobacter pylori 26695
0.00	35	35	0	1055531	Helicobacter pylori Aklavik117
0.00	28	28	0	592205	Helicobacter pylori B38
0.00	12	12	0	907237	Helicobacter pylori Lithuania75

0.00	12	12	0	1352356	Helicobacter pylori SouthAfrica20
0.00	25	0	S	213	Helicobacter cinaedi
0.00	25	25	0	1172562	Helicobacter cinaedi PAGU611
0.00	4	0	S	138563	Helicobacter cetorum
0.00	3	3	0	182217	Helicobacter cetorum MIT 00-7128
0.00	1	1	0	1163745	Helicobacter cetorum MIT 99-5656
0.00	39	0	G	286130	Sulfuricurvum
0.00	39	0	S	148813	Sulfuricurvum kujiense
0.00	39	39	0	709032	Sulfuricurvum kujiense DSM 16994
0.00	2	0	G	202746	Sulfurimonas
0.00	2	0	S	202747	Sulfurimonas autotrophica
0.00	2	2	0	563040	Sulfurimonas autotrophica DSM 16294
0.00	512	1	C	28221	Deltaproteobacteria
0.00	253	0	O	213115	Desulfovibrionales
0.00	253	0	F	194924	Desulfovibrionaceae
0.00	250	0	G	41707	Lawsonia
0.00	250	171	S	29546	Lawsonia intracellularis
0.00	41	41	0	363253	Lawsonia intracellularis PHE/MN1-00
0.00	38	38	0	1234378	Lawsonia intracellularis N343
0.00	3	0	G	872	Desulfovibrio
0.00	2	0	S	881	Desulfovibrio vulgaris
0.00	2	2	0	883	Desulfovibrio vulgaris str. 'Miyazaki F'
0.00	1	0	S	876	Desulfovibrio desulfuricans
0.00	1	1	0	641491	Desulfovibrio desulfuricans ND132
0.00	154	0	O	29	Myxococcales
0.00	101	0	0	80811	Cystobacterineae
0.00	100	0	F	1524215	Anaeromyxobacteraceae
0.00	100	0	G	161492	Anaeromyxobacter
0.00	100	100	S	404589	Anaeromyxobacter sp. Fw109-5
0.00	1	0	F	31	Myxococcaceae
0.00	1	0	G	32	Myxococcus
0.00	1	0	S	33	Myxococcus fulvus
0.00	1	1	0	483219	Myxococcus fulvus HW-1
0.00	53	0	0	80812	Sorangiineae
0.00	53	0	F	49	Polyangiaceae
0.00	53	0	G	39643	Sorangium
0.00	53	0	S	56	Sorangium cellulosum
0.00	53	53	0	1254432	Sorangium cellulosum So0157-2
0.00	65	0	O	453227	Desulfarculales
0.00	65	0	F	453228	Desulfarculaceae
0.00	65	0	G	453229	Desulfarculus
0.00	65	0	S	453230	Desulfarculus baarsii
0.00	65	65	0	644282	Desulfarculus baarsii DSM 2075
0.00	23	0	O	69541	Desulfuromonadales
0.00	23	0	F	213422	Geobacteraceae
0.00	23	0	G	28231	Geobacter
0.00	12	12	S	443144	Geobacter sp. M21
0.00	11	0	S	351604	Geobacter uraniireducens
0.00	11	11	0	351605	Geobacter uraniireducens Rf4
0.00	15	0	O	213481	Bdellovibrionales
0.00	15	0	F	263369	Bacteriovoracaceae
0.00	15	0	G	146784	Bacteriovorax
0.00	15	0	S	97084	Bacteriovorax marinus
0.00	15	15	0	862908	Bacteriovorax marinus SJ
0.00	1	0	O	213118	Desulfobacterales
0.00	1	0	F	213121	Desulfobulbaceae
0.00	1	0	G	893	Desulfobulbus
0.00	1	0	S	894	Desulfobulbus propionicus
0.00	1	1	0	577650	Desulfobulbus propionicus DSM 2032
0.02	22444	25	P	1239	Firmicutes
0.01	16100	11	C	91061	Bacilli
0.01	12903	20	O	186826	Lactobacillales
0.00	4298	0	F	81852	Enterococcaceae
0.00	4297	4297	G	1350	Enterococcus
0.00	1	0	G	51668	Tetragenococcus
0.00	1	0	S	51669	Tetragenococcus halophilus
0.00	1	1	0	945021	Tetragenococcus halophilus NBRC 12172
0.00	4278	0	F	81850	Leuconostocaceae
0.00	4278	4277	G	1243	Leuconostoc
0.00	1	0	S	1252	Leuconostoc carnosum
0.00	1	1	0	1229758	Leuconostoc carnosum JB16
0.00	3354	1	F	1300	Streptococcaceae
0.00	3337	22	G	1301	Streptococcus
0.00	3130	0	0	671232	Streptococcus anginosus group
0.00	3130	3129	S	1328	Streptococcus anginosus
0.00	1	1	0	862970	Streptococcus anginosus C1051
0.00	58	18	S	1318	Streptococcus parasanguinis
0.00	20	20	0	760570	Streptococcus parasanguinis ATCC 15912
0.00	20	20	0	1114965	Streptococcus parasanguinis FW213
0.00	56	0	0	119603	Streptococcus dysgalactiae group
0.00	55	0	S	1336	Streptococcus equi
0.00	55	55	0	40041	Streptococcus equi subsp. zooepidemicus
0.00	1	0	S	1334	Streptococcus dysgalactiae

0.00	1	1	0	119602	Streptococcus dysgalactiae subsp. equisimilis
0.00	16	13	S	1313	Streptococcus pneumoniae
0.00	1	1	0	189423	Streptococcus pneumoniae 670-6B
0.00	1	1	0	488222	Streptococcus pneumoniae JJA
0.00	1	1	0	869309	Streptococcus pneumoniae SPNA45
0.00	15	0	S	28037	Streptococcus mitis
0.00	15	15	0	365659	Streptococcus mitis B6
0.00	10	0	S	1303	Streptococcus oralis
0.00	10	10	0	927666	Streptococcus oralis Uo5
0.00	9	9	S	1308	Streptococcus thermophilus
0.00	7	0	S	1305	Streptococcus sanguinis
0.00	7	7	0	388919	Streptococcus sanguinis SK36
0.00	3	2	S	1304	Streptococcus salivarius
0.00	1	1	0	347253	Streptococcus salivarius JIM8777
0.00	3	0	S	257758	Streptococcus pseudopneumoniae
0.00	3	3	0	1054460	Streptococcus pseudopneumoniae IS7493
0.00	2	0	S	1302	Streptococcus gordonii
0.00	2	0	0	29390	Streptococcus gordonii str. Challis
0.00	2	2	0	467705	Streptococcus gordonii str. Challis substr. CH1
0.00	2	0	S	102684	Streptococcus infantarius
0.00	2	0	0	150054	Streptococcus infantarius subsp. infantarius
0.00	2	2	0	1069533	Streptococcus infantarius subsp. infantarius CJ18
0.00	1	1	S	1307	Streptococcus suis
0.00	1	1	S	1309	Streptococcus mutans
0.00	1	0	S	59310	Streptococcus macedonicus
0.00	1	1	0	1116231	Streptococcus macedonicus ACA-DC 198
0.00	1	1	S	1156433	Streptococcus sp. I-P16
0.00	16	0	G	1357	Lactococcus
0.00	16	0	S	1358	Lactococcus lactis
0.00	9	6	0	1359	Lactococcus lactis subsp. cremoris
0.00	2	2	0	1111678	Lactococcus lactis subsp. cremoris UC509.9
0.00	1	1	0	272622	Lactococcus lactis subsp. cremoris SK11
0.00	7	5	0	1360	Lactococcus lactis subsp. lactis
0.00	1	1	0	684738	Lactococcus lactis subsp. lactis KF147
0.00	1	1	0	929102	Lactococcus lactis subsp. lactis CV56
0.00	953	0	F	33958	Lactobacillaceae
0.00	953	0	G	1578	Lactobacillus
0.00	944	0	S	1623	Lactobacillus ruminis
0.00	944	944	0	1069534	Lactobacillus ruminis ATCC 27782
0.00	2	2	S	47715	Lactobacillus rhamnosus
0.00	2	0	0	655183	Lactobacillus casei group
0.00	2	1	S	1582	Lactobacillus casei
0.00	1	1	0	498216	Lactobacillus casei str. Zhang
0.00	1	1	S	1579	Lactobacillus acidophilus
0.00	1	0	S	1584	Lactobacillus delbrueckii
0.00	1	1	0	1585	Lactobacillus delbrueckii subsp. bulgaricus
0.00	1	1	S	1590	Lactobacillus plantarum
0.00	1	0	S	1598	Lactobacillus reuteri
0.00	1	1	0	1340495	Lactobacillus reuteri I5007
0.00	1	0	S	47770	Lactobacillus crispatus
0.00	1	1	0	748671	Lactobacillus crispatus ST1
0.00	3186	8	O	1385	Bacillales
0.00	2607	1	F	186817	Bacillaceae
0.00	2576	3	G	1386	Bacillus
0.00	2358	273	0	86661	Bacillus cereus group
0.00	1677	17	S	1428	Bacillus thuringiensis
0.00	1511	0	0	1432	Bacillus thuringiensis serovar thuringiensis
0.00	1511	1511	0	1286404	Bacillus thuringiensis serovar thuringiensis str. IS5056
0.00	106	106	0	529122	Bacillus thuringiensis YBT-1518
0.00	30	2	0	29339	Bacillus thuringiensis serovar kurstaki
0.00	26	26	0	714359	Bacillus thuringiensis BMB171
0.00	2	2	0	1279365	Bacillus thuringiensis serovar kurstaki str. HD73
0.00	12	12	0	412694	Bacillus thuringiensis str. Al Hakam
0.00	1	0	0	180856	Bacillus thuringiensis serovar konkukian
0.00	1	1	0	281309	Bacillus thuringiensis serovar konkukian str. 97-27
0.00	300	0	S	86662	Bacillus weihenstephanensis
0.00	300	300	0	315730	Bacillus weihenstephanensis KBAB4
0.00	70	35	S	1396	Bacillus cereus
0.00	18	18	0	226900	Bacillus cereus ATCC 14579
0.00	11	11	0	405532	Bacillus cereus B4264
0.00	2	2	0	361100	Bacillus cereus Q1
0.00	2	0	0	1179100	Bacillus cereus biovar anthracis
0.00	2	2	0	637380	Bacillus cereus biovar anthracis str. CI
0.00	1	1	0	288681	Bacillus cereus E33L
0.00	1	1	0	405535	Bacillus cereus AH820
0.00	38	38	S	1392	Bacillus anthracis
0.00	194	0	S	324767	Bacillus infantis
0.00	194	194	0	1367477	Bacillus infantis NRRL B-14911
0.00	17	0	S	79885	Bacillus pseudofirmus
0.00	17	17	0	398511	Bacillus pseudofirmus OF4
0.00	3	0	0	653685	Bacillus subtilis group
0.00	2	1	S	1390	Bacillus amyloliquefaciens
0.00	1	0	0	1054400	Bacillus amyloliquefaciens subsp. plantarum

0.00	1	1	0	1338518	Bacillus amyloliquefaciens subsp. plantarum UCMB5033
0.00	1	0	S	1423	Bacillus subtilis
0.00	1	1	0	936156	Bacillus subtilis BSn5
0.00	1	0	S	1413	Bacillus cellulosilyticus
0.00	1	1	0	649639	Bacillus cellulosilyticus DSM 2522
0.00	11	0	G	150247	Anoxybacillus
0.00	11	0	S	33934	Anoxybacillus flavithermus
0.00	11	11	0	491915	Anoxybacillus flavithermus WK1
0.00	10	8	G	129337	Geobacillus
0.00	1	0	S	33940	Geobacillus thermodenitrificans
0.00	1	1	0	420246	Geobacillus thermodenitrificans NG80-2
0.00	1	1	S	1233873	Geobacillus sp. GHH01
0.00	8	0	G	400634	Lysinibacillus
0.00	8	0	S	1421	Lysinibacillus sphaericus
0.00	8	8	0	444177	Lysinibacillus sphaericus C3-41
0.00	1	0	G	182709	Oceanobacillus
0.00	1	0	S	182710	Oceanobacillus iheyensis
0.00	1	1	0	221109	Oceanobacillus iheyensis HTE831
0.00	555	7	F	90964	Staphylococcaceae
0.00	543	88	G	1279	Staphylococcus
0.00	284	0	S	29385	Staphylococcus saprophyticus
0.00	284	0	0	147452	Staphylococcus saprophyticus subsp. saprophyticus
0.00	284	284	0	342451	Staphylococcus saprophyticus subsp. saprophyticus ATCC 15305
0.00	57	44	S	1282	Staphylococcus epidermidis
0.00	7	7	0	176280	Staphylococcus epidermidis ATCC 12228
0.00	6	6	0	176279	Staphylococcus epidermidis RP62A
0.00	37	0	S	1292	Staphylococcus warneri
0.00	37	37	0	1194526	Staphylococcus warneri SG1
0.00	33	13	S	1280	Staphylococcus aureus
0.00	20	6	0	46170	Staphylococcus aureus subsp. aureus
0.00	8	8	0	985002	Staphylococcus aureus subsp. aureus MSHR1132
0.00	5	5	0	985006	Staphylococcus aureus subsp. aureus LGA251
0.00	1	1	0	1392476	Staphylococcus aureus subsp. aureus 6850
0.00	19	19	S	28035	Staphylococcus lugdunensis
0.00	9	0	S	1281	Staphylococcus carnosus
0.00	9	0	0	147448	Staphylococcus carnosus subsp. carnosus
0.00	9	9	0	396513	Staphylococcus carnosus subsp. carnosus TM300
0.00	8	0	S	45972	Staphylococcus pasteuri
0.00	8	8	0	1276282	Staphylococcus pasteuri SP1
0.00	7	0	S	1283	Staphylococcus haemolyticus
0.00	7	7	0	279808	Staphylococcus haemolyticus JCSC1435
0.00	1	1	S	283734	Staphylococcus pseudintermedius
0.00	5	0	G	69965	Macrococcus
0.00	5	0	S	69966	Macrococcus caseolyticus
0.00	5	5	0	458233	Macrococcus caseolyticus JCSC5402
0.00	14	0	F	186820	Listeriaceae
0.00	14	2	G	1637	Listeria
0.00	12	2	S	1639	Listeria monocytogenes
0.00	9	9	0	882097	Listeria monocytogenes SLCC2376
0.00	1	1	0	879089	Listeria monocytogenes SLCC2540
0.00	1	0	F	186821	Sporolactobacillaceae
0.00	1	0	0	663587	unclassified Sporolactobacillaceae
0.00	1	0	S	85683	[Bacillus] selenitireducens
0.00	1	1	0	439292	[Bacillus] selenitireducens MLS10
0.00	1	0	F	186822	Paenibacillaceae
0.00	1	0	G	44249	Paenibacillus
0.00	1	0	S	61624	Paenibacillus mucilaginosus
0.00	1	1	0	997761	Paenibacillus mucilaginosus K02
0.01	6296	75	C	186801	Clostridia
0.00	4315	10	O	186802	Clostridiales
0.00	3945	0	F	31979	Clostridiaceae
0.00	3632	0	G	49082	Candidatus Arthromitus
0.00	3632	3632	S	49118	Candidatus Arthromitus sp. SFB-mouse
0.00	278	1	G	1485	Clostridium
0.00	205	0	S	1501	Clostridium pasteurianum
0.00	205	205	0	86416	Clostridium pasteurianum BC1
0.00	51	51	S	1534	Clostridium kluyveri
0.00	12	0	S	1491	Clostridium botulinum
0.00	10	0	0	36827	Clostridium botulinum B
0.00	10	0	0	591968	Clostridium botulinum B1
0.00	10	10	0	498213	Clostridium botulinum B1 str. Okra
0.00	1	1	0	36826	Clostridium botulinum A
0.00	1	1	0	929506	Clostridium botulinum BKT015925
0.00	4	0	S	1493	Clostridium cellulovorans
0.00	4	4	0	573061	Clostridium cellulovorans 743B
0.00	4	0	S	169679	Clostridium saccharobutylicum
0.00	4	4	0	1345695	Clostridium saccharobutylicum DSM 13864
0.00	1	1	S	1488	Clostridium acetobutylicum
0.00	35	0	G	114627	Alkaliphilus
0.00	35	0	S	208226	Alkaliphilus metalliredigens
0.00	35	35	0	293826	Alkaliphilus metalliredigens QYMF
0.00	327	0	F	186804	Peptostreptococcaceae
0.00	327	0	G	1481960	Peptoclostridium

0.00	327	326	S	1496	Peptoclostridium difficile
0.00	1	1	0	272563	Peptoclostridium difficile 630
0.00	8	0	F	186803	Lachnospiraceae
0.00	5	0	G	1506553	Lachnoclostridium
0.00	5	0	S	66219	Lachnoclostridium phytofermentans
0.00	5	5	0	357809	Lachnoclostridium phytofermentans ISDg
0.00	1	0	G	841	Roseburia
0.00	1	1	S	166486	Roseburia intestinalis
0.00	1	0	G	33042	Coprococcus
0.00	1	0	S	116085	Coprococcus catus
0.00	1	1	0	717962	Coprococcus catus GD/7
0.00	1	0	G	572511	Blautia
0.00	1	0	S	40520	[Ruminococcus] obeum
0.00	1	1	0	657314	Ruminococcus obeum A2-162
0.00	8	0	0	1570339	Peptoniphilaceae
0.00	6	0	G	150022	Finegoldia
0.00	6	0	S	1260	Finegoldia magna
0.00	6	6	0	334413	Finegoldia magna ATCC 29328
0.00	2	0	G	165779	Anaerococcus
0.00	2	0	S	33034	Anaerococcus prevotii
0.00	2	2	0	525919	Anaerococcus prevotii DSM 20548
0.00	7	0	F	68298	Syntrophomonadaceae
0.00	6	0	G	862	Syntrophomonas
0.00	6	0	S	863	Syntrophomonas wolfei
0.00	6	0	0	370885	Syntrophomonas wolfei subsp. wolfei
0.00	6	6	0	335541	Syntrophomonas wolfei subsp. wolfei str. Goettingen G311
0.00	1	0	G	129001	Syntrophothermus
0.00	1	0	S	86170	Syntrophothermus lipocalidus
0.00	1	1	0	643648	Syntrophothermus lipocalidus DSM 12680
0.00	4	0	F	186807	Peptococcaceae
0.00	4	0	G	1562	Desulfotomaculum
0.00	4	0	S	58135	Desulfotomaculum kuznetsovii
0.00	4	4	0	760568	Desulfotomaculum kuznetsovii DSM 6115
0.00	4	0	F	541000	Ruminococcaceae
0.00	2	0	G	1263	Ruminococcus
0.00	1	0	S	40518	Ruminococcus bromii
0.00	1	1	0	657321	Ruminococcus bromii L2-63
0.00	1	1	S	657323	Ruminococcus sp. SR1/5
0.00	2	0	G	1508657	Ruminiclostridium
0.00	2	1	S	39492	[Eubacterium] siraeum
0.00	1	1	0	717961	[Eubacterium] siraeum V10Sc8a
0.00	2	0	F	186806	Eubacteriaceae
0.00	2	0	G	1730	Eubacterium
0.00	2	1	S	39491	Eubacterium rectale
0.00	1	1	0	657317	Eubacterium rectale M104/1
0.00	1905	1773	O	68295	Thermoanaerobacterales
0.00	119	0	F	186814	Thermoanaerobacteraceae
0.00	64	55	G	1754	Thermoanaerobacter
0.00	9	0	S	46354	Thermoanaerobacter wiegelii
0.00	9	9	0	697303	Thermoanaerobacter wiegelii Rt8.B1
0.00	55	0	G	249529	Caldanaerobacter
0.00	55	0	S	911092	Caldanaerobacter subterraneus
0.00	55	0	0	119072	Caldanaerobacter subterraneus subsp. tengcongensis
0.00	55	55	0	273068	Caldanaerobacter subterraneus subsp. tengcongensis MB4
0.00	13	0	F	543371	Thermoanaerobacterales Family III. Incertae Sedis
0.00	13	0	G	44000	Caldicellulosiruptor
0.00	8	0	S	31899	Caldicellulosiruptor bescii
0.00	8	8	0	521460	Caldicellulosiruptor bescii DSM 6725
0.00	5	0	S	413889	Caldicellulosiruptor kronotskyensis
0.00	5	5	0	632348	Caldicellulosiruptor kronotskyensis 2002
0.00	1	0	O	53433	Halanaerobiales
0.00	1	0	F	972	Halanaerobiaceae
0.00	1	0	G	2330	Halanaerobium
0.00	1	1	S	656519	Halanaerobium hydrogeniformans
0.00	23	0	C	909932	Negativicutes
0.00	23	0	O	909929	Selenomonadales
0.00	23	0	F	31977	Veillonellaceae
0.00	22	0	G	29465	Veillonella
0.00	22	0	S	29466	Veillonella parvula
0.00	22	22	0	479436	Veillonella parvula DSM 2008
0.00	1	0	G	158846	Megamonas
0.00	1	0	S	158847	Megamonas hypermegale
0.00	1	1	0	657316	Megamonas hypermegale ART12/1
0.01	17414	0	P	544448	Tenericutes
0.01	17414	0	C	31969	Mollicutes
0.01	17364	0	O	2085	Mycoplasmatales
0.01	17364	0	F	2092	Mycoplasmataceae
0.01	17364	158	G	2093	Mycoplasma
0.01	8881	8871	S	2100	Mycoplasma hyorhinis
0.00	8	8	0	1118964	Mycoplasma hyorhinis SK76
0.00	2	2	0	872331	Mycoplasma hyorhinis HUB-1
0.00	5766	70	S	2099	Mycoplasma hyopneumoniae
0.00	2650	14	0	907287	Mycoplasma hyopneumoniae 168

0.00	2636	2636	0	1116211	Mycoplasma hyopneumoniae 168-L
0.00	1551	1551	0	754503	Mycoplasma hyopneumoniae 7422
0.00	1383	1383	0	262722	Mycoplasma hyopneumoniae 7448
0.00	89	89	0	295358	Mycoplasma hyopneumoniae 232
0.00	23	23	0	262719	Mycoplasma hyopneumoniae J
0.00	2070	0	S	2109	Mycoplasma synoviae
0.00	2070	2070	0	262723	Mycoplasma synoviae 53
0.00	308	143	S	2096	Mycoplasma gallisepticum
0.00	153	153	0	1006581	Mycoplasma gallisepticum S6
0.00	12	12	0	1159201	Mycoplasma gallisepticum WI01_2001.043-13-2P
0.00	96	49	S	2097	Mycoplasma genitalium
0.00	45	45	0	662946	Mycoplasma genitalium M6282
0.00	2	2	0	662945	Mycoplasma genitalium M6320
0.00	45	0	S	136241	Mycoplasma haemocanis
0.00	45	45	0	1111676	Mycoplasma haemocanis str. Illinois
0.00	15	0	0	656088	Mycoplasma mycoides group
0.00	15	0	S	2102	Mycoplasma mycoides
0.00	12	0	0	40477	Mycoplasma mycoides subsp. capri
0.00	12	0	0	44100	Mycoplasma mycoides subsp. capri LC
0.00	12	12	0	862259	Mycoplasma mycoides subsp. capri LC str. 95010
0.00	3	0	0	2103	Mycoplasma mycoides subsp. mycoides
0.00	3	0	0	44101	Mycoplasma mycoides subsp. mycoides SC
0.00	3	3	0	865867	Mycoplasma mycoides subsp. mycoides SC str. Gladysdale
0.00	13	0	S	2107	Mycoplasma pulmonis
0.00	13	13	0	272635	Mycoplasma pulmonis UAB CTIP
0.00	4	0	S	57372	Mycoplasma suis
0.00	4	4	0	768700	Mycoplasma suis str. Illinois
0.00	4	0	S	171284	Mycoplasma cynos
0.00	4	4	0	1246955	Mycoplasma cynos C142
0.00	3	0	S	28903	Mycoplasma bovis
0.00	3	3	0	289397	Mycoplasma bovis PG45
0.00	1	0	S	2123	Mycoplasma putrefaciens
0.00	1	1	0	743965	Mycoplasma putrefaciens KS1
0.00	37	0	O	186329	Acholeplasmatales
0.00	37	0	F	2146	Acholeplasmataceae
0.00	36	0	G	33926	Candidatus Phytoplasma
0.00	36	0	0	85630	16SrX (Apple proliferation group)
0.00	36	36	S	37692	Candidatus Phytoplasma mali
0.00	1	0	G	2147	Acholeplasma
0.00	1	0	S	2148	Acholeplasma laidlawii
0.00	1	1	0	441768	Acholeplasma laidlawii PG-8A
0.00	13	0	O	186328	Entomoplasmatales
0.00	13	0	F	2131	Spiroplasmataceae
0.00	13	4	G	2132	Spiroplasma
0.00	7	0	S	2145	Spiroplasma taiwanense
0.00	7	7	0	1276220	Spiroplasma taiwanense CT-1
0.00	1	0	S	2137	Spiroplasma apis
0.00	1	1	0	1276258	Spiroplasma apis B31
0.00	1	0	S	216933	Spiroplasma chrysopicola
0.00	1	1	0	1276227	Spiroplasma chrysopicola DF-1
0.01	7285	0	0	2323	unclassified Bacteria
0.01	7240	0	0	49928	unclassified Bacteria (miscellaneous)
0.01	7240	7240	S	1235990	Halyomorpha halys symbiont
0.00	44	0	P	95818	Candidatus Saccharibacteria
0.00	44	0	G	1331051	Candidatus Saccharimonas
0.00	44	44	S	1332188	Candidatus Saccharimonas aalborgensis
0.00	1	0	0	221235	candidate division SR1
0.00	1	1	S	1394709	candidate division SR1 bacterium RAAC1_SR1_1
0.00	5504	0	0	68336	Bacteroidetes/Chlorobi group
0.00	5504	1	P	976	Bacteroidetes
0.00	5345	0	C	117743	Flavobacteriia
0.00	5345	0	O	200644	Flavobacteriales
0.00	5143	18	F	49546	Flavobacteriaceae
0.00	4598	0	G	237	Flavobacterium
0.00	3923	0	S	312277	Flavobacterium indicum
0.00	3923	3923	0	1094466	Flavobacterium indicum GPTSA100-9 = DSM 17447
0.00	648	0	S	96345	Flavobacterium psychrophilum
0.00	648	648	0	402612	Flavobacterium psychrophilum JIP02/86
0.00	24	0	S	986	Flavobacterium johnsoniae
0.00	24	24	0	376686	Flavobacterium johnsoniae UW101
0.00	3	0	S	996	Flavobacterium columnare
0.00	3	3	0	1041826	Flavobacterium columnare ATCC 49512
0.00	403	0	G	112040	Zobellia
0.00	403	403	S	63186	Zobellia galactanivorans
0.00	119	0	G	104264	Cellulophaga
0.00	119	0	S	59600	Cellulophaga algicola
0.00	119	119	0	688270	Cellulophaga algicola DSM 14237
0.00	2	0	G	52959	Polaribacter
0.00	2	2	S	313598	Polaribacter sp. MED152
0.00	1	0	G	34084	Riemerella
0.00	1	1	S	34085	Riemerella anatipestifer
0.00	1	0	0	61432	unclassified Flavobacteriaceae
0.00	1	1	S	531844	Flavobacteriaceae bacterium 3519-10

0.00	1	0	G	292691	Gramella
0.00	1	0	S	411153	Gramella forsetii
0.00	1	1	0	411154	Gramella forsetii KT0803
0.00	201	0	0	313602	unclassified Flavobacteriales
0.00	201	0	G	336809	Candidatus Sulcia
0.00	201	3	S	336810	Candidatus Sulcia muelleri
0.00	165	165	0	595499	Candidatus Sulcia muelleri SMDSEM
0.00	23	23	0	1343076	Candidatus Sulcia muelleri str. Sulcia-ALF
0.00	10	10	0	641892	Candidatus Sulcia muelleri DMIN
0.00	1	0	F	39782	Blattabacteriaceae
0.00	1	0	G	34098	Blattabacterium
0.00	1	1	S	1186051	Blattabacterium sp. (Blaberus giganteus)
0.00	80	0	C	200643	Bacteroidia
0.00	80	4	O	171549	Bacteroidales
0.00	48	0	F	815	Bacteroidaceae
0.00	48	0	G	816	Bacteroides
0.00	38	0	S	371601	Bacteroides xylanisolvens
0.00	38	38	0	657309	Bacteroides xylanisolvens XB1A
0.00	9	0	S	376805	Bacteroides salanitronis
0.00	9	9	0	667015	Bacteroides salanitronis DSM 18170
0.00	1	0	S	290053	Bacteroides helcogenes
0.00	1	1	0	693979	Bacteroides helcogenes P 36-108
0.00	28	0	F	171552	Prevotellaceae
0.00	28	0	G	838	Prevotella
0.00	25	0	S	28132	Prevotella melaninogenica
0.00	25	25	0	553174	Prevotella melaninogenica ATCC 25845
0.00	3	0	S	652716	Prevotella sp. oral taxon 299
0.00	3	3	0	575614	Prevotella sp. oral taxon 299 str. F0039
0.00	77	0	C	768503	Cytophagia
0.00	77	0	O	768507	Cytophagales
0.00	67	0	F	89373	Cytophagaceae
0.00	30	0	G	120831	Dyadobacter
0.00	30	0	S	94254	Dyadobacter fermentans
0.00	30	30	0	471854	Dyadobacter fermentans DSM 18053
0.00	16	0	G	978	Cytophaga
0.00	16	0	S	985	Cytophaga hutchinsonii
0.00	16	16	0	269798	Cytophaga hutchinsonii ATCC 33406
0.00	15	0	G	312278	Emticicia
0.00	15	0	S	312279	Emticicia oligotrophica
0.00	15	15	0	929562	Emticicia oligotrophica DSM 17448
0.00	5	0	G	861914	Fibrella
0.00	5	0	S	651143	Fibrella aestuarina
0.00	5	5	0	1166018	Fibrella aestuarina BUZ 2
0.00	1	0	G	319458	Leadbetterella
0.00	1	0	S	316068	Leadbetterella byssophila
0.00	1	1	0	649349	Leadbetterella byssophila DSM 17132
0.00	10	0	F	563798	Cyclobacteriaceae
0.00	10	0	G	68288	Cyclobacterium
0.00	10	0	S	104	Cyclobacterium marinum
0.00	10	10	0	880070	Cyclobacterium marinum DSM 745
0.00	1	0	C	117747	Sphingobacteriia
0.00	1	0	O	200666	Sphingobacteriales
0.00	1	0	0	563835	Chitinophagaceae
0.00	1	0	G	79328	Chitinophaga
0.00	1	0	S	79329	Chitinophaga pinensis
0.00	1	1	0	485918	Chitinophaga pinensis DSM 2588
0.00	4492	10	P	1117	Cyanobacteria
0.00	4178	0	O	1212	Prochlorales
0.00	4178	0	F	1217	Prochlorococcaceae
0.00	4178	0	G	1218	Prochlorococcus
0.00	4178	0	S	1219	Prochlorococcus marinus
0.00	4177	4177	0	74546	Prochlorococcus marinus str. MIT 9312
0.00	1	0	0	142554	Prochlorococcus marinus subsp. marinus
0.00	1	1	0	167539	Prochlorococcus marinus subsp. marinus str. CCMP1375
0.00	163	0	0	1301283	Oscillatoriophycideae
0.00	128	0	O	1118	Chroococcales
0.00	107	0	G	43988	Cyanothece
0.00	107	107	S	497965	Cyanothece sp. PCC 7822
0.00	21	0	0	92682	Halothece cluster
0.00	21	0	G	76023	Halothece
0.00	21	21	S	65093	Halothece sp. PCC 7418
0.00	35	0	O	1150	Oscillatoriales
0.00	21	0	G	1158	Oscillatoria
0.00	21	0	S	482564	Oscillatoria nigro-viridis
0.00	21	21	0	179408	Oscillatoria nigro-viridis PCC 7112
0.00	13	0	G	47251	Leptolyngbya
0.00	13	13	S	111781	Leptolyngbya sp. PCC 7376
0.00	1	0	G	1152	Pseudanabaena
0.00	1	1	S	82654	Pseudanabaena sp. PCC 7367
0.00	134	0	O	1161	Nostocales
0.00	86	0	F	1185	Rivulariaceae
0.00	44	0	G	373984	Rivularia
0.00	44	44	S	373994	Rivularia sp. PCC 7116

0.00	42	0	G	1186	Calothrix
0.00	42	0	S	32054	Calothrix parietina
0.00	42	42	0	1170562	Calothrix sp. PCC 6303
0.00	48	0	F	1162	Nostocaceae
0.00	44	0	G	1163	Anabaena
0.00	43	43	S	46234	Anabaena sp. 90
0.00	1	0	S	1165	Anabaena cylindrica
0.00	1	1	0	272123	Anabaena cylindrica PCC 7122
0.00	3	0	G	264688	Trichormus
0.00	3	0	S	1164	Trichormus azollae
0.00	3	3	0	551115	'Nostoc azollae' 0708
0.00	1	0	G	1177	Nostoc
0.00	1	0	S	272131	Nostoc punctiforme
0.00	1	1	0	63737	Nostoc punctiforme PCC 73102
0.00	7	0	O	52604	Pleurocapsales
0.00	7	0	G	54298	Chroococcidiopsis
0.00	7	0	S	54299	Chroococcidiopsis thermalis
0.00	7	7	0	251229	Chroococcidiopsis thermalis PCC 7203
0.00	1238	0	P	201174	Actinobacteria
0.00	1238	2	C	1760	Actinobacteria
0.00	1229	1	0	85003	Actinobacteridae
0.00	1224	69	O	2037	Actinomycetales
0.00	809	0	0	85009	Propionibacterineae
0.00	797	0	F	31957	Propionibacteriaceae
0.00	795	3	G	1743	Propionibacterium
0.00	779	606	S	1747	Propionibacterium acnes
0.00	154	154	0	1091045	Propionibacterium acnes ATCC 11828
0.00	16	16	0	909952	Propionibacterium acnes 266
0.00	2	2	0	1134454	Propionibacterium acnes HL096PA1
0.00	1	1	0	267747	Propionibacterium acnes KPA171202
0.00	7	0	S	33010	Propionibacterium avidum
0.00	7	7	0	1170318	Propionibacterium avidum 44067
0.00	3	0	S	1750	Propionibacterium propionicum
0.00	3	3	0	767029	Propionibacterium propionicum F0230a
0.00	2	0	S	1748	Propionibacterium acidipropionici
0.00	2	2	0	1171373	Propionibacterium acidipropionici ATCC 4875
0.00	1	0	S	1744	Propionibacterium freudenreichii
0.00	1	0	0	1752	Propionibacterium freudenreichii subsp. shermanii
0.00	1	1	0	754252	Propionibacterium freudenreichii subsp. shermanii CIRM-BIA1
0.00	2	0	G	29404	Microlunatus
0.00	2	0	S	29405	Microlunatus phosphovorus
0.00	2	2	0	1032480	Microlunatus phosphovorus NM-1
0.00	12	0	F	85015	Nocardioidaceae
0.00	10	0	G	1839	Nocardioides
0.00	10	10	S	196162	Nocardioides sp. JS614
0.00	2	0	G	182639	Kribbella
0.00	2	0	S	182640	Kribbella flavida
0.00	2	2	0	479435	Kribbella flavida DSM 17836
0.00	176	12	0	85006	Micrococcineae
0.00	72	2	F	1268	Micrococcaceae
0.00	44	0	G	1269	Micrococcus
0.00	44	0	S	1270	Micrococcus luteus
0.00	44	44	0	465515	Micrococcus luteus NCTC 2665
0.00	16	0	G	32207	Rothia
0.00	12	0	S	2047	Rothia dentocariosa
0.00	12	12	0	762948	Rothia dentocariosa ATCC 17931
0.00	4	0	S	43675	Rothia mucilaginosa
0.00	4	4	0	680646	Rothia mucilaginosa DY-18
0.00	8	3	G	1663	Arthrobacter
0.00	3	0	S	256701	Arthrobacter arilaitensis
0.00	3	3	0	861360	Arthrobacter arilaitensis Re117
0.00	1	0	S	43663	Arthrobacter aurescens
0.00	1	1	0	290340	Arthrobacter aurescens TC1
0.00	1	1	S	290399	Arthrobacter sp. FB24
0.00	2	0	G	57493	Kocuria
0.00	2	0	S	72000	Kocuria rhizophila
0.00	2	2	0	378753	Kocuria rhizophila DC2201
0.00	66	3	F	85023	Microbacteriaceae
0.00	57	0	G	33882	Microbacterium
0.00	57	0	S	2033	Microbacterium testaceum
0.00	57	57	0	979556	Microbacterium testaceum StLB037
0.00	5	0	G	1573	Clavibacter
0.00	5	2	S	28447	Clavibacter michiganensis
0.00	1	0	0	31963	Clavibacter michiganensis subsp. nebraskensis
0.00	1	1	0	1097677	Clavibacter michiganensis subsp. nebraskensis NCPPB 2581
0.00	1	1	0	31964	Clavibacter michiganensis subsp. sepedonicus
0.00	1	0	0	33013	Clavibacter michiganensis subsp. michiganensis
0.00	1	1	0	443906	Clavibacter michiganensis subsp. michiganensis NCPPB 382
0.00	1	0	G	110932	Leifsonia
0.00	1	1	S	1575	Leifsonia xyli
0.00	15	0	F	85020	Dermabacteraceae
0.00	15	0	G	43668	Brachybacterium
0.00	15	0	S	43669	Brachybacterium faecium

0.00	15	15	0	446465	Brachybacterium faecium DSM 4810
0.00	6	0	F	85016	Cellulomonadaceae
0.00	6	1	G	1707	Cellulomonas
0.00	3	0	S	1708	Cellulomonas fimi
0.00	3	3	0	590998	Cellulomonas fimi ATCC 484
0.00	2	0	S	11	[Cellvibrio] gilvus
0.00	2	2	0	593907	[Cellvibrio] gilvus ATCC 13127
0.00	2	0	F	85021	Intrasporangiaceae
0.00	2	0	G	53357	Intrasporangium
0.00	2	0	S	53358	Intrasporangium calvum
0.00	2	2	0	710696	Intrasporangium calvum DSM 43043
0.00	2	0	F	125316	Beutenbergiaceae
0.00	2	0	G	84756	Beutenbergia
0.00	2	0	S	84757	Beutenbergia cavernae
0.00	2	2	0	471853	Beutenbergia cavernae DSM 12333
0.00	1	0	F	145357	Dermacoccaceae
0.00	1	0	G	57499	Kytococcus
0.00	1	0	S	1276	Kytococcus sedentarius
0.00	1	1	0	478801	Kytococcus sedentarius DSM 20547
0.00	76	2	0	85007	Corynebacterineae
0.00	37	0	F	1762	Mycobacteriaceae
0.00	36	7	G	1763	Mycobacterium
0.00	13	1	0	77643	Mycobacterium tuberculosis complex
0.00	7	0	S	78331	Mycobacterium canettii
0.00	7	7	0	1205675	Mycobacterium canettii CIPT 140070008
0.00	5	0	S	1773	Mycobacterium tuberculosis
0.00	5	5	0	478434	Mycobacterium tuberculosis KZN 1435
0.00	4	2	S	1804	Mycobacterium gilvum
0.00	1	1	0	278137	Mycobacterium gilvum Spyr1
0.00	1	1	0	350054	Mycobacterium gilvum PYR-GCK
0.00	3	0	S	1772	Mycobacterium smegmatis
0.00	2	2	0	710686	Mycobacterium smegmatis JS623
0.00	1	1	0	246196	Mycobacterium smegmatis str. MC2 155
0.00	2	0	S	1800	Mycobacterium chubuense
0.00	2	2	0	710421	Mycobacterium chubuense NBB4
0.00	2	0	S	36814	Mycobacterium rhodesiae
0.00	2	2	0	710685	Mycobacterium rhodesiae NBB3
0.00	2	0	0	670516	Mycobacterium chelonae group
0.00	2	0	0	670506	Mycobacterium abscessus subgroup
0.00	2	2	S	36809	Mycobacterium abscessus
0.00	1	0	S	1768	Mycobacterium kansasii
0.00	1	1	0	557599	Mycobacterium kansasii ATCC 12478
0.00	1	0	S	110539	Mycobacterium vanbaalenii
0.00	1	1	0	350058	Mycobacterium vanbaalenii PYR-1
0.00	1	1	0	120793	Mycobacterium avium complex (MAC)
0.00	1	0	G	992401	Amycolicicoccus
0.00	1	0	S	639313	Amycolicicoccus subflavus
0.00	1	1	0	443218	Amycolicicoccus subflavus DQS3-9A1
0.00	17	0	F	1653	Corynebacteriaceae
0.00	17	0	G	1716	Corynebacterium
0.00	5	0	S	161879	Corynebacterium kroppenstedtii
0.00	5	5	0	645127	Corynebacterium kroppenstedtii DSM 44385
0.00	4	0	S	1727	Corynebacterium variabile
0.00	4	4	0	858619	Corynebacterium variabile DSM 44702
0.00	3	0	S	38289	Corynebacterium jeikeium
0.00	3	3	0	306537	Corynebacterium jeikeium K411
0.00	2	0	S	258224	Corynebacterium resistens
0.00	2	2	0	662755	Corynebacterium resistens DSM 45100
0.00	1	0	S	1718	Corynebacterium glutamicum
0.00	1	1	0	340322	Corynebacterium glutamicum R
0.00	1	0	S	169292	Corynebacterium aurimucosum
0.00	1	1	0	548476	Corynebacterium aurimucosum ATCC 700975
0.00	1	0	S	225326	Corynebacterium halotolerans
0.00	1	1	0	1121362	Corynebacterium halotolerans YIM 70093 = DSM 44683
0.00	15	0	F	85025	Nocardiaceae
0.00	12	1	G	1827	Rhodococcus
0.00	7	1	S	1833	Rhodococcus erythropolis
0.00	4	4	0	234621	Rhodococcus erythropolis PR4
0.00	2	2	0	1136179	Rhodococcus erythropolis CCM2595
0.00	2	0	S	37919	Rhodococcus opacus
0.00	2	2	0	632772	Rhodococcus opacus B4
0.00	1	0	S	43767	Rhodococcus hoagii
0.00	1	1	0	685727	Rhodococcus equi 103S
0.00	1	0	S	132919	Rhodococcus jostii
0.00	1	1	0	101510	Rhodococcus jostii RHA1
0.00	3	0	G	1817	Nocardia
0.00	2	0	S	37326	Nocardia brasiliensis
0.00	2	2	0	1133849	Nocardia brasiliensis ATCC 700358
0.00	1	0	S	135487	Nocardia cyriacigeorgica
0.00	1	1	0	1127134	Nocardia cyriacigeorgica GUH-2
0.00	2	0	F	85026	Gordoniaceae
0.00	2	0	G	2053	Gordonia
0.00	2	0	S	84595	Gordonia polyisoprenivorans

0.00	2	2	0	1112204	Gordonia polyisoprenivorans VH2
0.00	2	0	F	316606	Segniliparaceae
0.00	2	0	G	286801	Segniliparus
0.00	2	0	S	286802	Segniliparus rotundus
0.00	2	2	0	640132	Segniliparus rotundus DSM 44985
0.00	1	0	F	85028	Tsukamurellaceae
0.00	1	0	G	2060	Tsukamurella
0.00	1	0	S	2061	Tsukamurella paurometabola
0.00	1	1	0	521096	Tsukamurella paurometabola DSM 20162
0.00	32	0	0	85011	Streptomycineae
0.00	32	0	F	2062	Streptomycetaceae
0.00	29	5	G	1883	Streptomyces
0.00	4	0	S	68280	Streptomyces violaceusniger
0.00	4	4	0	653045	Streptomyces violaceusniger Tu 4113
0.00	4	0	0	1477431	Streptomyces albidoflavus group
0.00	4	0	S	1902	Streptomyces coelicolor
0.00	4	4	0	100226	Streptomyces coelicolor A3(2)
0.00	3	0	S	33903	Streptomyces avermitilis
0.00	3	3	0	227882	Streptomyces avermitilis MA-4680 = NBRC 14893
0.00	2	0	S	1930	Streptomyces scabiei
0.00	2	2	0	680198	Streptomyces scabiei 87.22
0.00	2	0	S	42684	Streptomyces collinus
0.00	2	2	0	1214242	Streptomyces collinus Tu 365
0.00	2	0	S	348043	Streptomyces davawensis
0.00	2	2	0	1214101	Streptomyces davawensis JCM 4913
0.00	2	0	S	1226757	Streptomyces rapamycinicus
0.00	2	2	0	1343740	Streptomyces rapamycinicus NRRL 5491
0.00	1	0	S	1912	Streptomyces hygroscopicus
0.00	1	1	0	311982	Streptomyces hygroscopicus subsp. jinggangensis
0.00	1	0	S	54571	Streptomyces venezuelae
0.00	1	1	0	953739	Streptomyces venezuelae ATCC 10712
0.00	1	0	S	68202	Streptomyces fulvissimus
0.00	1	1	0	1303692	Streptomyces fulvissimus DSM 40593
0.00	1	0	S	379067	Streptomyces bingchenggensis
0.00	1	1	0	749414	Streptomyces bingchenggensis BCW-1
0.00	1	1	S	862751	Streptomyces sp. SirexAA-E
0.00	3	0	G	2063	Kitasatospora
0.00	3	0	S	2066	Kitasatospora setae
0.00	3	3	0	452652	Kitasatospora setae KM-6054
0.00	20	1	0	85012	Streptosporangineae
0.00	19	0	F	83676	Nocardiopsaceae
0.00	17	1	G	2013	Nocardiopsis
0.00	9	0	S	53437	Nocardiopsis alba
0.00	9	9	0	1205910	Nocardiopsis alba ATCC BAA-2165
0.00	7	0	S	2014	Nocardiopsis dassonvillei
0.00	7	0	0	568208	Nocardiopsis dassonvillei subsp. dassonvillei
0.00	7	7	0	446468	Nocardiopsis dassonvillei subsp. dassonvillei DSM 43111
0.00	2	0	G	83677	Thermobifida
0.00	2	0	S	2021	Thermobifida fusca
0.00	2	2	0	269800	Thermobifida fusca YX
0.00	17	0	0	85010	Pseudonocardineae
0.00	17	1	F	2070	Pseudonocardiaceae
0.00	7	0	G	1847	Pseudonocardia
0.00	7	0	S	240495	Pseudonocardia dioxanivorans
0.00	7	7	0	675635	Pseudonocardia dioxanivorans CB1190
0.00	4	0	G	1813	Amycolatopsis
0.00	2	0	S	31958	Amycolatopsis orientalis
0.00	2	2	0	1156913	Amycolatopsis orientalis HCCB10007
0.00	2	2	S	33910	Amycolatopsis mediterranei
0.00	3	0	G	1835	Saccharopolyspora
0.00	3	0	S	1836	Saccharopolyspora erythraea
0.00	3	3	0	405948	Saccharopolyspora erythraea NRRL 2338
0.00	1	0	G	40566	Actinosynnema
0.00	1	0	S	40567	Actinosynnema mirum
0.00	1	1	0	446462	Actinosynnema mirum DSM 43827
0.00	1	0	G	147067	Thermobispora
0.00	1	0	S	2006	Thermobispora bispora
0.00	1	1	0	469371	Thermobispora bispora DSM 43833
0.00	14	2	0	85013	Frankineae
0.00	8	1	F	85030	Geodermatophilaceae
0.00	4	0	G	38501	Blastococcus
0.00	4	0	S	138336	Blastococcus saxobsidens
0.00	4	4	0	1146883	Blastococcus saxobsidens DD2
0.00	3	0	G	88138	Modestobacter
0.00	3	3	S	477641	Modestobacter marinus
0.00	3	0	F	74712	Frankiaceae
0.00	3	0	G	1854	Frankia
0.00	2	2	S	656024	Frankia symbiont of Datisca glomerata
0.00	1	1	S	298654	Frankia sp. EuI1c
0.00	1	0	F	85031	Nakamurellaceae
0.00	1	0	G	53460	Nakamurella
0.00	1	0	S	53461	Nakamurella multipartita
0.00	1	1	0	479431	Nakamurella multipartita DSM 44233

0.00	8	0	0	85008	Micromonosporineae
0.00	8	2	F	28056	Micromonosporaceae
0.00	3	0	G	1865	Actinoplanes
0.00	2	0	S	1866	Actinoplanes missouriensis
0.00	2	2	0	512565	Actinoplanes missouriensis 431
0.00	1	0	S	196914	Actinoplanes friuliensis
0.00	1	1	0	1246995	Actinoplanes friuliensis DSM 7358
0.00	1	1	G	1873	Micromonospora
0.00	1	0	G	84593	Verrucosispora
0.00	1	0	S	1003110	Verrucosispora maris
0.00	1	1	0	263358	Verrucosispora maris AB-18-032
0.00	1	0	G	168694	Salinispora
0.00	1	0	S	168697	Salinispora arenicola
0.00	1	1	0	391037	Salinispora arenicola CNS-205
0.00	2	0	0	414714	Catenulisporineae
0.00	2	0	F	414877	Catenulisporaceae
0.00	2	0	G	414878	Catenulispora
0.00	2	0	S	304895	Catenulispora acidiphila
0.00	2	2	0	479433	Catenulispora acidiphila DSM 44928
0.00	1	0	0	85014	Glycomycineae
0.00	1	0	F	85034	Glycomycetaceae
0.00	1	0	G	283810	Stackebrandtia
0.00	1	0	S	283811	Stackebrandtia nassauensis
0.00	1	1	0	446470	Stackebrandtia nassauensis DSM 44728
0.00	4	0	O	85004	Bifidobacteriales
0.00	4	0	F	31953	Bifidobacteriaceae
0.00	3	0	G	2701	Gardnerella
0.00	3	1	S	2702	Gardnerella vaginalis
0.00	1	1	0	525284	Gardnerella vaginalis ATCC 14019
0.00	1	1	0	553190	Gardnerella vaginalis 409-05
0.00	1	0	G	1678	Bifidobacterium
0.00	1	0	S	1685	Bifidobacterium breve
0.00	1	1	0	866777	Bifidobacterium breve ACS-071-V-Sch8b
0.00	6	0	0	84998	Coriobacteridae
0.00	6	0	O	84999	Coriobacteriales
0.00	6	0	0	255727	Coriobacterineae
0.00	6	0	F	84107	Coriobacteriaceae
0.00	6	0	G	1380	Atopobium
0.00	6	0	S	1382	Atopobium parvulum
0.00	6	6	0	521095	Atopobium parvulum DSM 20469
0.00	1	0	0	84995	Rubrobacteridae
0.00	1	0	O	588673	Solirubrobacterales
0.00	1	0	F	320583	Conexibacteraceae
0.00	1	0	G	191494	Conexibacter
0.00	1	0	S	191495	Conexibacter woesei
0.00	1	1	0	469383	Conexibacter woesei DSM 14684
0.00	358	0	P	203691	Spirochaetes
0.00	358	0	C	203692	Spirochaetia
0.00	358	0	O	136	Spirochaetales
0.00	245	0	F	137	Spirochaetaceae
0.00	122	0	G	138	Borrelia
0.00	51	0	S	29520	Borrelia crocidurae
0.00	51	51	0	1155096	Borrelia crocidurae str. Achema
0.00	44	0	0	64895	Borrelia burgdorferi group
0.00	42	0	S	62088	Borrelia valaisiana
0.00	42	42	0	445987	Borrelia valaisiana VS116
0.00	2	2	S	139	Borrelia burgdorferi
0.00	20	0	S	44449	Borrelia recurrentis
0.00	20	20	0	412418	Borrelia recurrentis A1
0.00	4	0	S	142	Borrelia turicatae
0.00	4	4	0	314724	Borrelia turicatae 91E135
0.00	3	0	S	47466	Borrelia miyamotoi
0.00	3	3	0	1302858	Borrelia miyamotoi LB-2001
0.00	122	0	G	399320	Sphaerochaeta
0.00	122	0	S	273376	Sphaerochaeta coccoides
0.00	122	122	0	760011	Sphaerochaeta coccoides DSM 17374
0.00	1	0	G	157	Treponema
0.00	1	0	S	88058	Treponema primitia
0.00	1	1	0	545694	Treponema primitia ZAS-2
0.00	60	0	F	143786	Brachyspiraceae
0.00	60	0	G	29521	Brachyspira
0.00	60	58	S	52584	Brachyspira pilosicoli
0.00	1	1	0	1042417	Brachyspira pilosicoli P43/6/78
0.00	1	1	0	1161918	Brachyspira pilosicoli WesB
0.00	53	0	F	170	Leptospiraceae
0.00	53	0	G	171	Leptospira
0.00	44	0	S	173	Leptospira interrogans
0.00	44	0	0	44275	Leptospira interrogans serovar Copenhageni
0.00	44	44	0	267671	Leptospira interrogans serovar Copenhageni str. Fiocruz L1-130
0.00	9	0	S	172	Leptospira biflexa
0.00	9	9	0	145259	Leptospira biflexa serovar Patoc
0.00	168	0	P	32066	Fusobacteria
0.00	168	0	C	203490	Fusobacteriia

0.00	168	0	O	203491	Fusobacteriales
0.00	111	0	F	1129771	Leptotrichiaceae
0.00	109	0	G	32067	Leptotrichia
0.00	109	0	S	40542	Leptotrichia buccalis
0.00	109	109	0	523794	Leptotrichia buccalis C-1013-b
0.00	2	0	G	34104	Streptobacillus
0.00	2	0	S	34105	Streptobacillus moniliformis
0.00	2	2	0	519441	Streptobacillus moniliformis DSM 12112
0.00	57	0	F	203492	Fusobacteriaceae
0.00	57	0	G	848	Fusobacterium
0.00	57	0	S	851	Fusobacterium nucleatum
0.00	57	0	0	76859	Fusobacterium nucleatum subsp. animalis
0.00	57	57	0	469607	Fusobacterium nucleatum subsp. animalis 4_8
0.00	85	0	P	200940	Thermodesulfobacteria
0.00	85	0	C	67799	Thermodesulfobacteria
0.00	85	0	O	188710	Thermodesulfobacteriales
0.00	85	0	F	188711	Thermodesulfobacteriaceae
0.00	85	0	G	1740	Thermodesulfobacterium
0.00	85	0	S	1295609	Thermodesulfobacterium geofontis
0.00	85	85	0	795359	Thermodesulfobacterium geofontis OPF15
0.00	72	0	0	51290	Chlamydiae/Verrucomicrobia group
0.00	52	0	P	204428	Chlamydiae
0.00	52	0	C	204429	Chlamydiia
0.00	52	0	O	51291	Chlamydiales
0.00	42	0	F	92713	Parachlamydiaceae
0.00	41	0	G	83551	Parachlamydia
0.00	41	0	S	83552	Parachlamydia acanthamoebae
0.00	41	41	0	765952	Parachlamydia acanthamoebae UV-7
0.00	1	0	G	282132	Candidatus Protochlamydia
0.00	1	0	S	362787	Candidatus Protochlamydia amoebophila
0.00	1	1	0	264201	Candidatus Protochlamydia amoebophila UWE25
0.00	10	0	F	809	Chlamydiaceae
0.00	10	0	0	1113537	Chlamydia/Chlamydophila group
0.00	9	0	G	810	Chlamydia
0.00	9	1	S	85991	Chlamydia pecorum
0.00	8	8	0	1234367	Chlamydia pecorum PV3056/3
0.00	1	0	G	83553	Chlamydophila
0.00	1	0	S	83556	Chlamydophila felis
0.00	1	1	0	264202	Chlamydophila felis Fe/C-56
0.00	20	0	P	74201	Verrucomicrobia
0.00	20	0	C	414999	Opitutae
0.00	11	0	0	415000	Opitutales
0.00	11	0	F	134623	Opitutaceae
0.00	11	0	G	178440	Opitutus
0.00	11	0	S	107709	Opitutus terrae
0.00	11	11	0	452637	Opitutus terrae PB90-1
0.00	9	0	O	415001	Puniceicoccales
0.00	9	0	F	415002	Puniceicoccaceae
0.00	9	0	G	442430	Coraliomargarita
0.00	9	0	S	395922	Coraliomargarita akajimensis
0.00	9	9	0	583355	Coraliomargarita akajimensis DSM 45221
0.00	32	0	P	200918	Thermotogae
0.00	32	0	C	188708	Thermotogae
0.00	32	0	O	2419	Thermotogales
0.00	32	0	F	188709	Thermotogaceae
0.00	24	0	G	1184396	Mesotoga
0.00	24	0	S	1184387	Mesotoga prima
0.00	24	24	0	660470	Mesotoga prima MesG1.Ag.4.2
0.00	7	0	G	2422	Fervidobacterium
0.00	7	0	S	93466	Fervidobacterium pennivorans
0.00	7	7	0	771875	Fervidobacterium pennivorans DSM 9078
0.00	1	0	G	160798	Marinitoga
0.00	1	0	S	149715	Marinitoga piezophila
0.00	1	1	0	443254	Marinitoga piezophila KA3
0.00	6	0	P	1297	Deinococcus-Thermus
0.00	6	0	C	188787	Deinococci
0.00	4	0	O	118964	Deinococcales
0.00	4	0	F	183710	Deinococcaceae
0.00	4	1	G	1298	Deinococcus
0.00	1	0	S	309887	Deinococcus maricopensis
0.00	1	1	0	709986	Deinococcus maricopensis DSM 21211
0.00	1	0	S	310783	Deinococcus deserti
0.00	1	1	0	546414	Deinococcus deserti VCD115
0.00	1	0	S	502394	Deinococcus gobiensis
0.00	1	1	0	745776	Deinococcus gobiensis I-0
0.00	2	0	O	68933	Thermales
0.00	2	0	F	188786	Thermaceae
0.00	2	0	G	270	Thermus
0.00	2	1	S	274	Thermus thermophilus
0.00	1	1	0	798128	Thermus thermophilus JL-18
0.00	2	0	P	200795	Chloroflexi
0.00	1	0	C	32061	Chloroflexia
0.00	1	0	O	32064	Chloroflexales

0.00	1	0	0	1508595	Roseiflexineae
0.00	1	0	F	1508635	Roseiflexaceae
0.00	1	0	G	120961	Roseiflexus
0.00	1	0	S	120962	Roseiflexus castenholzii
0.00	1	1	0	383372	Roseiflexus castenholzii DSM 13941
0.00	1	0	C	189775	Thermomicrobia
0.00	1	0	0	85000	Sphaerobacteridae
0.00	1	0	O	85001	Sphaerobacterales
0.00	1	0	0	255728	Sphaerobacterineae
0.00	1	0	F	85002	Sphaerobacteraceae
0.00	1	0	G	2056	Sphaerobacter
0.00	1	0	S	2057	Sphaerobacter thermophilus
0.00	1	1	0	479434	Sphaerobacter thermophilus DSM 20745
0.01	11953	613	D	2157	Archaea
0.01	11302	249	P	28890	Euryarchaeota
0.00	5978	0	C	183939	Methanococci
0.00	5978	0	O	2182	Methanococcales
0.00	5978	0	F	2183	Methanococcaceae
0.00	5966	58	G	2184	Methanococcus
0.00	5541	0	S	2188	Methanococcus voltae
0.00	5541	5541	0	456320	Methanococcus voltae A3
0.00	356	0	S	42879	Methanococcus aeolicus
0.00	356	356	0	419665	Methanococcus aeolicus Nankai-3
0.00	11	0	S	2187	Methanococcus vannielii
0.00	11	11	0	406327	Methanococcus vannielii SB
0.00	12	0	G	155862	Methanothermococcus
0.00	12	0	S	155863	Methanothermococcus okinawensis
0.00	12	12	0	647113	Methanothermococcus okinawensis IH1
0.00	4445	0	C	183963	Halobacteria
0.00	4445	0	O	2235	Halobacteriales
0.00	4445	7	F	2236	Halobacteriaceae
0.00	4205	0	G	293431	Haloquadratum
0.00	4205	0	S	293091	Haloquadratum walsbyi
0.00	4205	4205	0	362976	Haloquadratum walsbyi DSM 16790
0.00	142	0	G	332951	Halovivax
0.00	142	0	S	387341	Halovivax ruber
0.00	142	142	0	797302	Halovivax ruber XH-70
0.00	37	0	G	63742	Natrialba
0.00	37	0	S	13769	Natrialba magadii
0.00	37	37	0	547559	Natrialba magadii ATCC 43099
0.00	22	0	G	203135	Halomicrobium
0.00	22	0	S	57705	Halomicrobium mukohataei
0.00	22	22	0	485914	Halomicrobium mukohataei DSM 12286
0.00	19	0	G	1269201	Salinarchaeum
0.00	19	19	S	1333523	Salinarchaeum sp. Harcht-Bsk1
0.00	10	0	G	332246	Halalkalicoccus
0.00	10	0	S	413810	Halalkalicoccus jeotgali
0.00	10	10	0	795797	Halalkalicoccus jeotgali B3
0.00	3	0	G	29287	Natronococcus
0.00	3	0	S	29288	Natronococcus occultus
0.00	3	3	0	694430	Natronococcus occultus SP4
0.00	602	0	C	224756	Methanomicrobia
0.00	563	0	O	94695	Methanosarcinales
0.00	563	0	F	2206	Methanosarcinaceae
0.00	466	0	G	2207	Methanosarcina
0.00	327	0	S	2209	Methanosarcina mazei
0.00	319	319	0	192952	Methanosarcina mazei Go1
0.00	8	8	0	1236903	Methanosarcina mazei Tuc01
0.00	112	0	S	2208	Methanosarcina barkeri
0.00	112	112	0	269797	Methanosarcina barkeri str. Fusaro
0.00	27	0	S	2214	Methanosarcina acetivorans
0.00	27	27	0	188937	Methanosarcina acetivorans C2A
0.00	39	0	G	2175	Methanohalophilus
0.00	39	0	S	2176	Methanohalophilus mahii
0.00	39	39	0	547558	Methanohalophilus mahii DSM 5219
0.00	37	0	G	101191	Methanomethylovorans
0.00	37	0	S	101192	Methanomethylovorans hollandica
0.00	37	37	0	867904	Methanomethylovorans hollandica DSM 15978
0.00	15	0	G	196136	Methanosalsum
0.00	15	0	S	39669	Methanosalsum zhilinae
0.00	15	15	0	679901	Methanosalsum zhilinae DSM 4017
0.00	6	0	G	2225	Methanococcoides
0.00	6	0	S	29291	Methanococcoides burtonii
0.00	6	6	0	259564	Methanococcoides burtonii DSM 6242
0.00	39	0	O	2191	Methanomicrobiales
0.00	30	0	F	88404	Methanocorpusculaceae
0.00	30	0	G	2192	Methanocorpusculum
0.00	30	0	S	83984	Methanocorpusculum labreanum
0.00	30	30	0	410358	Methanocorpusculum labreanum Z
0.00	5	0	F	2194	Methanomicrobiaceae
0.00	5	0	G	2314	Methanoplanus
0.00	5	0	S	54120	Methanoplanus petrolearius
0.00	5	5	0	679926	Methanoplanus petrolearius DSM 11571

0.00	4	0	F	196137	Methanospirillaceae
0.00	4	0	G	2202	Methanospirillum
0.00	4	0	S	2203	Methanospirillum hungatei
0.00	4	4	0	323259	Methanospirillum hungatei JF-1
0.00	25	0	C	183925	Methanobacteria
0.00	25	0	O	2158	Methanobacteriales
0.00	25	0	F	2159	Methanobacteriaceae
0.00	25	0	G	2172	Methanobrevibacter
0.00	24	24	S	224719	Methanobrevibacter sp. AbM4
0.00	1	0	S	2173	Methanobrevibacter smithii
0.00	1	1	0	420247	Methanobrevibacter smithii ATCC 35061
0.00	3	0	C	183968	Thermococci
0.00	3	0	O	2258	Thermococcales
0.00	3	0	F	2259	Thermococcaceae
0.00	3	0	G	2260	Pyrococcus
0.00	3	3	S	342949	Pyrococcus sp. NA2
0.00	38	0	P	28889	Crenarchaeota
0.00	38	0	C	183924	Thermoprotei
0.00	38	0	O	114380	Desulfurococcales
0.00	38	0	F	2307	Pyrodictiaceae
0.00	38	0	G	54251	Pyrolobus
0.00	38	0	S	54252	Pyrolobus fumarii
0.00	38	38	0	694429	Pyrolobus fumarii 1A
0.03	38569	478	D	10239	Viruses
0.03	36293	1031	0	35237	dsDNA viruses, no RNA stage
0.01	15158	256	0	51368	unclassified dsDNA viruses
0.01	7612	7612	S	1349410	Pandoravirus salinus
0.01	7290	7290	S	1349409	Pandoravirus dulcis
0.01	12324	6	O	28883	Caudovirales
0.01	11991	0	F	10744	Podoviridae
0.01	11948	0	0	542835	Autographivirinae
0.01	11948	0	G	110456	T7likevirus
0.01	11948	0	0	329157	unclassified T7-like viruses
0.01	11948	11948	S	227720	Yersinia phage phiA1122
0.00	43	0	0	196895	unclassified Podoviridae
0.00	43	43	S	941970	Liberibacter phage SC2
0.00	222	0	F	10662	Myoviridae
0.00	154	0	0	857473	Spounavirinae
0.00	120	0	G	140409	Spounalikevirus
0.00	117	117	S	10685	Bacillus phage SPO1
0.00	3	0	0	365007	unclassified SPO1-like viruses
0.00	3	3	S	1007127	Staphylococcus phage Sb-1
0.00	34	0	0	857474	unclassified Spounavirinae
0.00	34	34	S	857312	Brochothrix phage A9
0.00	66	0	0	196896	unclassified Myoviridae
0.00	35	35	S	1477406	Dickeya phage RC-2014
0.00	31	31	S	1190451	Campylobacter phage CP21
0.00	1	0	0	857479	Peduovirinae
0.00	1	0	G	140410	P2likevirus
0.00	1	0	0	329161	unclassified P2-like viruses
0.00	1	1	S	1530085	Ralstonia phage RSY1
0.00	1	0	0	1198136	Tevenvirinae
0.00	1	0	G	10663	T4likevirus
0.00	1	0	S	348604	Enterobacteria phage T4 sensu lato
0.00	1	1	0	10665	Enterobacteria phage T4
0.00	105	0	F	10699	Siphoviridae
0.00	105	2	0	196894	unclassified Siphoviridae
0.00	50	50	S	73422	Streptococcus phage TP-J34
0.00	48	48	S	633135	Streptococcus phage Abc2
0.00	2	2	S	1229784	Propionibacterium phage P14.4
0.00	1	1	S	1147042	Staphylococcus phage StB12
0.00	1	1	S	1229751	Lactococcus phage BM13
0.00	1	1	S	1229792	Propionibacterium phage P1.1
0.00	3675	4	O	548681	Herpesvirales
0.00	2693	2	F	548682	Alloherpesviridae
0.00	2015	0	G	172653	Ictalurivirus
0.00	2015	2015	S	10401	Ictalurid herpesvirus 1
0.00	676	96	G	692606	Cyprinivirus
0.00	451	451	S	317858	Cyprinid herpesvirus 1
0.00	94	94	S	180230	Cyprinid herpesvirus 3
0.00	35	35	S	317878	Cyprinid herpesvirus 2
0.00	978	0	F	10292	Herpesviridae
0.00	647	0	0	10357	Betaherpesvirinae
0.00	598	0	G	10358	Cytomegalovirus
0.00	579	579	S	1535247	Saimiriine herpesvirus 4
0.00	13	13	S	50290	Aotine herpesvirus 1
0.00	6	0	0	342596	unclassified Cytomegalovirus
0.00	6	6	S	944969	Cynomolgus macaque cytomegalovirus strain Ottawa
0.00	31	0	G	548689	Proboscivirus
0.00	31	31	S	146015	Elephantid herpesvirus 1
0.00	17	0	G	40272	Roseolovirus
0.00	17	17	S	32603	Human herpesvirus 6A
0.00	1	0	0	686603	unassigned Betaherpesvirinae

0.00	1	1	S	33706	Caviid herpesvirus 2
0.00	185	0	0	10374	Gammaherpesvirinae
0.00	166	0	G	548687	Macavirus
0.00	89	89	S	35252	Alcelaphine herpesvirus 1
0.00	44	44	S	138184	Alcelaphine herpesvirus 2
0.00	33	33	S	10398	Ovine herpesvirus 2
0.00	11	0	G	10379	Rhadinovirus
0.00	11	11	S	85618	Ateline herpesvirus 3
0.00	8	0	G	548688	Percavirus
0.00	8	8	S	12657	Equid herpesvirus 2
0.00	146	0	0	10293	Alphaherpesvirinae
0.00	99	0	G	10319	Varicellovirus
0.00	99	99	S	35244	Bovine herpesvirus 5
0.00	47	0	G	10294	Simplexvirus
0.00	36	36	S	10310	Human herpesvirus 2
0.00	11	11	S	10317	Cercopithecine herpesvirus 2
0.00	1468	0	F	10482	Polydnaviridae
0.00	1075	0	G	10483	Ichnovirus
0.00	730	730	S	419435	Glypta fumiferanae ichnovirus
0.00	345	345	S	265522	Hyposoter fugitivus ichnovirus
0.00	393	0	G	10485	Bracovirus
0.00	393	393	S	39640	Cotesia congregata bracovirus
0.00	751	0	F	10442	Baculoviridae
0.00	729	0	G	558016	Alphabaculovirus
0.00	367	332	S	51313	Helicoverpa armigera nucleopolyhedrovirus
0.00	35	35	0	566972	Helicoverpa armigera NPV NNg1
0.00	83	83	S	320432	Chrysodeixis chalcites nucleopolyhedrovirus
0.00	76	76	S	208013	Agrotis ipsilon multiple nucleopolyhedrovirus
0.00	63	63	S	1307954	Maruca vitrata nucleopolyhedrovirus
0.00	50	0	0	328432	unclassified Nucleopolyhedrovirus
0.00	37	37	S	490711	Orgyia leucostigma NPV
0.00	13	13	S	307461	Apocheima cinerarium nucleopolyhedrovirus
0.00	35	35	S	207830	Mamestra configurata nucleopolyhedrovirus A
0.00	17	17	S	31508	Agrotis segetum nucleopolyhedrovirus
0.00	16	0	0	745176	unclassified Alphabaculovirus
0.00	16	16	S	1367205	Choristoneura rosaceana alphabaculovirus
0.00	12	12	S	74320	Buzura suppressaria nucleopolyhedrovirus
0.00	10	10	S	307467	Euproctis pseudoconspersa nucleopolyhedrovirus
0.00	22	0	G	558017	Betabaculovirus
0.00	21	0	0	342107	unclassified Betabaculovirus
0.00	21	21	S	166056	Epinotia aporema granulovirus
0.00	1	1	S	51677	Xestia c-nigrum granulovirus
0.00	604	0	F	10240	Poxviridae
0.00	338	0	0	10241	Chordopoxvirinae
0.00	311	0	G	10282	Yatapoxvirus
0.00	311	311	S	38804	Yaba monkey tumor virus
0.00	27	0	G	10242	Orthopoxvirus
0.00	19	19	S	12643	Ectromelia virus
0.00	8	8	S	10243	Cowpox virus
0.00	266	0	0	10284	Entomopoxvirinae
0.00	250	0	G	10286	Betaentomopoxvirus
0.00	231	231	S	28321	Amsacta moorei entomopoxvirus 'L'
0.00	19	0	0	1311761	unclassified Betaentomopoxvirus
0.00	19	19	S	1293539	Choristoneura rosaceana entomopoxvirus 'L'
0.00	16	0	G	62098	Alphaentomopoxvirus
0.00	16	16	S	62099	Anomala cuprea entomopoxvirus
0.00	493	0	F	10501	Phycodnaviridae
0.00	185	0	G	181085	Phaeovirus
0.00	185	185	S	37665	Ectocarpus siliculosus virus 1
0.00	164	0	G	181086	Prymnesiovirus
0.00	164	0	0	358403	unclassified Prymnesiovirus
0.00	164	164	S	251749	Phaeocystis globosa virus
0.00	116	0	G	181083	Chlorovirus
0.00	116	116	S	46021	Paramecium bursaria Chlorella virus NY2A
0.00	28	0	0	455363	unclassified Phycodnaviridae
0.00	28	28	S	1474867	Aureococcus anophagefferens virus
0.00	466	0	F	549779	Mimiviridae
0.00	434	397	0	985780	unclassified Mimiviridae
0.00	18	0	S	985782	Moumouvirus
0.00	18	18	0	1269028	Acanthamoeba polyphaga moumouvirus
0.00	11	11	S	1235314	Megavirus lba
0.00	8	8	S	1094892	Megavirus chiliensis
0.00	32	0	G	1513234	Cafeteriavirus
0.00	32	0	S	1513235	Cafeteria roenbergensis virus
0.00	32	32	0	693272	Cafeteria roenbergensis virus BV-PW1
0.00	129	0	F	43682	Ascoviridae
0.00	129	0	G	43680	Ascovirus
0.00	129	0	0	328613	unclassified Ascovirus
0.00	129	129	S	328615	Trichoplusia ni ascovirus 2c
0.00	102	0	F	1511852	Nudiviridae
0.00	91	0	0	1110703	unclassified Nudiviridae
0.00	91	91	S	1529056	Penaeus monodon nudivirus
0.00	11	0	0	1511853	Alphanudivirus

0.00	11	11	S	92521	Oryctes rhinoceros nudivirus
0.00	33	0	O	1511857	Ligamenvirales
0.00	33	0	F	10477	Lipothrixviridae
0.00	33	0	G	564646	Deltalipothrixvirus
0.00	33	33	S	300186	Acidianus filamentous virus 2
0.00	21	0	F	196937	Nimaviridae
0.00	21	0	G	249585	Whispovirus
0.00	21	0	S	342409	White spot syndrome virus
0.00	21	21	0	92652	Shrimp white spot syndrome virus
0.00	20	20	0	79205	unclassified dsDNA phages
0.00	16	0	F	10508	Adenoviridae
0.00	16	0	G	10552	Aviadenovirus
0.00	16	16	S	190065	Fowl aviadenovirus E
0.00	1	0	F	10486	Iridoviridae
0.00	1	0	G	10491	Chloriridovirus
0.00	1	1	S	345201	Invertebrate iridescent virus 3
0.00	1	0	F	944644	Marseilleviridae
0.00	1	0	0	1513460	unassigned Marseilleviridae
0.00	1	1	S	999883	Lausannevirus
0.00	1492	0	0	439488	ssRNA viruses
0.00	1468	0	0	35278	ssRNA positive-strand viruses, no DNA stage
0.00	717	0	F	39740	Bromoviridae
0.00	717	0	G	12300	Bromovirus
0.00	717	717	S	188141	Spring beauty latent virus
0.00	338	0	O	464095	Picornavirales
0.00	220	0	F	12058	Picornaviridae
0.00	209	0	G	688449	Salivirus
0.00	209	0	0	1330992	unclassified Salivirus
0.00	209	209	S	1547495	Salivirus FHB
0.00	11	0	G	1330065	Aquamavirus
0.00	11	0	S	1330066	Aquamavirus A
0.00	11	11	0	471728	Seal picornavirus type 1
0.00	118	0	0	675074	unclassified Picornavirales
0.00	118	118	S	1439369	Carp picornavirus 1
0.00	256	0	F	39733	Astroviridae
0.00	256	0	0	352926	unclassified Astroviridae
0.00	256	256	S	1074210	Mouse astrovirus M-52/USA/2008
0.00	78	0	0	38173	unclassified ssRNA positive-strand viruses
0.00	57	57	S	1491393	Jingmen tick virus
0.00	21	21	S	631345	Solenopsis invicta virus 3
0.00	59	0	O	76804	Nidovirales
0.00	59	0	F	11118	Coronaviridae
0.00	59	0	0	693995	Coronavirinae
0.00	59	59	G	693996	Alphacoronavirus
0.00	8	0	F	11974	Caliciviridae
0.00	8	0	0	179239	unclassified Caliciviridae
0.00	8	0	S	646294	St-Valerien swine virus
0.00	8	8	0	520973	Calicivirus pig/AB90/CAN
0.00	5	0	F	11050	Flaviviridae
0.00	4	0	G	11102	Hepacivirus
0.00	4	0	S	11103	Hepatitis C virus
0.00	4	4	0	40271	Hepatitis C virus genotype 2
0.00	1	0	0	38144	unclassified Flaviviridae
0.00	1	1	S	39113	Hepatitis GB virus B
0.00	5	0	F	39729	Potyviridae
0.00	5	0	G	12195	Potyvirus
0.00	5	5	S	167129	Moroccan watermelon mosaic virus
0.00	2	0	O	675063	Tymovirales
0.00	2	0	F	249184	Tymoviridae
0.00	2	0	G	12051	Marafivirus
0.00	2	2	S	630199	Grapevine Syrah virus 1
0.00	24	0	0	35301	ssRNA negative-strand viruses
0.00	19	0	F	11571	Bunyaviridae
0.00	19	0	G	11611	Tospovirus
0.00	19	0	0	326176	unclassified Tospovirus
0.00	18	18	S	460926	Tomato zonate spot virus
0.00	1	1	S	89471	Melon yellow spot virus
0.00	5	0	F	11308	Orthomyxoviridae
0.00	5	0	G	324913	Isavirus
0.00	5	5	S	55987	Infectious salmon anemia virus
0.00	268	0	0	35325	dsRNA viruses
0.00	190	0	F	11012	Partitiviridae
0.00	150	0	G	1511809	Betapartitivirus
0.00	97	97	S	1323524	Red clover cryptic virus 2
0.00	32	32	S	1323525	White clover cryptic virus 2
0.00	12	0	S	1511840	Primula malacoides virus 1
0.00	12	12	0	479713	Primula malacoides virus China/Mar2007
0.00	4	4	S	674983	Pleurotus ostreatus virus 1
0.00	4	4	S	1323529	Dill cryptic virus 2
0.00	1	1	S	1323523	Hop trefoil cryptic virus 2
0.00	33	0	0	37960	unclassified Partitiviridae
0.00	33	33	S	1411681	Rhizoctonia solani dsRNA virus 2
0.00	7	0	G	1511808	Alphapartitivirus

0.00	7	7	S	54289	Vicia cryptic virus
0.00	56	0	F	249310	Chrysoviridae
0.00	56	0	G	11014	Chrysovirus
0.00	56	56	S	158372	Penicillium chrysogenum virus
0.00	22	0	F	10880	Reoviridae
0.00	22	0	0	36446	unclassified Reoviridae
0.00	22	22	S	907191	Raspberry latent virus
0.00	19	0	0	686617	unassigned viruses
0.00	19	0	F	1285590	Hytrosaviridae
0.00	15	0	G	1285593	Muscavirus
0.00	15	0	S	1285595	Musca hytrovirus
0.00	15	15	0	523909	Musca domestica salivary gland hypertrophy virus
0.00	4	0	G	1285591	Glossinavirus
0.00	4	0	S	1285594	Glossina hytrovirus
0.00	4	4	0	379529	Glossina pallidipes salivary gland hypertrophy virus
0.00	10	0	0	12429	unclassified viruses
0.00	10	10	S	1523023	Gentian ovary ring-spot virus
0.00	7	0	0	12877	Satellites
0.00	7	0	0	198601	Satellite Nucleic Acids
0.00	7	0	0	361688	Single stranded DNA satellites
0.00	7	0	0	190729	Betasatellites
0.00	7	0	0	361689	unclassified Begomovirus-associated DNA beta-like sequences
0.00	7	7	S	1367671	Malvastrum leaf curl Philippines betasatellite
0.00	2	0	0	35268	Retro-transcribing viruses
0.00	2	0	F	11632	Retroviridae
0.00	2	0	0	35276	unclassified Retroviridae
0.00	2	0	0	206037	Human endogenous retroviruses
0.00	2	0	S	45617	Human endogenous retrovirus K
0.00	2	2	0	166122	Human endogenous retrovirus K113
