# Supplementary material for: Genomes of the Bacterial Endosymbionts of Carrot Psyllid Trioza apicalis Suggest Complementary Biosynthetic Capabilities
Source: Curr Microbiol. 2025 Feb 20;82(4):145. doi: 10.1007/s00284-025-04119-y (PMC11842425; doi:10.1007/s00284-025-04119-y)
Supplement: Supplementary file 3 — Supplementary file3 (PDF 1069 kb) [file 284_2025_4119_MOESM3_ESM.pdf]

Genomes of the bacterial endosymbionts of carrot psyllid *Trioza apicalis* suggest complementary biosynthetic capabilities

Current Microbiology

Sarah Thompson, Jinhui Wang, Thomas Schott, Riitta Nissinen, Minna Haapalainen

University of Helsinki, email: minna.haapalainen@helsinki.fi; minna.haapalainen@luke.fi

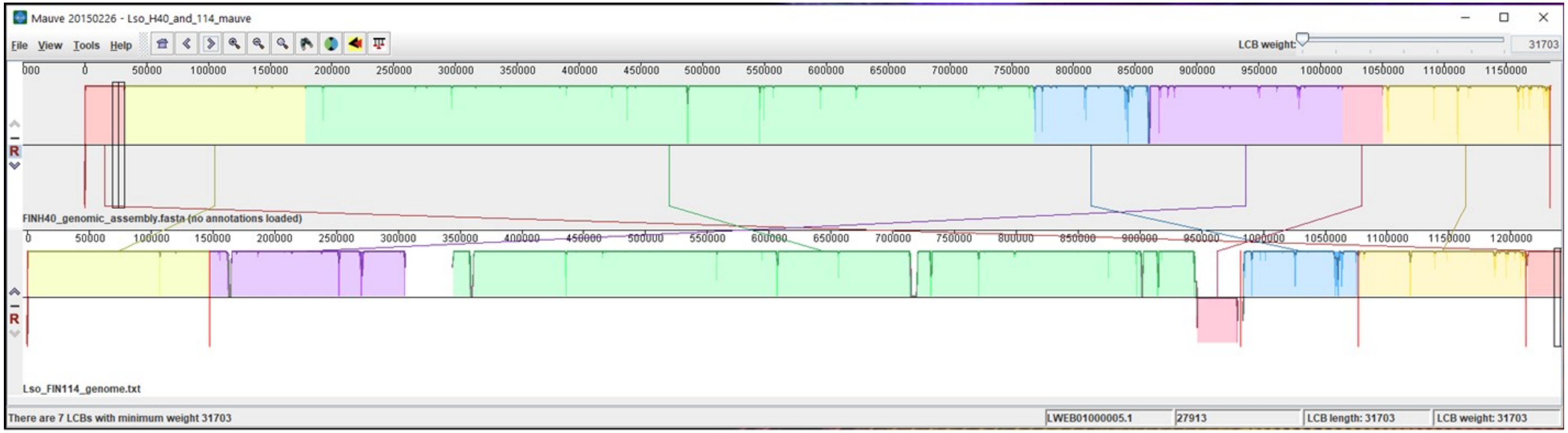

Supplementary Data S3. Genomic alignment by Mauve of the '*Candidatus* Liberibacter solanacearum' haplotype C assemblies FINH40 and FIN114 (NZ_ LWEB00000000.1).
