# Supplementary material for: Genomes of the Bacterial Endosymbionts of Carrot Psyllid Trioza apicalis Suggest Complementary Biosynthetic Capabilities
Source: Curr Microbiol. 2025 Feb 20;82(4):145. doi: 10.1007/s00284-025-04119-y (PMC11842425; doi:10.1007/s00284-025-04119-y)
Supplement: Supplementary file 4 — Supplementary file4 (PDF 898 kb) [file 284_2025_4119_MOESM4_ESM.pdf]

Genomes of the bacterial endosymbionts of carrot psyllid *Trioza apicalis* suggest complementary biosynthetic capabilities

Current Microbiology

Sarah Thompson, Jinhui Wang, Thomas Schott, Riitta Nissinen, Minna Haapalainen

University of Helsinki, email: minna.haapalainen@helsinki.fi; minna.haapalainen@luke.fi

Supplementary Data S4. Complementarity of the biosynthetic capacities encoded by the genomes of '*Candidatus* Carsonella ruddii' and gamma proteobacterium endosymbiont of *Trioza apicalis*. The proteins encoded by each genome are indicated and EC numbers are shown for enzymes.

**Functional category**	**Protein/ pathway**	**'*Candidatus* Carsonella ruddii'**	**Gamma proteobacterium endosymbiont**
Energy metabolism	F0F1 ATP synthase	F0 subunits A, B, C<br>F1 subunits α, β, γ	F0 subunits A, B, C<br>F1 subunits α, β, γ, δ, ε
	Cytochrome o	ubiquinol oxidase cyoABCD	ubiquinol oxidase cyoABCD
	Glykolysis and pentose phosphate pathway	6-phosphogluconate dehydrogenase (gnd)	Glyceraldehyde-3-phosphate dehydrogenase A (gapA, EC: 1.2.1.12), pyruvate kinase (EC:2.7.1.40), 6-phosphogluconate dehydrogenase (gnd, EC:1.1.1.44)
	TCA cycle	absent	succinate dehydrogenase (sdhA, sdhB, sdhD, EC:1.3.5.1), 2-oxoglutarate dehydrogenase (sucA EC:1.2.4.2, sucB EC:2.3.1.61), succinyl-CoA synthetase (sucC, sucD, EC:6.2.1.5), succinate-CoA ligase (α, β) (EC: 6.2.1.4), malate: quinone dehydrogenase (mqo, EC:1.1.5.4)
Nucleotide biosynthesis		ribose-phosphate pyrophosphokinase (PRPP, EC: 2.7.6.1)	carA and carB [EC:6.3.5.5], pyrB [EC:2.1.3.2], pyrC [EC:3.5.2.3], pyrD [EC:1.3.5.2], pyrF [EC:4.1.1.23], CTP synthase pyrG [EC 6.3.4.2], thyA[EC: 2.1.1.45], deoD [EC: 2.4.2.1], ribose-phosphate pyrophosphokinase (PRPP, EC: 2.7.6.1)
DNA replication and repair	Replication initiation	DNA primase	DNA primase, replication initiation protein
	DNA helicase	Replicative DNA helicase (DnaB)	Replicative DNA helicase (DnaB), DNA helicase (UvrD)
	DNA polymerase III	subunits α[EC:2.7.7.7] and ε[EC:2.7.7.7]	subunits α[EC:2.7.7.7], β[EC:2.7.7.7], γ/τ[EC:2.7.7.7], δ[EC:2.7.7.7], δ' [EC:2.7.7.7], ε[EC:2.7.7.7]
	DNA-binding protein	absent	single-stranded DNA-binding protein (Ssp1)
	Gyrase	absent	DNA topoisomerase (ATP-hydrolyzing) (gyrB)
	Recombination and repair	DNA recombination/ repair protein RecA	recB, recC, recD, Holliday junction resolvase RuvX, exodeoxyribonuclease I (sbcB)

Transcription	DNA-directed RNA polymerase (Rpo)	Rpo subunit β (broken genes for α and β’), σ70 (RpoD)	Rpo subunits α, β, β’ (rpoA, B, C), σ70 (RpoD)
	Elongation factor	absent	transcription elongation factor GreA
	Pausing factor	absent	transcription termination/ antitermination protein NusG
	Termination and maturation	Rho, possibly (43% positives with E. coli by tblastn)	transcription termination factor Rho, rRNA maturation RNase YbeY, rnc, rnpA, pnp
Translation	Ribosome biogenesis	absent	ribosome maturation factor RimM, rsmA, rsmD, rsmH, rlmB, ribosome biogenesis GTPase Der, RbfA, prmB, Obg family GTPase CgtA
	Initiation	translation initiation factors IF-1, IF-2 and IF-3	translation initiation factors IF-1, IF-2 and IF-3
	Elongation	elongation factors Tu and G, 50S ribosome-binding GTPase (MnmE), MnmG	elongation factors Ts, Tu, G and 4, translational GTPase TypA, rnpB, MiaB, MnmE
	Termination	peptide chain release factor 2, ribosome recycling factor	peptide chain release factor 1, peptide chain release factor 2, ribosome recycling factor, ribosome silencing factor (rsfS), SsrA-binding protein
	Aminoacyl-tRNA synthetase	alaS, aspS, gatA&gatB, glnS, gluRS, ileS, leuS, lysS, metRS, pheS, serS, trpS, tyrS, valS, tRNA lysidine(34) synthetase TilS, tRNA adenosine(34) deaminase TadA	alaS, asnS, aspS, gltX, glyS, glyQ, hisS, ileS, leuS, lysS, metRS, pheS, pheT, proS, serS, trpS, tyrS, valS, tRNA adenosine(34) deaminase TadA, tRNA lysidine(34) synthetase TilS
30S subunit	30S ribosomal proteins	S1, S2, S3, S4, S5, S7, S8, S9, S10, S11, S12, S13, S14, S15, S16, S17, S18, S19	S1, S2, S3, S4, S5, S6, S7, S8, S9, S10, S11, S12, S13, S14, S15, S16, S17, S18, S19, S21
50S subunit	50S ribosomal proteins	L1, L2, L3, L4, L5, L6, L7/L12, L10, L11, L13, L14, L15, L16, L17, L18, L21, L25, L27, L28, L31, L33, L35, L36	L1, L2, L3, L4, L5, L6, L7/12, L9, L10, L11, L13, L14, L15, L16, L17, L18, L19, L20, L21, L22, L23, L24, L27, L28, L29, L30, L31, L32, L33, L34, L35, L36, L3 N(5)-glutamine
Ribosomal functions		GTPase ObgE	YgfZ
Amino acid biosynthesis	valine biosynthesis	ketol-acid reductoisomerase, acetolactate synthase (ilvC EC1.1.1.86), branched-chain-amino-acid aminotransferase (ilvE EC2.6.1.42)	absent
	leucine biosynthesis	ketol-acid reductoisomerase, acetolactate synthase (ilvC EC1.1.1.86), aspartate-semialdehyde dehydrogenase(asd EC1.2.1.11), 2-isopropylmalate synthase(leuA EC2.3.3.13), 3-isopropylmalate dehydratase (leuC EC4.2.1.33), 3-isopropylmalate dehydrogenase(leuB EC1.1.1.85), branched-chain-amino-acid aminotransferase (ilvE EC2.6.1.42)	absent
	isoleucine biosynthesis	ketol-acid reductoisomerase (ilvC EC1.1.1.86, acetolactate synthase (ilvI EC2.2.1.6), aspartate-semialdehyde dehydrogenase (asd EC1.2.1.11), homoserine dehydrogenase (thrA EC1.1.3),	absent

	branched-chain-amino-acid aminotransferase (ilvE EC2.6.1.42)	
homoserine biosynthesis	aspartate kinase(lysC EC2.7.2.4), aspartate-semialdehyde dehydrogenase(asd EC1.2.1.11), homoserine dehydrogenase（thrA EC1.1.3）	absent
lysine biosynthesis	aspartate kinase(lysC EC2.7.2.4), aspartate-semialdehyde dehydrogenase(asd EC1.2.1.11), dihydrodipicolinate synthase (dapA EC4.3.3.7), dihydrodipicolinate reductase (dapB EC1.17.1.8), DAP-epimerase (dapF EC5.1.1.7), DAP-decarboxylase(lysA EC4.1.1.20), dapE (homolog); note: not identified tetrahydrodipicolinate N-succinyltransferase(dapD EC2.3.1.117), argD or serC homologs	absent
threonine biosynthesis	aspartate kinase (lysC EC2.7.2.4), homoserine dehydrogenase（thrA EC1.1.3, threonine synthase (thrC EC4.2.3.1)	absent
methionine synthesis	5-methyltetrahydropteroyl-triglutamate-- homocysteine S-methyltransferase (metE EC2.1.1.14)	absent
phenylalanine biosynthesis	3-phosphoshikimate 1-carboxyvinyltransferase (aroAEC2.5.1.19), shikimate kinase(aroK EC2.7.1.71), pheA (EC4.2.1.51), 3-deoxy-7-phosphoheptulonate synthase (aroH EC2.5.1.54)	absent
tryptophan biosynthesis	3-deoxy-7-phosphoheptulonate synthase (aroH EC2.5.1.54), anthranilate synthase (trpE EC4.1.3.27), phosphoribosylanthranilate isomerase (trpF EC5.3.1.24), tryptophan synthase (α & β EC4.2.1.20)	absent
tyrosine biosynthesis	3-deoxy-7-phosphoheptulonate synthase (aroH EC2.5.1.54)	absent
histidine biosynthesis	imidazoleglycerol-phosphate dehydratase (hisB EC:4.2.1.19), histidinol phosphate aminotransferase (hisC EC:2.6.1.9), imidazole glycerol phosphate synthase, glutamine amidotransferase subunit (hisH EC:4.3.2.10)	absent
arginine biosynthesis	argininosuccinate synthase (argG EC6.3.4.5), argininosuccinate lyase (argH EC4.3.2.1)	ornithine carbamoyltransferase (argF EC2.1.3.3), argininosuccinate synthase (argG EC6.3.4.5), argininosuccinate lyase (argH EC4.3.2.1)
asparagine and glutamine biosynthesis	absent	absent
glycine biosynthesis	serine hydroxymethyltransferase (glyA EC2.1.2.1)	absent

	glutamate and proline biosynthesis	bifunctional proline dehydrogenase/L-glutamate gamma-semialdehyde dehydrogenase PutA	absent
Chaperonins		dnaK, nucleotide exchange factor GrpE, groEL, groES, cold-shock protein	dnaK, DnaJ, groEL, groES, cold shock-like protein CspC, HtpG
Proteases		ClpP, ClpX (FtsH and HtpX absent)	ATP-dependent Clp endopeptidase subunits ClpP, ClpX, ATP-dependent zinc metalloprotease FtsH, protease HtpX
Enzyme modification		absent	lipoyl(octanoyl) transferase LipB
Enzyme cofactors	Riboflavin synthesis	absent	ribA [EC:3.5.4.25], ribB [EC:4.1.99.12], ribD [EC:3.5.4.26, 1.1.1.193], ribE [EC:2.5.1.78]
	Fe-S cluster assembly	absent	sufA, sufB, sufC, sufD, sufE, sufS
Antioxidants	glutathione biosynthesis	absent	glutamate--cysteine ligase (gshA)
Membrane lipid biosynthesis		cardiolipin synthase (cls)	cardiolipin synthase (cls), acyl carrier protein
Transport	transporters	absent	preprotein translocase subunits SecA, SecE, SecG and secY, YhgN family NAAT transporter, MFS transporter, inorganic phosphate transporter, CNNM family cation transport protein YoaE (magnesium/cobalt transporter CorA)
	cofactors	absent	signal recognition particle-docking protein, FtsY, signal recognition particle protein (ffh), signal recognition particle sRNA (ffs), signal peptidase II, membrane protein insertase yidC

List of full names of the identified genes encoding enzymes involved in the biosynthesis of essential amino acids, tricarboxylic acid cycle and DNA polymerase III:

argF= ornithine carbamoyltransferase [EC:2.1.3.3]
argG= argininosuccinate synthase [EC:6.3.4.5]
argH= argininosuccinate lyase [EC:4.3.2.1]
aroA= 3-phosphoshikimate 1-carboxyvinyltransferase [EC:2.5.1.19]
aroB= 3-dehydroquinate synthase [EC:4.2.3.4]
aroC= chorismate synthase [EC:4.2.3.5]
aroD= 3-dehydroquinate dehydratase [EC:4.2.1.10]
aroH= 3-deoxy-7-phosphoheptulonate synthase [EC:2.5.1.54]
asd= aspartate-semialdehyde dehydrogenase [EC:1.2.1.11]
carA= Carbamoyl phosphate synthase (small subunit) [EC:6.3.5.5]
carB= Carbamoyl phosphate synthase (large subunit) [EC:6.3.5.5], syn. pyrA
dapD= tetrahydrodipicolinate N-succinyltransferase [EC:2.3.1.117]
dapE= succinyl-diaminopimelate desuccinylase [EC=3.5.1.18]
dapF= diaminopimelate epimerase [EC:5.1.1.7]
deoD= Purine nucleoside phosphorylase DeoD-type [EC: 2.4.2.1]
dspA= dihydrodipicolinate synthase [EC:4.3.3.7]
dspB= dihydrodipicolinate reductase [EC:1.17.1.8]
hisA= 1-(5-phosphoribosyl)-5-[(5-phosphoribosylamino)methylideneamino] imidazole-4-carboxamide isomerase [EC:5.3.1.16]
hisB= imidazoleglycerol-phosphate dehydratase [EC:4.2.1.19]
hisC= histidinol phosphate aminotransferase [EC:2.6.1.9]
hisD= histidinol dehydrogenase [EC:1.1.1.23]
hisE= phosphoribosyl-ATP pyrophosphatase [EC:3.6.1.31]
hisF= imidazoleglycerol phosphate synthase, cyclase subunit [EC:4.3.2.10]
hisG= ATP phosphoribosyltransferase [EC:2.4.2.17]
hisH= imidazole glycerol phosphate synthase, glutamine amidotransferase subunit [EC:4.3.2.10]
hisI= phosphoribosyl-AMP cyclohydrolase [EC:3.5.4.19]
ilvC= ketol-acid reductoisomerase [EC:1.1.1.86]
ilvD= dihydroxy-acid dehydratase [EC:4.2.1.9]

ilvE= branched-chain amino acid aminotransferase [EC:2.6.1.42]
ilvI= acetolactate synthase large subunit [EC:2.2.1.6]
leuA= 2-isopropylmalate synthase [EC:2.3.3.13]
leuB= 3-isopropylmalate dehydrogenase [EC:1.1.1.85]
leuC= 3-isopropylmalate dehydratase [EC 4.2.1.33] = 3-isopropylmalate hydro-lyase
leuD= 3-isopropylmalate dehydratase small subunit [EC:4.2.1.33, 4.2.1.35]
lysA= diaminopimelate decarboxylase (decarboxylation of meso-diaminopimelate (meso-DAP) to L-lysine) [EC=4.1.1.20]
lysC= aspartokinase [EC:2.7.2.4]
metE= 5-methyltetrahydropteroyltriglutamate-- homocysteine S-methyltransferase [EC 2.1.1.14]
mqo= malate dehydrogenase, FAD/NAD(P)-binding domain [EC:1.1.5.4]
pdt= chorismate mutase [EC=5.4.99.5 4.2.1.51]
prs= ribose-phosphate pyrophosphokinase [EC: 2.7.6.1]
pyrB= aspartate carbamoyltransferase, catalytic subunit [EC:2.1.3.2]
pyrC= Dihydroorotase [EC:3.5.2.3]
pyrD= Dihydroorotate dehydrogenase [EC:1.3.5.2]
pyrF = Orotidine 5'-phosphate decarboxylase [EC:4.1.1.23]
pyrG= CTP synthase [EC 6.3.4.2], interconverts UTP and CTP
ribA= GTP cyclohydrolase II [EC:3.5.4.25]
ribB= 3,4 dihydroxy-2-butanone-4-phosphate synthase [EC:4.1.99.12]
ribD= fused diaminohydroxyphosphoribosylaminopyrimidine deaminase and 5-amino-6-(5-phosphoribosylamino) uracil reductase [EC:3.5.4.26 1.1.1.193]
ribE= riboflavin synthase beta chain [EC:2.5.1.78]
sucA= 2-oxoglutarate decarboxylase, thiamin-requiring [EC:1.2.4.2]
sucB= dihydrolipoyltranssuccinase [EC:2.3.1.61]
sucC= succinyl-CoA synthetase, beta subunit [EC:6.2.1.5]
sucD= succinyl-CoA synthetase, NAD(P)-binding, alpha subunit [EC:6.2.1.5]
thrA= bifunctional aspartokinase/ homoserine dehydrogenase [EC 1.1.1.3]
thrC= threonine synthase [EC:4.2.3.1]
thyA= thymidylate synthase [EC: 2.1.1.45]
trpA= tryptophan synthase subunit alpha [EC:4.2.1.20]
trpB= tryptophan synthase subunit beta [EC:4.2.1.20]
trpC= indole-3-glycerol phosphate synthase [EC:4.1.1.48]
trpE= anthranilate synthase component I [EC:4.1.3.27]
trpF= N-(5'-phosphoribosyl)anthranilate isomerase [EC:5.3.1.24]
trpG= anthranilate synthase component II [EC:4.1.3.27]

α(dnaE)= DNA polymerase III subunit alpha [EC:2.7.7.7]
β(dnaN)= beta sliding clamp [EC:2.7.7.7]
γ&τ(dnaX)= DNA polymerase III subunit tau [EC:2.7.7.7]
ε(dnaQ)= DNA polymerase III subunit epsilon [EC:2.7.7.7]
δ(holA)= DNA polymerase III subunit delta [EC:2.7.7.7]
δ'(holB)= DNA polymerase III subunit delta' [EC:2.7.7.7]
χ(holC)= DNA polymerase III subunit chi [EC:2.7.7.7]
Ψ(holD)= DNA polymerase III subunit psi [EC:2.7.7.7]
θ(holE)= DNA polymerase III subunit theta [EC:2.7.7.7]
