# Supplementary material for: Genomes of the Bacterial Endosymbionts of Carrot Psyllid Trioza apicalis Suggest Complementary Biosynthetic Capabilities
Source: Curr Microbiol. 2025 Feb 20;82(4):145. doi: 10.1007/s00284-025-04119-y (PMC11842425; doi:10.1007/s00284-025-04119-y)
Supplement: Supplementary file 5 — Supplementary file5 (PDF 2565 kb) [file 284_2025_4119_MOESM5_ESM.pdf]

Genomes of the bacterial endosymbionts of carrot psyllid *Trioza apicalis* suggest complementary biosynthetic capabilities

Current Microbiology

Sarah Thompson, Jinhui Wang, Thomas Schott, Riitta Nissinen, Minna Haapalainen

University of Helsinki

email: minna.haapalainen@helsinki.fi; minna.haapalainen@luke.fi

Supplementary Data S5. Genomic alignments by Mauve of the gamma proteobacterium endosymbiont of *Trioza apicalis* with closely-related bacterial endosymbionts for which the whole genome sequence was available: an ant endosymbiont '*Candidatus* Westeberhardia cardiocondylae' (GenBank LN774881), Enterobacteriaceae bacterium PSpicST1 (GenBank CP102982), an endosymbiont of *Cacopsylla picta,* and Enterobacteriaceae bacterium PC38 (GenBank CP102983), an endosymbiont of *Cacopsylla pyricola*.

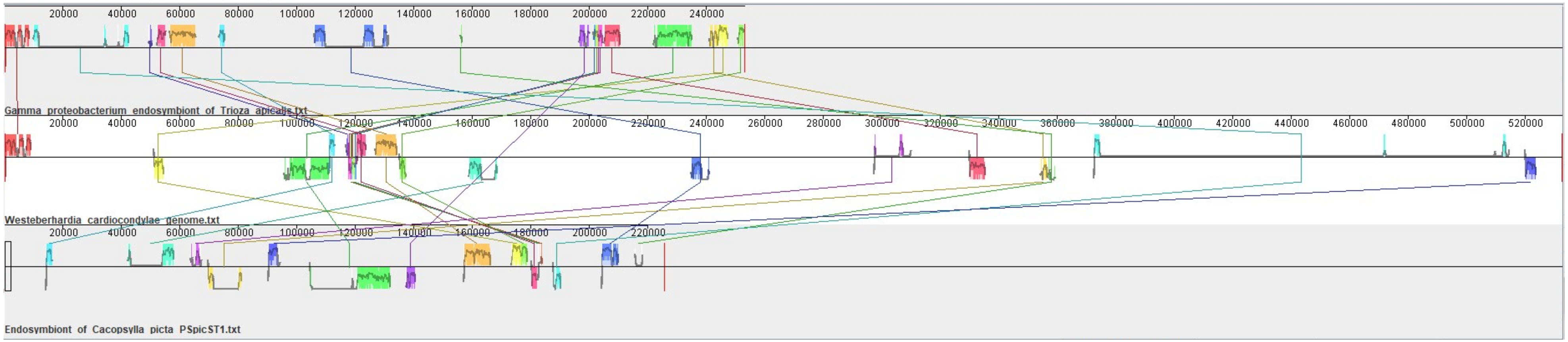

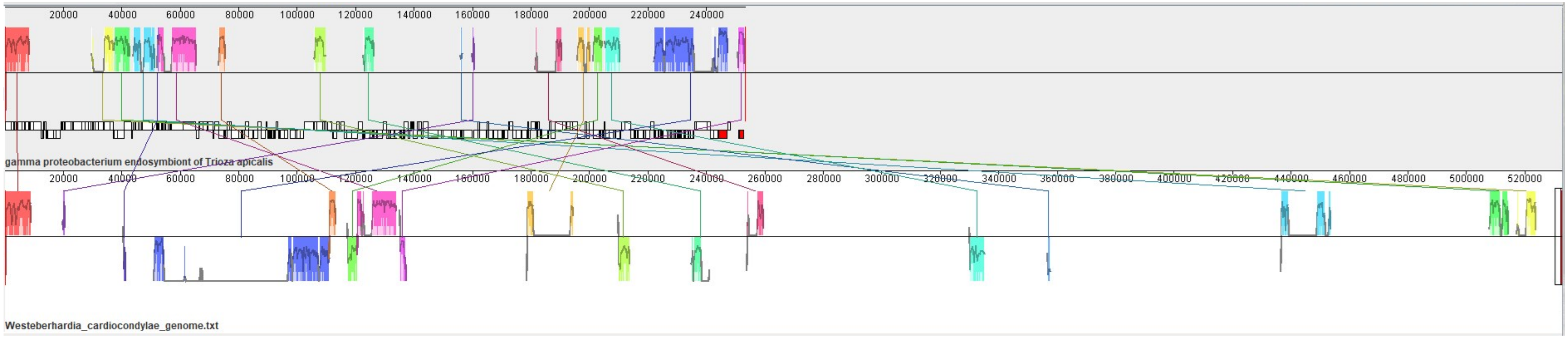
gamma proteobacterium endosymbiont of Trioza apicalis
Westeberhardia_cardiocondylae_genome.txt

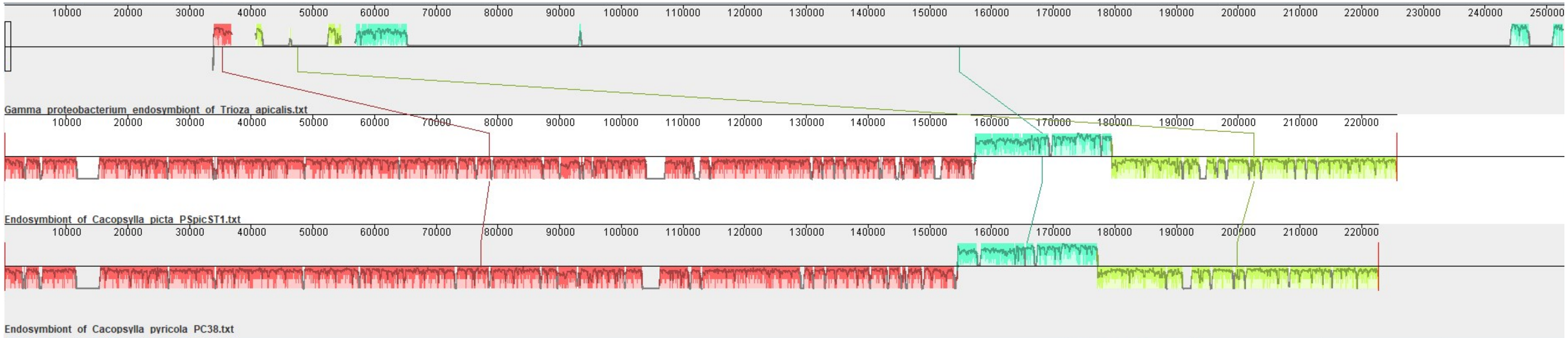
Gamma_proteobacterium_endosymbiont_of_Trioza_apicalis.txt
Endosymbiont_of_Cacopsylla_picta_PSpicST1.txt
Endosymbiont_of_Cacopsylla_pyricola_PC38.txt
