# Supplementary material for: Genomes of the Bacterial Endosymbionts of Carrot Psyllid Trioza apicalis Suggest Complementary Biosynthetic Capabilities
Source: Curr Microbiol. 2025 Feb 20;82(4):145. doi: 10.1007/s00284-025-04119-y (PMC11842425; doi:10.1007/s00284-025-04119-y)
Supplement: Supplementary file 8 — Supplementary file8 (PDF 169 kb) [file 284_2025_4119_MOESM8_ESM.pdf]

Genomes of the bacterial endosymbionts of carrot psyllid *Trioza apicalis* suggest complementary biosynthetic capabilities

Current Microbiology

Sarah Thompson, Jinhui Wang, Thomas Schott, Riitta Nissinen, Minna Haapalainen
University of Helsinki
email: minna.haapalainen@helsinki.fi; minna.haapalainen@luke.fi

Supplementary Data S8. 16S rRNA gene-based phylogeny of gammaproteobacteria endosymbionts of insects inferred by Maximum Likelihood method. Initial trees were obtained by applying Neighbor-Join and BioNJ algorithms to a matrix of pairwise distances. The percentage of trees in which the associated taxa clustered together is shown next to the branches. The tree is drawn to scale, with branch lengths measured in the number of substitutions per site, as indicated by the scale bar. *Agrobacterium tumefaciens* (alphaproteobacteria) type strain ATCC 4720 was used as the outgroup.

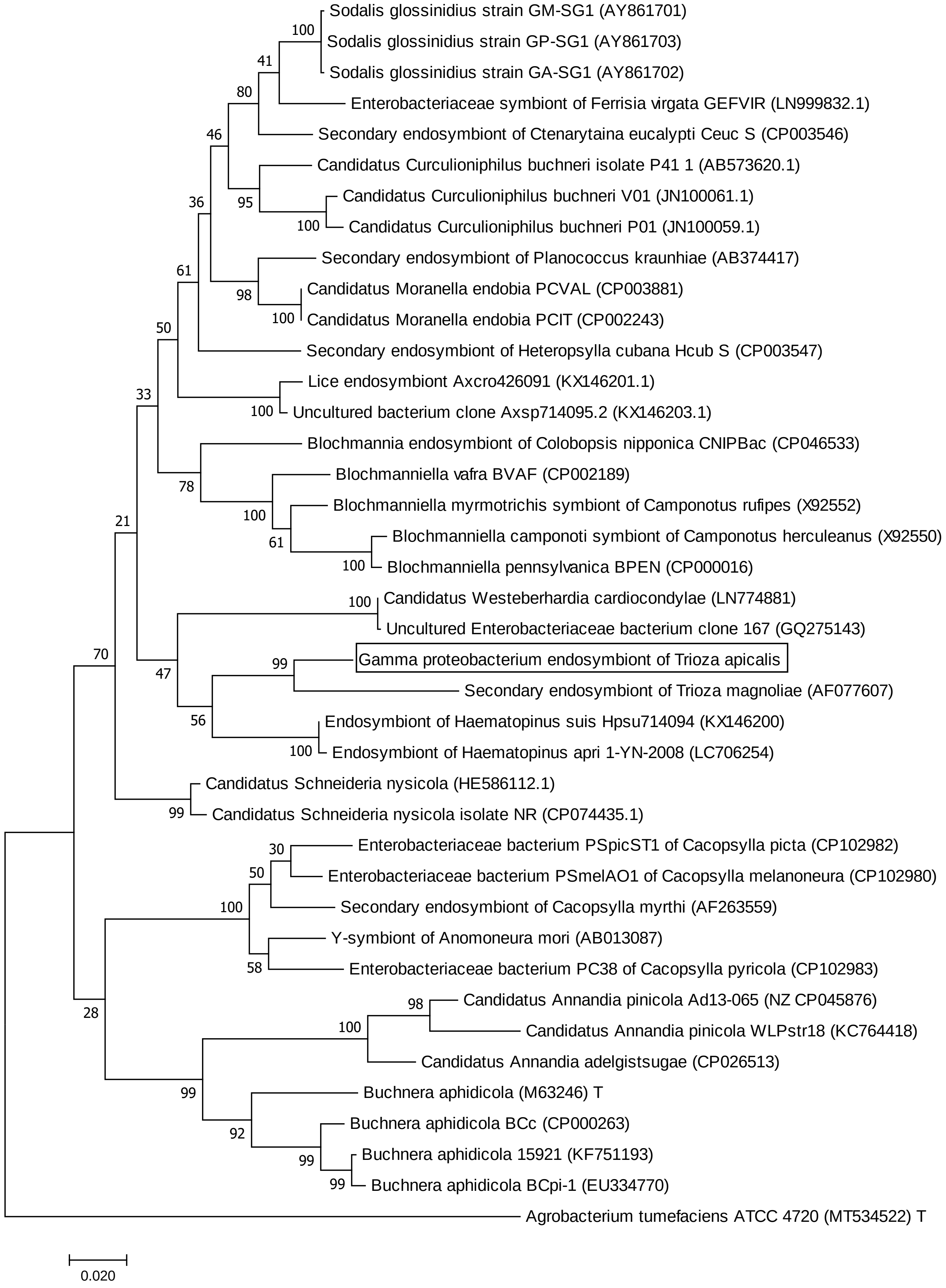
Sodalis glossinidius strain GM-SG1 (AY861701)
Sodalis glossinidius strain GP-SG1 (AY861703)
Sodalis glossinidius strain GA-SG1 (AY861702)
Enterobacteriaceae symbiont of Ferrisia virgata GEFVIR (LN999832.1)
Secondary endosymbiont of Ctenarytaina eucalypti Ceuc S (CP003546)
Candidatus Curculioniphilus buchneri isolate P41 1 (AB573620.1)
Candidatus Curculioniphilus buchneri V01 (JN100061.1)
Candidatus Curculioniphilus buchneri P01 (JN100059.1)
Secondary endosymbiont of Planococcus kraunhiae (AB374417)
Candidatus Moranella endobia PCVAL (CP003881)
Candidatus Moranella endobia PCIT (CP002243)
Secondary endosymbiont of Heteropsylla cubana Hcub S (CP003547)
Lice endosymbiont Axcro426091 (KX146201.1)
Uncultured bacterium clone Axsp714095.2 (KX146203.1)
Blochmannia endosymbiont of Colobopsis nipponica CNIPBac (CP046533)
Blochmanniella vafra BVAF (CP002189)
Blochmanniella myrmotrichis symbiont of Camponotus rufipes (X92552)
Blochmanniella camponoti symbiont of Camponotus herculeanus (X92550)
Blochmanniella pennsylvanica BPEN (CP000016)
Candidatus Westeberhardia cardiocondylae (LN774881)
Uncultured Enterobacteriaceae bacterium clone 167 (GQ275143)
Gamma proteobacterium endosymbiont of Trioza apicalis
Secondary endosymbiont of Trioza magnoliae (AF077607)
Endosymbiont of Haematopinus suis Hpsu714094 (KX146200)
Endosymbiont of Haematopinus apri 1-YN-2008 (LC706254)
Candidatus Schneideria nysicola (HE586112.1)
Candidatus Schneideria nysicola isolate NR (CP074435.1)
Enterobacteriaceae bacterium PSpicST1 of Cacopsylla picta (CP102982)
Enterobacteriaceae bacterium PSmelAO1 of Cacopsylla melanoneura (CP102980)
Secondary endosymbiont of Cacopsylla myrthi (AF263559)
Y-symbiont of Anomoneura mori (AB013087)
Enterobacteriaceae bacterium PC38 of Cacopsylla pyricola (CP102983)
Candidatus Annandia pinicola Ad13-065 (NZ CP045876)
Candidatus Annandia pinicola WLPstr18 (KC764418)
Candidatus Annandia adelgistsugae (CP026513)
Buchnera aphidicola (M63246) T
Buchnera aphidicola BCc (CP000263)
Buchnera aphidicola 15921 (KF751193)
Buchnera aphidicola BCpi-1 (EU334770)
Agrobacterium tumefaciens ATCC 4720 (MT534522) T
100
41
80
46
95
100
36
61
98
100
50
33
100
78
100
61
100
21
100
47
99
56
100
70
99
30
50
100
58
98
100
28
99
92
99
99
0.020
